# Supplementary material for: The fruit fly acetyltransferase chameau promotes starvation resilience at the expense of longevity
Source: EMBO Rep. 2023 Sep 19;24(10):e57023. doi: 10.15252/embr.202357023 (PMC10561354; doi:10.15252/embr.202357023)
Supplement: Supplementary file 1 — Appendix S1 [file EMBR-24-e57023-s008.pdf]

Table of contents

Appendix Figure S1 1
Appendix Figure S2 2

Appendix Figure S1

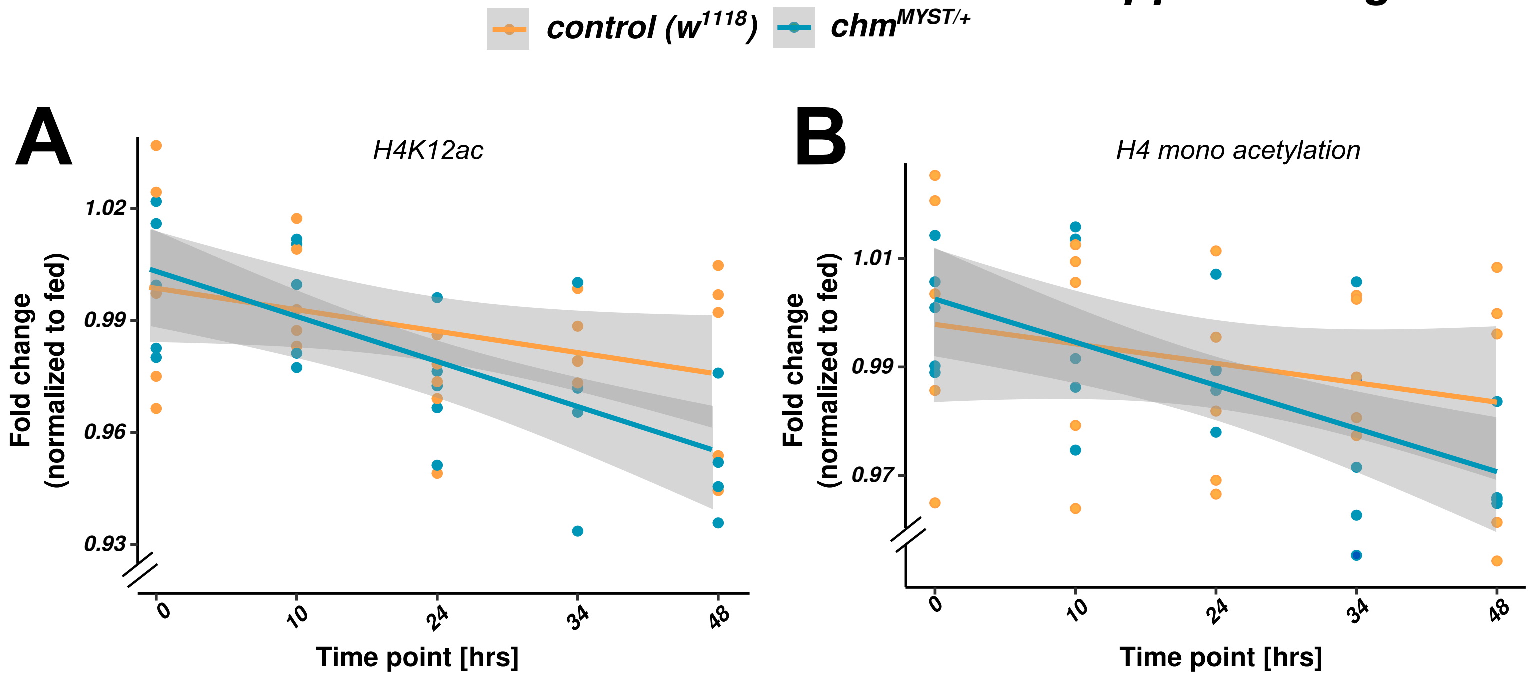

Appendix Figure S1: *Histone acetylation shows a decreasing trend in both control and $chm^{MYST/+}$ flies upon starvation.* Linear model showing mass-spectrometry quantified levels of A) H4K12ac and B) H4 mono-acetylation in control (w^{1118}) and $chm^{MYST/+}$ male flies across different starvation time points. Relative percentages of histone PTM were normalized to fed of corresponding genotype (N = 5, unpaired). Non-significant values are not indicated.

Appendix Figure S2

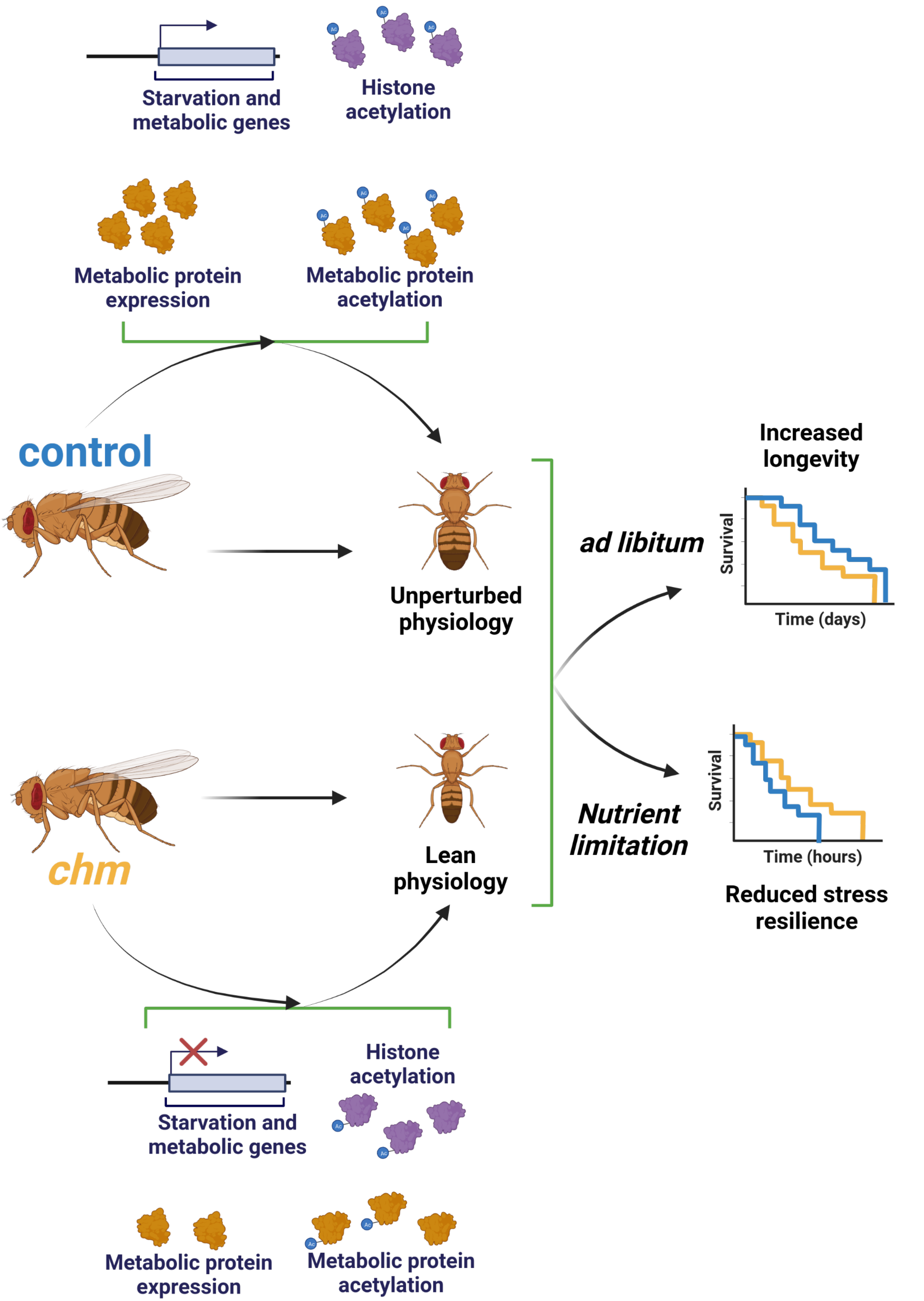

Appendix Figure S2: A decreased chm activity results in a lean phenotype and down regulation of genes and proteins involved in metabolic processes. While this results in life time extension in times of plenty, it is detrimental when food is sparse.
