# Supplementary material for: The fruit fly acetyltransferase chameau promotes starvation resilience at the expense of longevity
Source: EMBO Rep. 2023 Sep 19;24(10):e57023. doi: 10.15252/embr.202357023 (PMC10561354; doi:10.15252/embr.202357023)
Supplement: Supplementary file 2 — Expanded View Figures PDF [file EMBR-24-e57023-s003.pdf]

Expanded View Figures

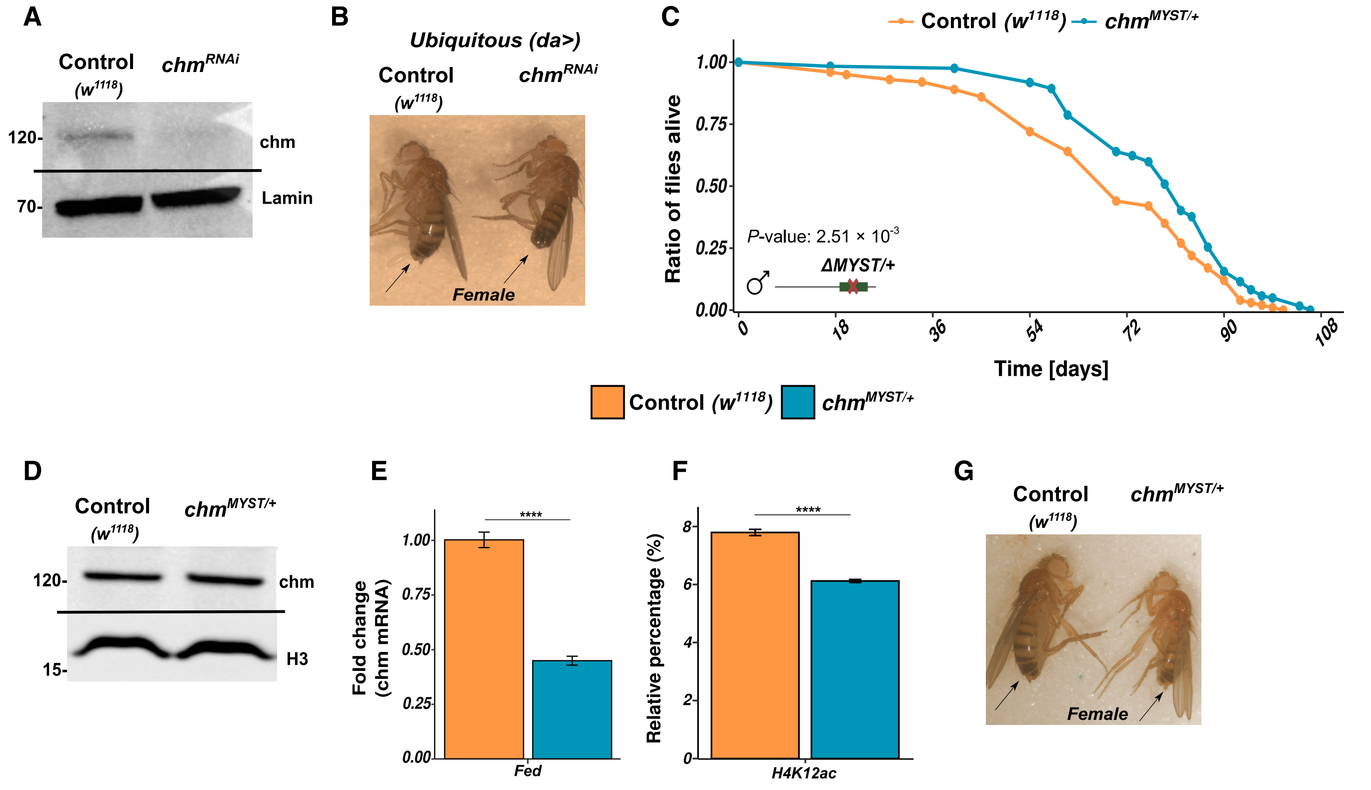

Figure EV1. Longevity in males is unaffected by temperature and female *chm* mutants also show changes in physiology.

A Western blot of control (*ω*^{1118}) and *chm*^{RNAi}.

B Image of 8- to 9-day-old female control (*ω*^{1118}, left) and *chm*^{RNAi} (right) flies upon ubiquitous *chm* knockdown.

C Survival curve showing increased lifespan of *chm*$^{MYST/+}$ flies at 23°C (N = 1).

D Western blot of control (w^{1118}) and *chm*$^{MYST/+}$.

E mRNA levels of *chm* in control (*ω*^{1118}) and *chm*$^{MYST/+}$ flies (N = 4 (control) and 5 (*chm*$^{MYST/+}$), unpaired).

F Mass spectrometry quantified relative percentage of H4K12ac between control (*ω*^{1118}) and *chm*$^{MYST/+}$ flies (N = 5, unpaired).

G Image of 8- to 9-day-old female control (*ω*^{1118}, left) and *chm*$^{MYST/+}$ (right) flies.

Data information: All replicates are independent biological replicates and error bars indicate standard error of the mean (SEM). Unpaired *t*-test was performed and non-significant values are not shown (*P < 0.05, **P < 0.01, ***P < 0.001, ****P < 0.0001). For survival curves, log-rank test was performed.
Source data are available online for this figure.

 © 2023 The Authors

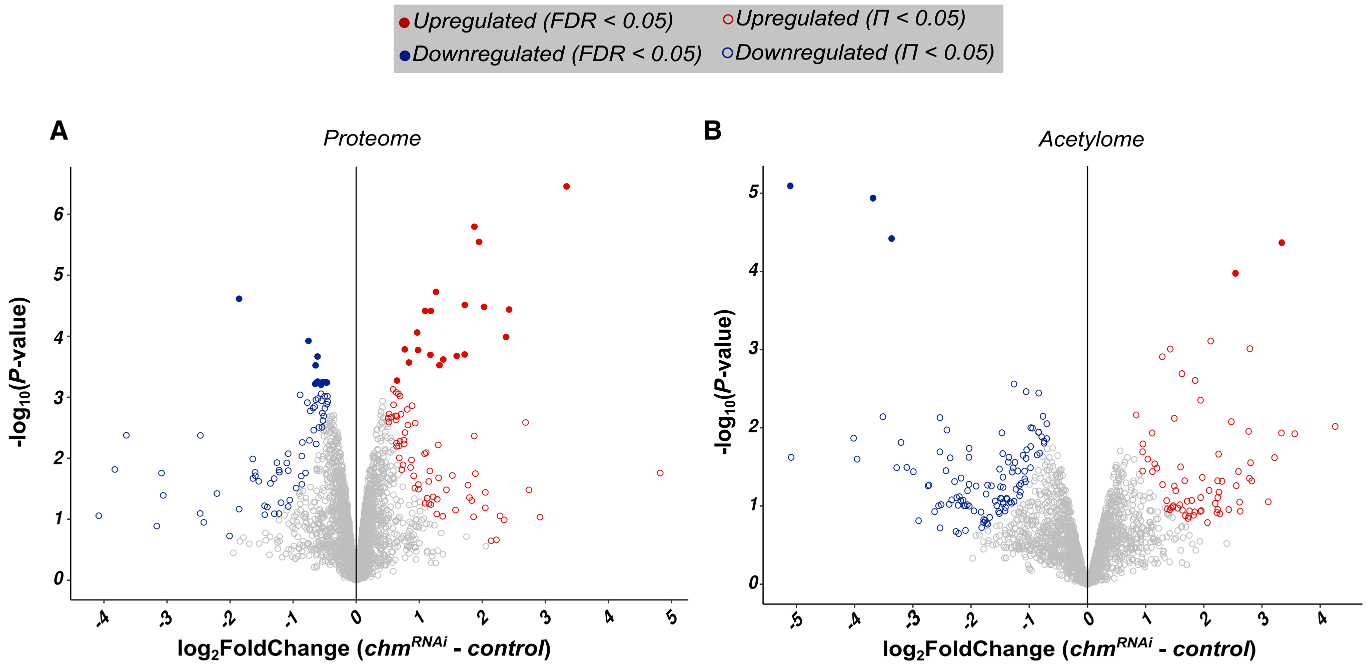

Figure EV2. Proteome and acetylome data show significant differential changes upon *chm* knockdown.

A, B Volcano plot showing the $\log_2$Foldchange (chm^{RNAi} - control) in x-axis and $\log_{10}$*P*-value in y-axis for A) proteome and B) acetylome (not normalized to the proteome). Filled circles indicate FDR < 0.05 and unfilled circles indicate $\Pi < 0.05$. Color of the circles indicates the nature of differential regulation.

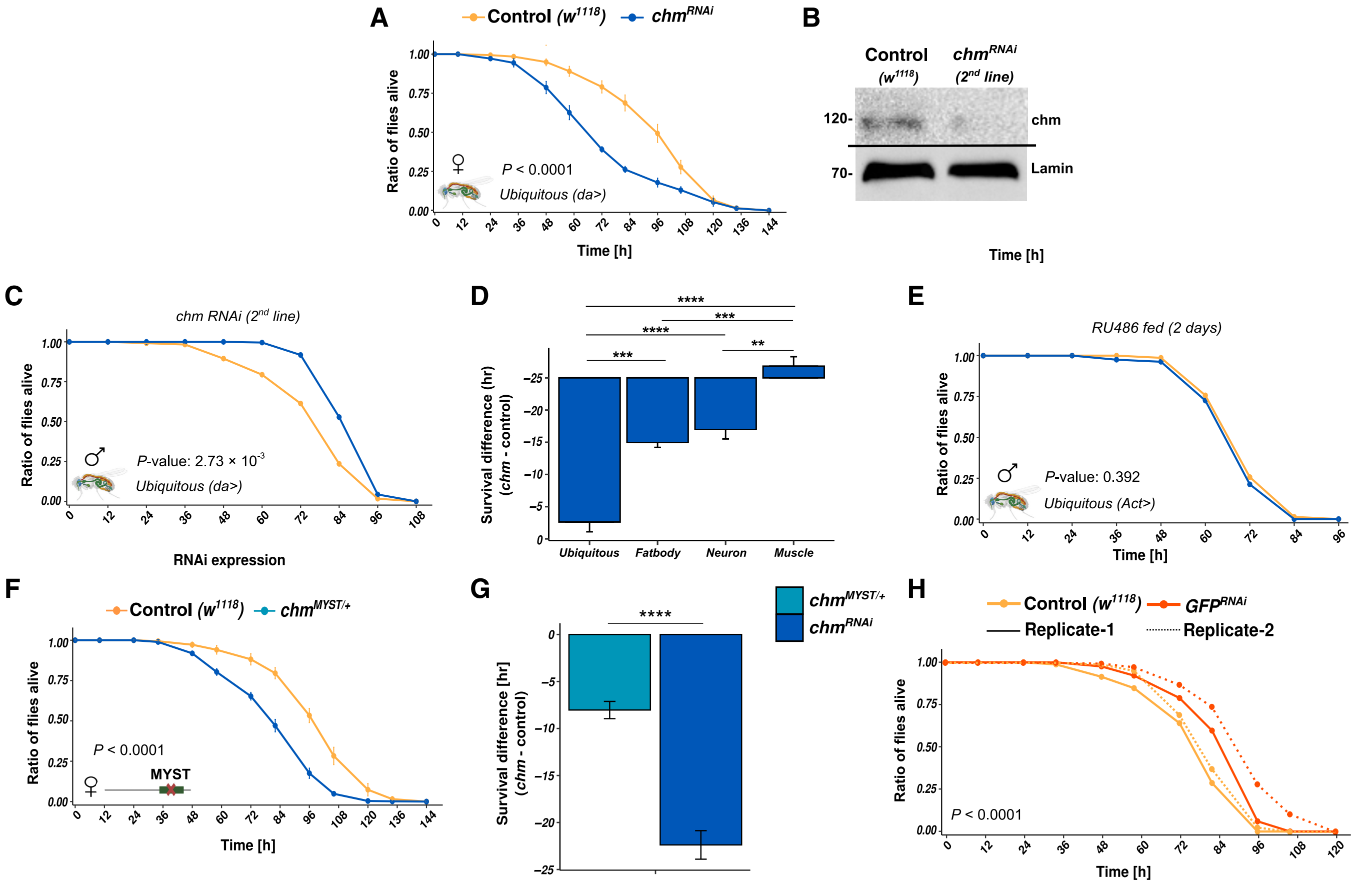

Figure EV3. Starvation susceptibility in chm mutants is observed independent of gender and RNAi line but not upon 2-day RU486 treatment.

A Average survival curve of control (w^{1118}) and chm^{RNAi} female flies upon ubiquitous knockdown of *chm* with *da-Gal4* (N = 4, paired).

B Western blot of chm for control (w^{1118}) and $chm^{RNAi\text{-}2}$.

C Survival curve of control (w^{1118}) and $chm^{RNAi\text{-}2}$ male flies upon ubiquitous knockdown of *chm* with *da-Gal4* (N = 1).

D Survival difference of chm knockdown using different drivers (ANOVA P-value: 2.73 × 10^{-07}, N = 3 (neuron- and muscle-specific) and 4 (ubiquitous and fat body-specific); Tukey *post-hoc* test was performed for statistical significance).

E Survival curve of control (w^{1118}) and chm^{RNAi} male flies upon RU486 administration for 2 days before starvation (N = 1).

F Average survival curve of control (w^{1118}) and $chm^{MYST/+}$ female flies (N = 4, paired).

G Survival difference between ubiquitous chm^{RNAi} and $chm^{MYST/+}$ male flies (N = 4 (RNAi) and 5 (MYST/+), unpaired t-test was performed for statistical significance).

H Survival curves of control (w^{1118}) and GFP^{RNAi} male flies upon ubiquitous GFP expression with *da-Gal4* (N = 2, paired). Each independent biological replicate is indicated by the solid and dotted lines.

Data information: All replicates are independent biological replicates and error bars indicate standard error of the mean (SEM). Unpaired t-test or Tukey test was performed as indicated and non-significant values are not shown (*P < 0.05, **P < 0.01, ***P < 0.001, ****P < 0.0001). For survival curves, log-rank test was performed for each biological replicate. The displayed P-value is based on all biological replicates.
Source data are available online for this figure.

 © 2023 The Authors

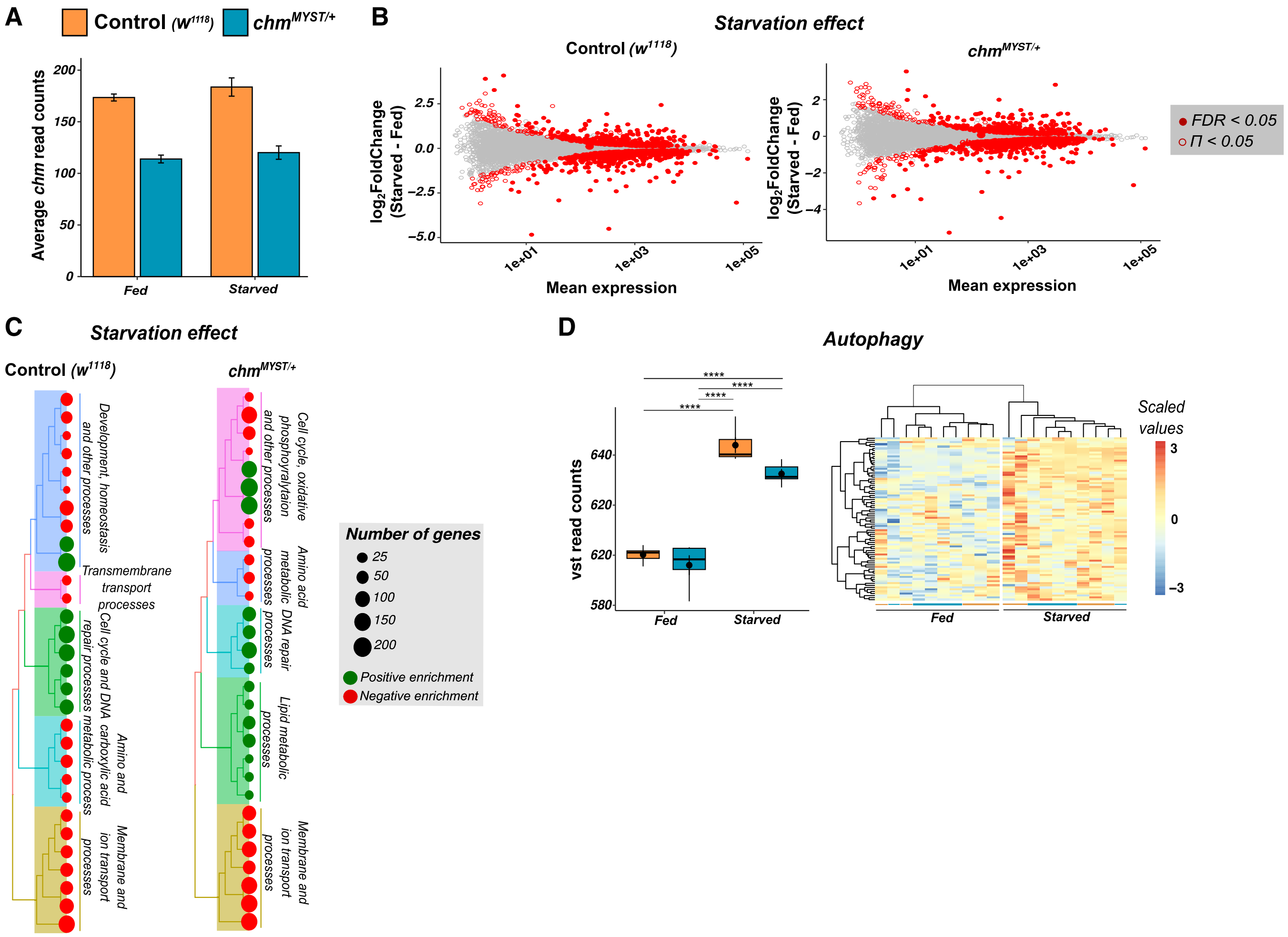

Figure EV4. Transcriptomic data show enrichment of similar genes and GO terms upon starvation independent of the genotype.

A Bar plot showing the average read counts of *chm* at fed and starved in control (w^{1118}) and $chm^{MYST/+}$ flies ($N = 5$, unpaired).

B MA plot of control (w^{1118}) and $chm^{MYST/+}$ flies in response to starvation. Significant genes are highlighted in filled and unfilled red circles for FDR < 0.05 and $\Pi < 0.05$, respectively.

C Tree plot depicting top 30 significant GO terms of starvation effect from GSEA of control (w^{1118}) and $chm^{MYST/+}$ transcriptome. Color on the circles indicates enrichment and the size indicates number of genes annotated with that pathway. GO terms were clustered based on semantic similarity and the terms that were represented the most within a cluster were mentioned.

D Box plot (left) and heatmap (right) of annotated genes from GO term autophagy (ANOVA *P*-value: 2.10×10^{-09}). Box plot shows the average vst normalized read counts of all annotated genes within the GO term averaged over the replicates. Boxes indicate interquartile range with central band as the median, central filled dot as the average, and the whiskers indicating the maximum and minimum values across five biological replicates. Heatmap shows the scaled values of vst normalized read counts in each replicate for all conditions.

Data information: All replicates are independent biological replicates and error bars indicate standard error of the mean (SEM). ANOVA followed by Tukey test was performed for box plot, and non-significant values are not shown (**P* < 0.05, ***P* < 0.01, ****P* < 0.001, *****P* < 0.0001).
Source data are available online for this figure.

© 2023 The Authors

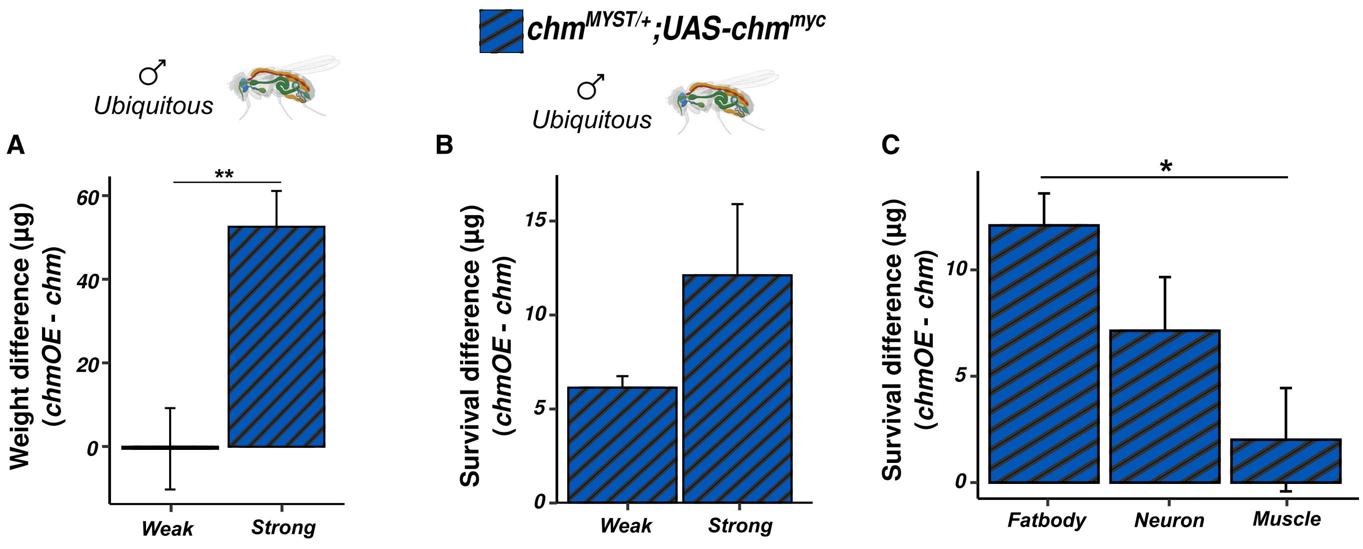

Figure EV5. chm's function in starvation susceptibility is validated by improvement of the phenotype upon overexpression.

A Weight difference in *chm*$^{MYST/+}$;*UAS-chm*^{myc} between weak and strong ubiquitous overexpressing male flies (*N* = 4 (strong) and 5 (weak), unpaired *t*-test was performed for statistical significance).

B Survival difference in *chm*$^{MYST/+}$;*UAS-chm*^{myc} between weak and strong ubiquitous overexpressing male flies (*N* = 3 (strong) and 4 (weak), unpaired *t*-test was performed for statistical significance).

C Survival difference in *chm*$^{MYST/+}$;*UAS-chm*^{myc} between different tissue-specific overexpressing male flies (*N* = 4 (fat body-specific) and 5 (neuron- and muscle-specific), unpaired, ANOVA *P*-value: 0.036; Tukey *post-hoc* test was performed for statistical significance).

Data information: All replicates are independent biological replicates and error bars indicate standard error of the mean (SEM). Unpaired *t*-test or Tukey test was performed as indicated and non-significant values are not shown (*P < 0.05, **P < 0.01, ***P < 0.001, ****P < 0.0001).
Source data are available online for this figure.

 © 2023 The Authors
