# Supplementary material for: The fruit fly acetyltransferase chameau promotes starvation resilience at the expense of longevity
Source: EMBO Rep. 2023 Sep 19;24(10):e57023. doi: 10.15252/embr.202357023 (PMC10561354; doi:10.15252/embr.202357023)
Supplement: Supplementary file 14 — PDF+ [file EMBR-24-e57023-s012.pdf]

Article

SOURCE DATA | TRANSPARENT PROCESS | OPEN ACCESS

EMBO reports

The fruit fly acetyltransferase chameau promotes starvation resilience at the expense of longevity

Anuroop Venkateswaran Venkatasubramani[1,2], Toshiharu Ichinose[3,4], Mai Kanno[3], Ignasi Forne[5], Hiromu Tanimoto[3], Shahaf Peleg[6,*] & Axel Imhof[1,5,**]

Abstract

Proteins involved in cellular metabolism and molecular regulation can extend lifespan of various organisms in the laboratory. However, any improvement in aging would only provide an evolutionary benefit if the organisms were able to survive under non-ideal conditions. We have previously shown that *Drosophila melanogaster* carrying a loss-of-function allele of the acetyltransferase *chameau* (*chm*) has an increased healthy lifespan when fed *ad libitum*. Here, we show that loss of chm and reduction in its activity results in a substantial reduction in weight and a decrease in starvation resistance. This phenotype is caused by failure to properly regulate the genes and proteins required for energy storage and expenditure. The previously observed increase in survival time thus comes with the inability to prepare for and cope with nutrient stress. As the ability to survive in environments with restricted food availability is likely a stronger evolutionary driver than the ability to live a long life, chm is still present in the organism's genome despite its apparent negative effect on lifespan.

Keywords acetylation; chameau; *Drosophila*; HBO1; starvation
Subject Categories Chromatin, Transcription, & Genomics; Metabolism; Post-translational Modifications & Proteolysis
DOI 10.15252/embr.202357023 | Received 22 February 2023 | Revised 22 August 2023 | Accepted 25 August 2023 | Published online 19 September 2023
EMBO Reports (2023) 24: e57023

Introduction

Organisms respond to a changing environment by complex physiological and behavioral adaptations (Flatt, 2020). These are triggered by changes in gene expression or metabolism. The availability of nutrients is one of the most dynamically changing extrinsic parameters. Therefore, a tight regulation of nutrient storage and energy expenditure is essential for survival (Zinke *et al*, 2002; Hsieh *et al*, 2022). While hormonal regulation of metabolism at an organismic level is well established, the existence of intracellular feedback loops that regulate storage and usage of metabolites is largely underexplored. Metabolic pathways are often regulated by positive- or negative-feedback loops mediated by key metabolites. While these loops result in fast changes in metabolic fluxes, organisms can also adapt to long-term changes in nutrient availability by transcriptional and posttranscriptional regulation of key enzymes (Carthew, 2021). The coupling of metabolite concentrations to transcriptional regulators has been suggested to constitute an important and yet understudied principle of regulation (Katada *et al*, 2012). Several transcription factors have been shown to respond to nutrients via the mTOR pathway, cAMP signaling, or even by direct binding of metabolites to transcription factors. However, nutritional changes can also result in an altered activity of enzymes or transcription factors by changing their posttranslational modification status. Such modifications can result not only in transient changes in the metabolic flux in case of enzymes (Shi & Tu, 2015; Ye & Tu, 2018; Hsieh *et al*, 2022) but also in alterations of gene expression programs that result in more sustainable adaptation (Rodrigues *et al*, 2021). The latter is often affected by modifications of histones or sequence-specific transcription factors. In fact, multiple transcription factors, such as C/EBPα (Zaini *et al*, 2018), PGC1 (Jeninga *et al*, 2010), (spargel), or dFoxo (Molaei *et al*, 2019), have been shown to be dynamically acetylated upon metabolic variation.

Chameau (chm) is one of four MYST domain-containing acetyltransferases in *Drosophila* and is involved in the specification of replication origins and in JNK/AP-1-dependent transcription during fly development (Aggarwal & Calvi, 2004; Miotto *et al*, 2006; McConnell *et al*, 2012). Its human ortholog HBO1 (HAT bound to ORC1; Grienenberger *et al*, 2002) also plays a role in DNA replication (Iizuka & Stillman, 1999) and JNK-mediated transcriptional activation (Miotto *et al*, 2006). Moreover, HBO1 and chm are also involved in metabolic reprogramming metastasizing colorectal cancer cells (Wu *et al*, 2015) and during the differentiation of hematopoietic progenitors (Tiwari *et al*, 2020), respectively. Consistent

1 Department of Molecular Biology, Biomedical Center Munich, Faculty of Medicine, LMU Munich, Martinsried, Germany
2 Graduate School of Quantitative Biosciences (QBM), LMU Munich, Munich, Germany
3 Graduate School of Life Sciences, Tohoku University, Sendai, Japan
4 Frontier Research Institute for Interdisciplinary Sciences, Tohoku University, Sendai, Japan
5 Protein Analysis Unit, Faculty of Medicine, Biomedical Center Munich, LMU Munich, Martinsried, Germany
6 Research Group Epigenetics, Metabolism and Longevity, Institute for Farm Animal Biology, Dummerstorf, Germany
*Corresponding author. Tel: +49 089 2180 75420; E-mail: imhof@lmu.de
**Corresponding author. Tel: +49 38208 68 942; E-mail: peleg@fbn-dummerstorf.de

© 2023 The Authors. Published under the terms of the CC BY 4.0 license.

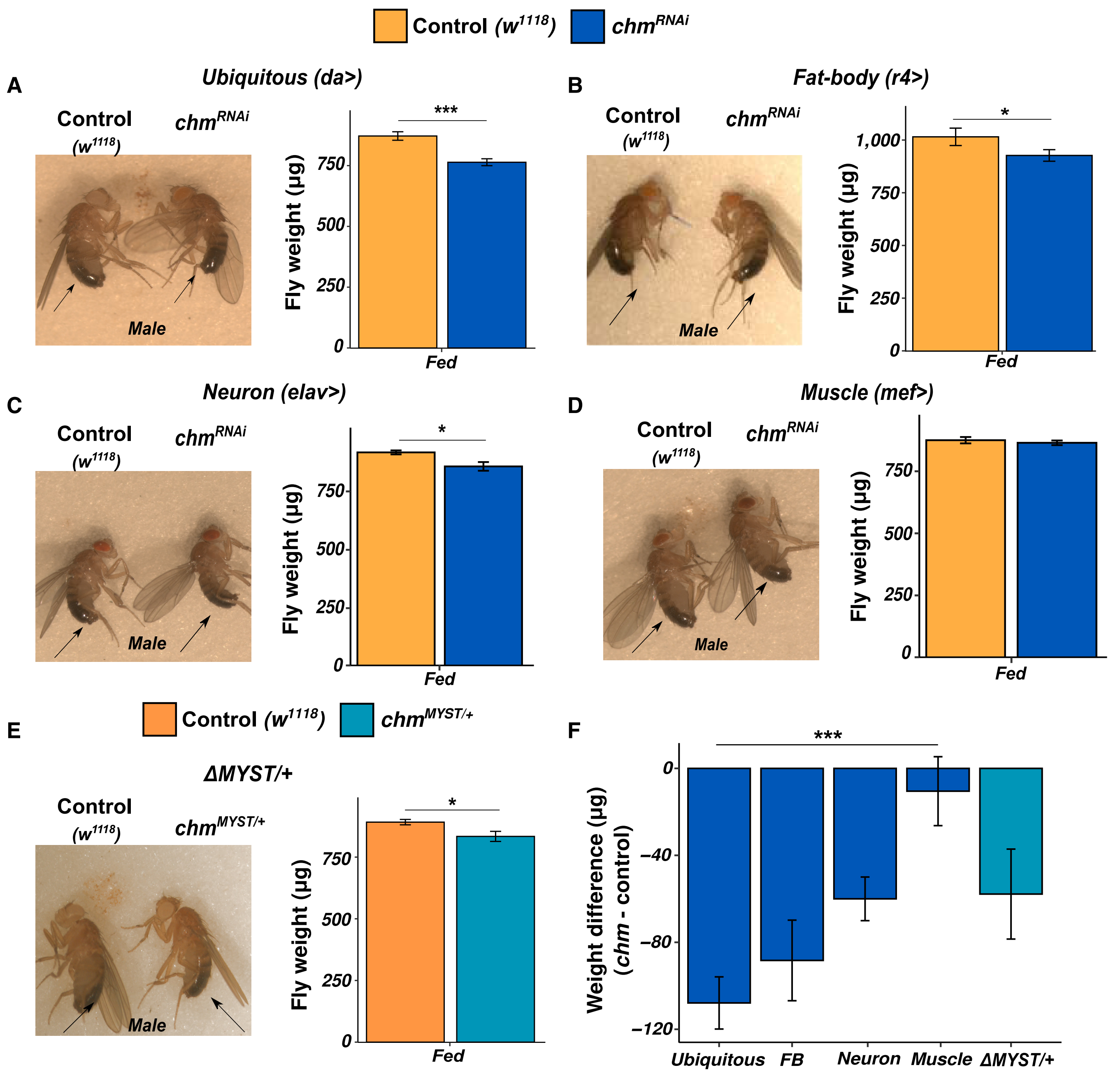

Figure 1. Loss of chm and reduction in its activity results in physiological changes in weight/size.

A Image of 8- to 9-day-old male control (w^{1118}, left) and chm^{RNAi} (right) flies upon ubiquitous chm knockdown (left) and the corresponding quantification (right; N = 6, paired t-test was performed for statistical significance).

B Image of 8- to 9-day-old male control (w^{1118}, left) and chm^{RNAi} (right) flies upon fat body–specific chm knockdown (left) and the corresponding quantification (right; N = 4, paired t-test was performed for statistical significance).

C Image of 8- to 9-day-old male control (w^{1118}, left) and chm^{RNAi} (right) flies upon neuron-specific chm knockdown (left) and the corresponding quantification (right; N = 3, paired t-test was performed for statistical significance).

D Image of 8- to 9-day-old male control (w^{1118}, left) and chm^{RNAi} (right) flies upon muscle-specific chm knockdown (left) and the corresponding quantification (right; N = 3, paired t-test was performed for statistical significance).

E Image of 8- to 9-day-old male control (w^{1118}, left) and $chm^{MYST/+}$ (right) flies (left) and the corresponding quantification (right; N = 5, paired t-test was performed for statistical significance).

F Comparison of weight difference between enzymatic mutant and RNAi flies with varying specificity of chm knockdown (N = 3 (neuron- and muscle-specific RNAi), 4 (fat body-specific RNAi), 5 (MYST/+ mutants), and 6 (ubiquitous RNAi); Tukey *post-hoc* test was performed for statistical significance, ANOVA P-value = 0.012).

Data information: All replicates are independent biological replicates and error bars indicate standard error of the mean (SEM). Paired t-test or Tukey test was performed as indicated and non-significant values are not shown (*$P < 0.05$, **$P < 0.01$, ***$P < 0.001$, ****$P < 0.0001$).
Source data are available online for this figure.

 © 2023 The Authors

with the hypothesis that HBO1/chm evolved to sense environmental changes, *chm* exhibits a strong sequence variation between tropical and temperate populations of *Drosophila melanogaster* (Levine & Begun, 2008). A homozygous deletion of *chm* in *Drosophila* results in pupal lethality of the organism (Grienenberger *et al*, 2002) but a partial reduction in *chm* in *Drosophila* or *HBO1* in mice extends lifespan (Peleg *et al*, 2016a; Wang *et al*, 2021) when animals are fed *ad libitum*. In *Drosophila*, this lifespan extension is caused by a lower responsiveness of *chm* mutant flies to potentially dangerously increased levels of acetyl-CoA in aging animals (Peleg *et al*, 2016a). Therefore, we wondered whether the ability of flies to cope with longer lifespan of *chm* mutant flies is accompanied by an impaired response during conditions of limiting nutrients.

Results

Loss of chameau (chm) results in a substantial weight loss in *Drosophila melanogaster*

To investigate chm's physiological function in adult flies, we reduced chm levels by crossing flies carrying an UAS-driven *chm* RNAi transgene with a ubiquitous *da-Gal4* driver line (Fig EV1A). In addition to a fewer number of eclosed flies, loss of chm resulted in a reduction in body weight and size of both male and female flies as compared to the isogenic control flies of the same age or a control RNAi line (Figs 1A and EV1B). As *chm* is highly expressed in neuronal tissue and in fat body (Jenkins *et al*, 2022), we selectively removed *chm* from these tissues by crossing the RNAi flies with the corresponding driver lines (*elav-Gal4* for neurons and *r4-Gal4* for fat body). While we did not observe an effect on fly development, a reduction in *chm* expression in neurons as well as in fat body showed a substantial loss in size and weight (Fig 1B and C). However, when crossed with *mef-Gal4* (muscle-specific driver), no difference in size or weight was observed between control and RNAi flies (Fig 1D).

Enzymatic activity of chm is important for the organisms' normal physiology

Since chm is an acetyltransferase, we wanted to assess if its enzymatic activity is important for this phenotype. To do this, we used a fly line heterozygous for a mutant allele with partial deletion of the MYST domain (Grienenberger *et al*, 2002), referred to as $chm^{MYST/+}$. While flies homozygous for this allele do not survive, heterozygous $chm^{MYST/+}$ flies are viable and even show an extended lifespan independent of the ambient temperature (Peleg *et al*, 2016a; Fig EV1C). The mutant allele did not affect the protein levels of chm (Fig EV1D), but a qPCR assay using primers hybridizing with the sequence of the mutated MYST domain showed a 50% reduction (Fig EV1E). Consistent with the reduced chm activity, a reduced bulk level of H4K12ac was measured by mass spectrometry (Feller *et al*, 2015; Peleg *et al*, 2016a; Fig EV1F). The heterozygous deletion mutant did not show any noticeable difference in development but showed a significant weight reduction compared to the controls of same age (Figs 1E and EV1G), indicating that the acetyltransferase activity of chm is important for the organism's physiology. Interestingly, this phenotype is only observed at temperatures of 23°C and not higher, which is why we performed all following experiments at 23°C.

In summary, lack of chm and its activity affects the physiology of flies as indicated by the thin phenotype. This effect is mainly caused by the expression of active chm in the fat body or neurons (Fig 1F).

Transcriptomic analysis predicts the role of chm in regulation of starvation

As $chm^{MYST/+}$ flies have reduced histone acetylation levels, we decided to assess the transcriptome of these flies. Principle component analysis (PCA) and a Spearman correlation clustering of the transcriptome separate into two clear clusters indicating the two genotypes but (Fig 2A and B) only few individual genes showed significantly different expression levels (Fig 2C; Dataset EV1). To check whether the moderate effect of chm on individual genes may nevertheless result in cumulative effect on entire pathways, we performed a gene set enrichment analysis (GSEA) on the data set. This analysis revealed a significant enrichment and downregulation of most biological processes related to metabolism (Fig 2D; Dataset EV2). Interestingly, and consistent with the observed weight reduction, genes involved in processes such as response to starvation and maintenance of nutrient levels were also downregulated in $chm^{MYST/+}$ flies (Fig 2E). These observations suggest a modulatory role of chm in response to nutrient-limiting conditions by affecting the expression of genes involved in metabolic pathways.

Loss of chm reduces proteome and acetylome of metabolic proteins

As transcriptional effects were rather moderate compared to the strong phenotype, we wondered whether chm might also have a transcription-independent effect on the proteome. We therefore measured proteomic changes upon *chm* knockdown. We identified approximately 4,000 proteins, of which 158 proteins showed

Figure 2. Transcriptome suggests a moderate inhibitory effect on metabolism and stress response in fed $chm^{MYST/+}$ flies.

A PCA plot of control (w^{1118}) and $chm^{MYST/+}$ male flies at fed condition ($N = 5$, unpaired).

B Heatmap showing Spearman's correlation of control (w^{1118}) and $chm^{MYST/+}$ male flies' transcriptome at fed condition.

C MA plot comparing the log2FoldChange and mean expression. Significant genes are highlighted in filled and unfilled red circles for FDR < 0.05 and $\Pi < 0.05$, respectively.

D Tree plot depicting the top 30 significant GO terms from GSEA of the fed transcriptome. Color of the circles indicates enrichment and the size indicates number of genes annotated with that pathway. GO terms were clustered based on semantic similarity and the terms that were represented the most within a cluster were mentioned.

E GSEA plot for response to starvation and response to nutrient-level GO terms.

Data information: All replicates are independent biological replicates.

© 2023 The Authors

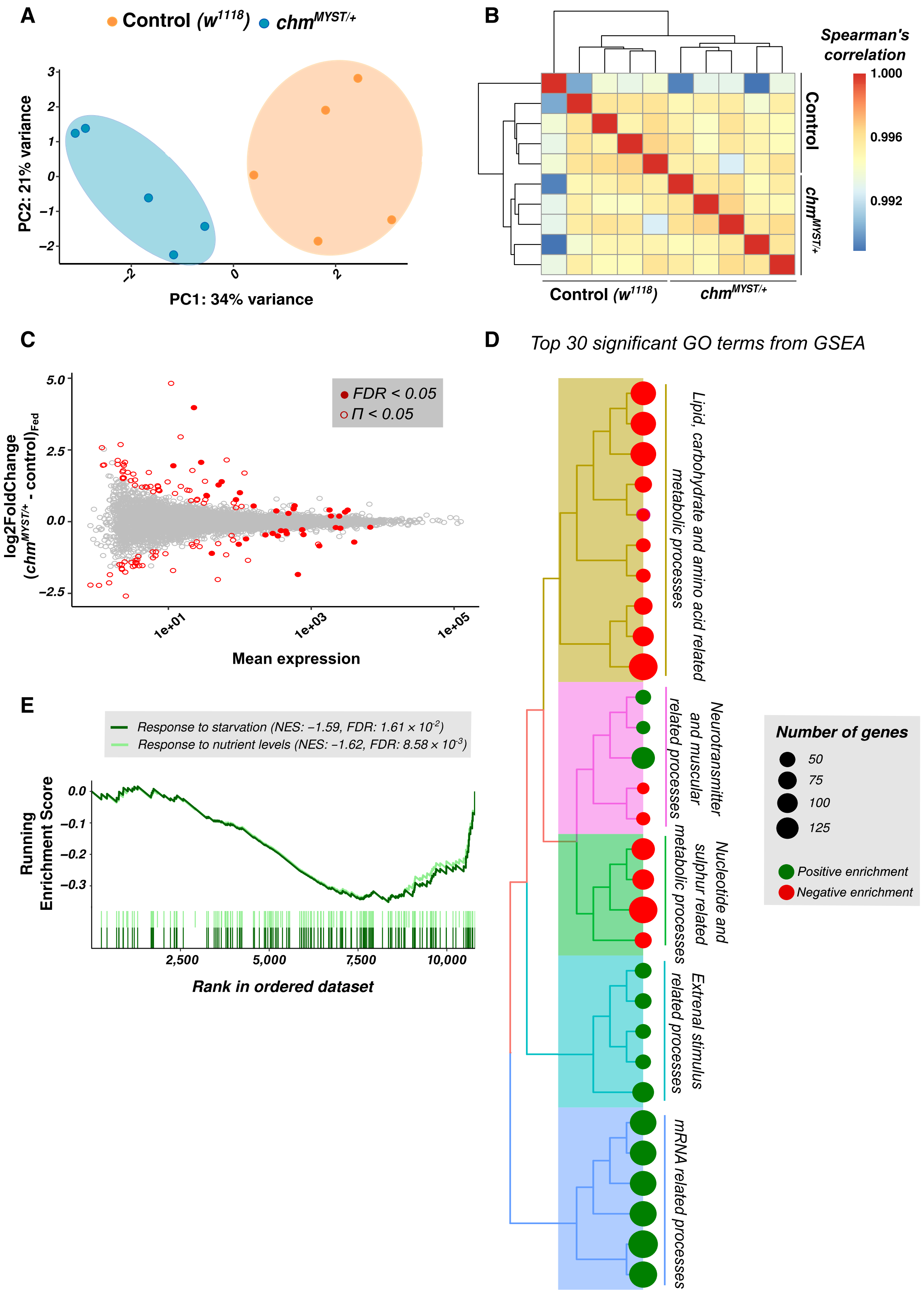

Figure 2.

significant differences (Figs 3A and EV2A; Dataset EV3). Similar to the transcriptional changes, GESA revealed a reduction in proteins involved in metabolic processes such as amino acid, small molecule, lipid, and carbohydrate metabolism upon loss of chm. Moreover, *chm* knockdown flies had higher levels of several immune response proteins (Fig 3B; Dataset EV4).

© 2023 The Authors

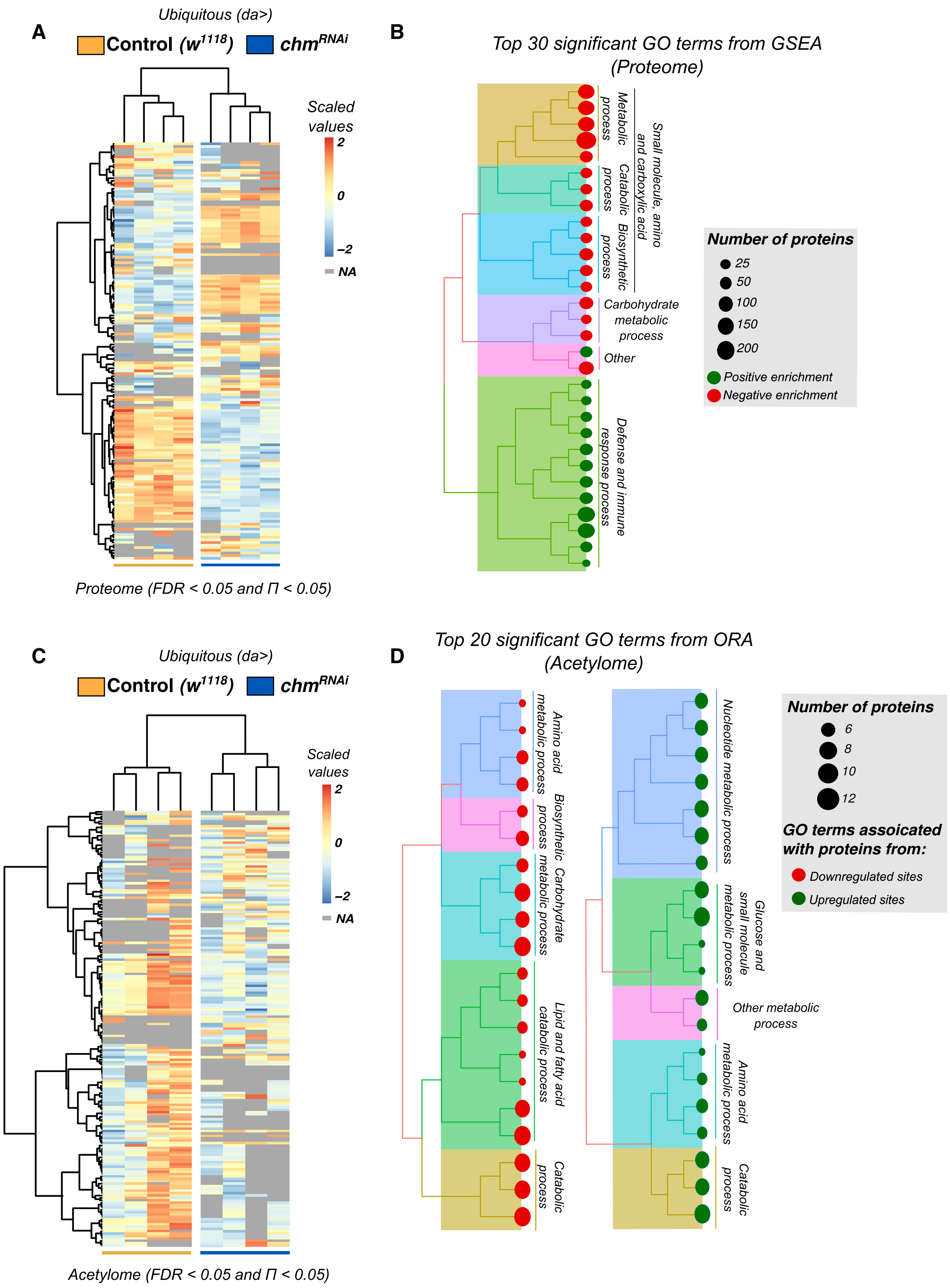
A
Ubiquitous (da>)
Control (w1118)
chmRNAi
Scaled values
2
0
−2
NA
Proteome (FDR < 0.05 and Π < 0.05)
B
Top 30 significant GO terms from GSEA (Proteome)
Metabolic process
Catabolic process
Biosynthetic process
Small molecule, amino and carboxylic acid
Carbohydrate metabolic process
Other
Defense and immune response process
Number of proteins
25
50
100
150
200
Positive enrichment
Negative enrichment
C
Ubiquitous (da>)
Control (w1118)
chmRNAi
Scaled values
2
0
−2
NA
Acetylome (FDR < 0.05 and Π < 0.05)
D
Top 20 significant GO terms from ORA (Acetylome)
Amino acid metabolic process
Biosynthetic process
Carbohydrate metabolic process
Lipid and fatty acid catabolic process
Catabolic process
Nucleotide metabolic process
Glucose and small molecule metabolic process
Other metabolic process
Amino acid metabolic process
Catabolic process
Number of proteins
6
8
10
12
GO terms assoicated with proteins from:
Downregulated sites
Upregulated sites

Figure 3.

Figure 3. Proteome and acetylome bolster the regulatory effect of chm on *Drosophila* metabolism.

A Heatmap showing all significant proteins (FDR < 0.05 and Π < 0.05) between control (w^{1118}) and chm^{RNAi}. Values in the heatmap are scaled raw intensity values. NAs are indicated in gray.

B Tree plot depicting the top 30 significant GO terms from GSEA of the fed proteome. Color of the circles indicates enrichment and the size indicates number of proteins annotated with that pathway. GO terms were clustered based on semantic similarity and the terms that were represented the most within a cluster were mentioned.

C Heatmap showing all significant sites (FDR < 0.05 and Π < 0.05) between control (w^{1118}) and chm^{RNAi}. Values in the heatmap are scaled raw intensity values. NAs are indicated in gray.

D Tree plot depicting the top 20 significant GO terms from ORA of the fed acetylome. Color of the circles indicates enrichment and the size indicates number of proteins annotated with that pathway. GO terms were clustered based on semantic similarity and the terms that were represented the most within a cluster were mentioned.

Data information: All replicates are independent biological and paired replicates (*N* = 4).

Subsequently, we wondered how chm affects the cellular acetylome being an acetyltransferase. Analysis of acetylome upon *chm* knockdown identified a total of 182 acetylation sites (of approx. 2,400) that were significantly altered (Figs 3C and EV2B; Dataset EV5). Similar to the effects observed for the transcriptome and the proteome, the differential acetylated proteins were frequently involved in metabolic processes of carbohydrate, lipid, and amino acids (Fig 3D; Dataset EV6), suggesting that loss of chm leads to misregulated acetylation of proteins involved in metabolism.

Overall, these results indicate a role of chm in regulating metabolism. As changes in metabolism may affect the organism's ability to respond to nutritional challenges, we wondered whether chm changes the flies' ability to cope with metabolic stress.

Chameau (chm) and its enzymatic activity are required for regulating nutritional stress response independent of its role in development

To test if chm is required to cope with starvation, we subjected flies compromised for *chm* expression to wet starvation. Consistent with the prediction from changes in transcriptome, proteome, and acetylome, we observed a strong decrease in survival during starvation in both male and female flies of chm^{RNAi} compared to their corresponding controls (Figs 4A and EV3A). This effect is independent of the RNAi line used (Fig EV3B, C and H). Furthermore, selective loss of chm in neurons and fat body also increased the flies' sensitivity to starvation (Fig 4B and C). Muscle-specific knockdown of *chm* in flies showed no differences compared to its control flies (Fig 4D) further supporting the hypothesis that chm exerts its function in both neurons and fat body (Fig EV3D).

Earlier studies have shown that chm is required for proper development (Aggarwal & Calvi, 2004; Hainaut *et al*, 2012; McConnell *et al*, 2012). We therefore wanted to assess if the observed sensitivity toward starvation is dependent on its role in developmental regulation. We therefore employed the GeneSwitch (Osterwalder *et al*, 2001) system to induce an adult-specific knockdown of *chm*. Flies that were not fed with RU486 (or ethanol fed; Fig 4E) and flies that were fed with RU486 for 2 days before starvation (Fig EV3E) showed no differences in starvation response. However, when fed with RU486 for 4 days before the start of starvation, chm^{RNAi} showed increased susceptibility to starvation as compared to the controls (Fig 4F). These data indicate that regulation of nutrient stress by chm is not coupled to its role in development. Finally, we also observed sensitivity to starvation in $chm^{MYST/+}$ male and female flies (Figs 4G and EV3F), suggesting that the resilience towards metabolic stress requires at least the partial role of chm's enzymatic activity (Fig EV3G). In order to be sure, we also tested the starvation response using a GFP-RNAi line. In contrast to the chm RNAi line, the GFP-RNAi showed an even higher survival than the w1118 male flies (Fig EV3H). As the GFP-RNAi was not backcrossed with w1118, while da-gal4, the other RNAi lines and the MYST/+ mutant flies were, we used w1118 as control in all our experiments.

Chm mutant flies show a dampened transcriptomic response upon starvation

To test whether chm modulates the expression of genes upon starvation, we analyzed the transcriptional response of control and $chm^{MYST/+}$ flies to wet starvation (Fig 5A). Consistent with a modulatory role of *chm* activity in response to starvation, PCA of the

Figure 4. Loss of chm and its activity increase the susceptibility to nutrient stress.

A Average survival curve of control (w^{1118}) and chm^{RNAi} male flies upon ubiquitous knockdown of chm with *da-Gal4* (*N* = 4, paired, log-rank test was performed for each biological replicate. All replicates had *P*-values < 0.0001).

B Average survival curve of control (w^{1118}) and chm^{RNAi} male flies upon fat body knockdown of chm with *r4-Gal4* (*N* = 4, paired, log-rank test was performed for each biological replicate. All replicates had *P*-values < 0.0001).

C Average survival curve of control (w^{1118}) and chm^{RNAi} male flies upon neuronal knockdown of chm with *elav-Gal4* (*N* = 4, paired, log-rank test was performed for each biological replicate. All replicates had *P*-values < 0.0001).

D Average survival curve of control (w^{1118}) and chm^{RNAi} male flies upon muscle knockdown of chm with *mef-Gal4* (*N* = 4, paired, log-rank test was performed for each biological replicate. All replicates had *P*-values < 0.0001).

E, F Average survival curve between (E) ethanol (*N* = 3, paired log-rank test was performed for each biological replicate. *P*-values ranged between 0.2 and 0.05 across replicates) and (F) RU486 (*N* = 4, paired, Log-rank test was performed for each biological replicate with all of them having a *P*-value < 0.05) and administered control (w^{1118}) and chm^{RNAi} male flies using *Act-GS-Gal4* for adult-specific chm knockdown.

G Average survival curve of control (w^{1118}) and $chm^{MYST/+}$ male flies (*N* = 5, paired log-rank test was performed for each biological replicate with all of them having a *P*-value < 0.0001).

Data information: All replicates are independent biological replicates and error bars indicate standard error of the mean (SEM). For survival curves, log-rank test was performed for each biological replicate. The displayed *P*-value is based on all biological replicates.
Source data are available online for this figure.

© 2023 The Authors

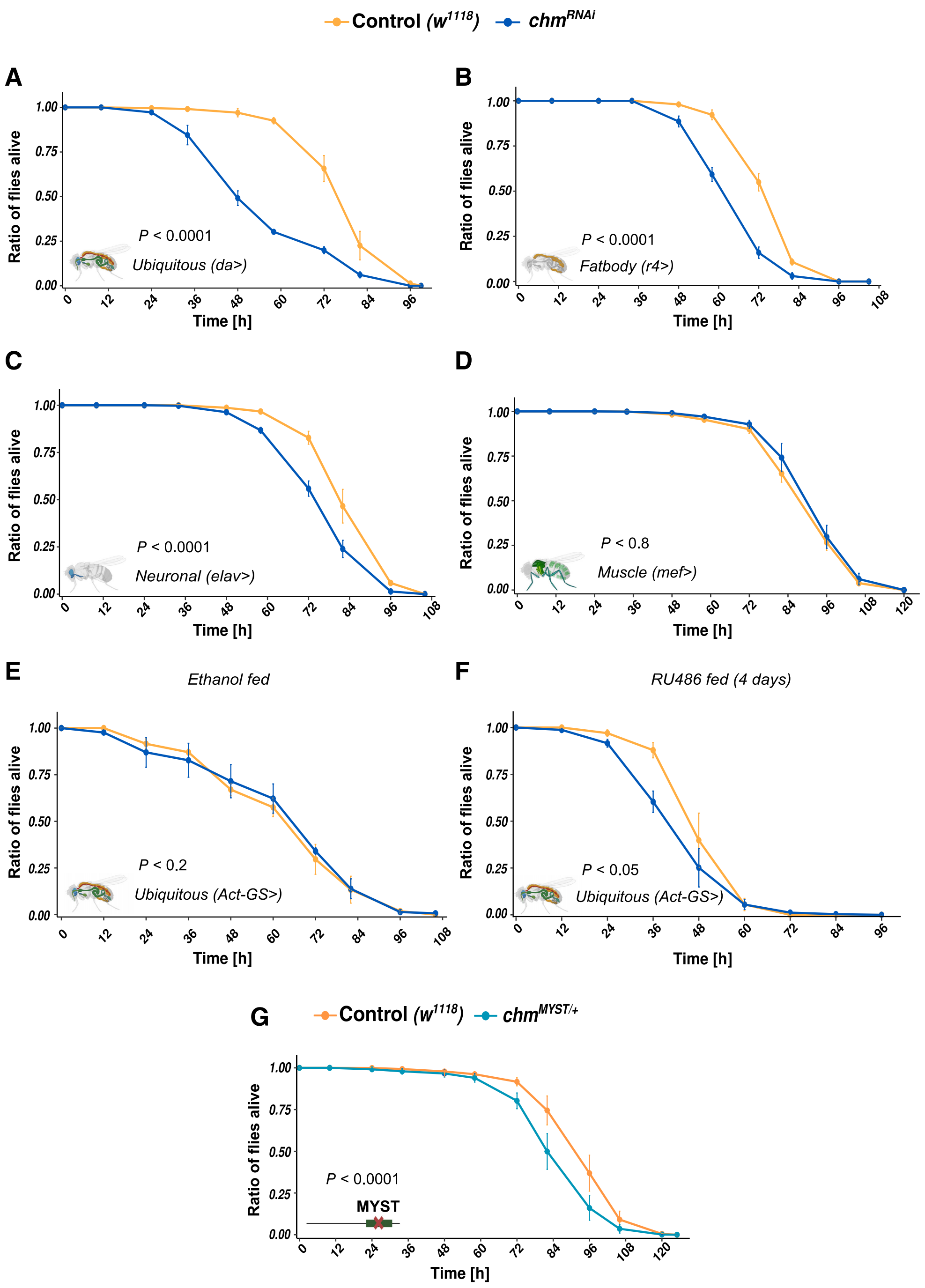

Figure 4.

transcriptome of fed and starved flies separated primarily based on its nutrient status (PC1) and only secondarily on their corresponding genotypes (PC2; Fig 5B). Furthermore, we noticed that *chm* mRNA levels were unaffected by starvation (Fig EV4A).

In accordance with this finding, we did not observe many differences in gene expression when comparing the transcriptional response to starvation between *chm*$^{MYST/+}$ and control flies (Figs 5B and EV5C; Dataset EV7 and EV8), such that a large overlap of 745 significant genes were shared between the genotypes upon starvation (Fig 5C). To investigate whether chm modulates the amplitude of transcriptional change, we performed GSEA for change in transcript levels between control and *chm*$^{MYST/+}$ flies in response to starvation. Although most GO terms were shared (Fig EV4C; Dataset EV9 and EV10), lack of chm activity clearly dampened the transcriptional response of genes involved in stress and starvation response and carbohydrate and lipid metabolism, upon starvation (Fig 5D–G). Interestingly, genes in autophagy were upregulated upon starvation as expected but they did not show genotype-specific differences (Fig EV4D). We, therefore, conclude that *chm*$^{MYST/+}$ flies respond to starvation but much less efficiently than wild-type flies. Overall,

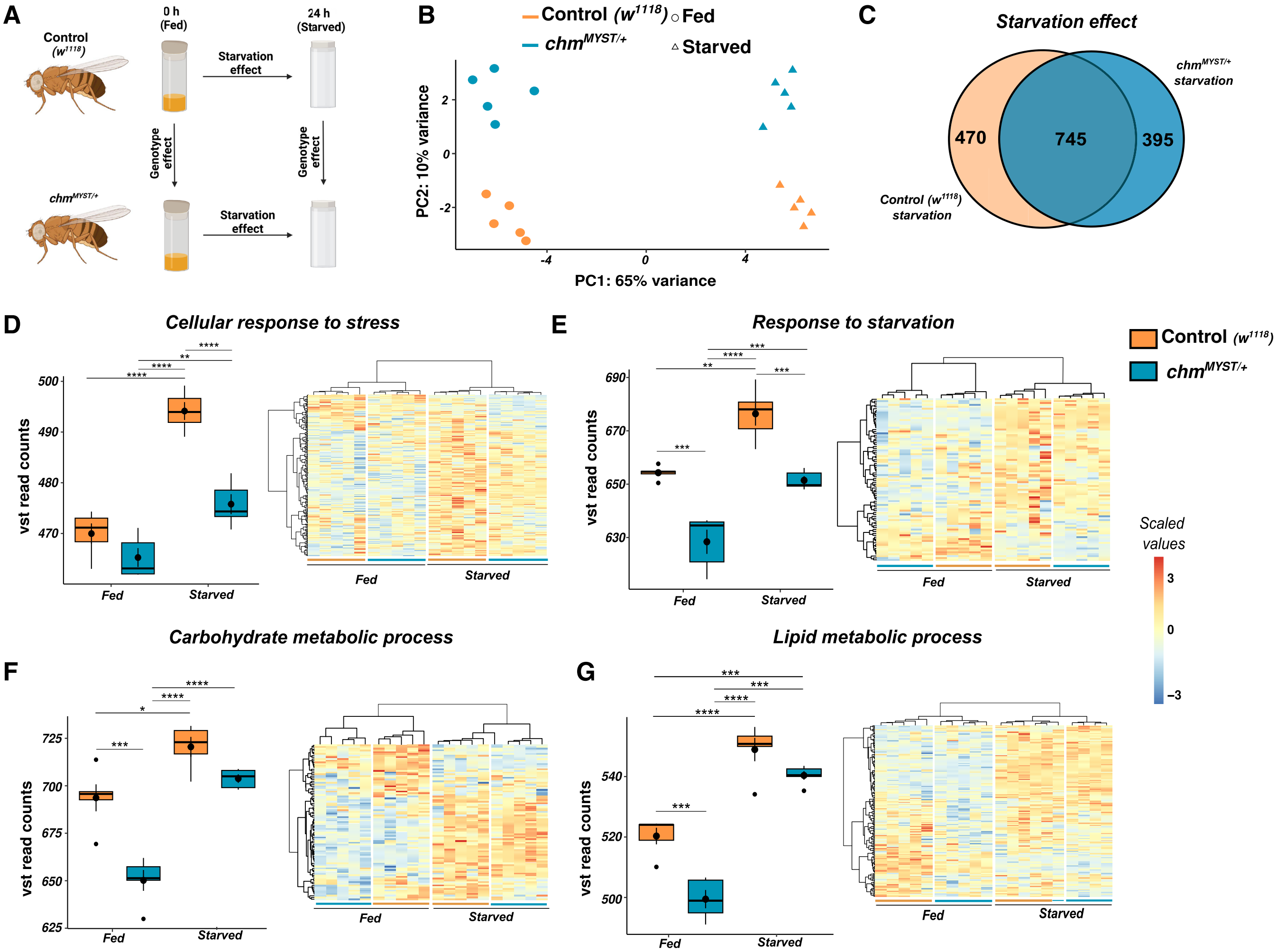

Figure 5. Mutant *chm* flies show a dampened transcriptomic profile upon starvation.

A Experimental design for transcriptomic analysis of fed and starved control (w^{1118}) and $chm^{MYST/+}$ flies.

B PCA of the transcriptome between fed and starved control (w^{1118}) and $chm^{MYST/+}$ flies ($N = 5$, unpaired). Colors indicate the genotype and shapes indicate the nutrient status.

C Venn diagram of significant genes (FDR < 0.05 and $\Pi < 0.05$) between control (w^{1118}) and $chm^{MYST/+}$ in response to starvation.

D–G Box plot (left) and heatmap (right) of annotated genes from GO terms of D) cellular response to stress (ANOVA *P*-value: 6.49×10^{-08}); E) Response to starvation (ANOVA *P*-value: 3.09×10^{-07}); F) Carbohydrate metabolic process (ANOVA *P*-value: 5.46×10^{-07}); G) Lipid metabolic process (ANOVA *P*-value: 7.88×10^{-09}); Box plot shows the average vst normalized read counts of all annotated genes within the GO term averaged over the replicates. Boxes indicate interquartile range with central band as the median and central filled dot as the average, and the whiskers indicate the maximum and minimum values across five biological replicates. Heatmap shows the scaled values of vst normalized read counts in each replicate for all conditions.

Data information: All replicates are independent biological replicates and error bars indicate standard error of the mean (SEM). Tukey test was performed and non-significant values are not shown (*$P < 0.05$, **$P < 0.01$, ***$P < 0.001$, ****$P < 0.0001$).
Source data are available online for this figure.

these data indicate that enzymatic activity of chm is required to mount a full transcriptional response when the organism is exposed to novel and stressful conditions, such as starvation.

Overexpression of chm improves weight and starvation susceptibility in *Drosophila*

We then wanted to address whether the metabolic phenotypes can be rescued by restoring the full activity of chm. For this, we used $chm^{MYST/+}$ flies carrying a *UAS-chm.myc* transgene. We used *da-Gal4* for strong and *arm-Gal4* for weak expression and measured the weight of flies expressing different levels of wild-type *chm*. Consistent with chm playing a role in storing metabolic energy, we noticed a gain of weight that was dependent on the overall expression levels (Figs 6A and EV5A). Both drivers also improved starvation resistance in a $chm^{MYST/+}$ background (Fig 6B and C) supporting the hypothesis that both phenotypes are caused by similar mechanisms. Furthermore, *da-Gal4*-driven expression resulted in a stronger increase in survival upon starvation as compared to *arm-Gal4*, further indicating significance of active chm in regulating

 © 2023 The Authors

stress response (Fig EV5B). In addition, we also performed rescue experiments using tissue-specific driver lines (*r4-Gal4* for fat body, *elav-Gal4* for neurons, and *mef-Gal4* for muscles). As expected, neuronal and fat body expression but not muscle-specific expression increased the survival consistently, validating the role of chm in neuronal and fat body regulation of starvation (Fig 6D–F). Furthermore, chm expression in fat body showed a stronger increase in survival as compared to neuronal tissue-specific expression, therefore bolstering the role of chm in fat body and metabolism (Fig EV5C).

In summary, these results validate chm as an important modulator of metabolism that could influence the organisms' phenotype under non-ideal conditions at multiple levels.

Discussion

Our current data show that the acetyltransferase chm is important for maintenance of physiology and starvation resilience in *Drosophila melanogaster*. We show that a knockdown of *chm* perturbs the physiology of the fly as they are thinner than control flies. This phenotype is also observed in flies that have a reduced chm catalytic activity, suggesting a key role of chm's acetyltransferase activity in this process. This is not only due to chm's ability to acetylate H4K12ac but also due to its function in regulating protein levels and its ability to acetylate other non-histone substrates. In fact, the latter might be more relevant for chm's role in modulating the response to starvation as we did not observe a strong effect of starvation on H4 acetylation (Appendix Fig S1A and B).

The fact that selective loss of chm in neurons and fat body is sufficient for the phenotype suggests that behavioral as well as metabolic changes in these flies could contribute to their thin body and response to starvation. To investigate whether the weight loss is primarily due to a behavioral change, we tested the feeding behavior of the flies ubiquitously expressing a *chm* RNAi and in a $chm^{MYST/+}$ background. While we observed reduced feeding in chm^{RNAi} flies upon ubiquitous knockdown, MYST/+ mutants showed no such

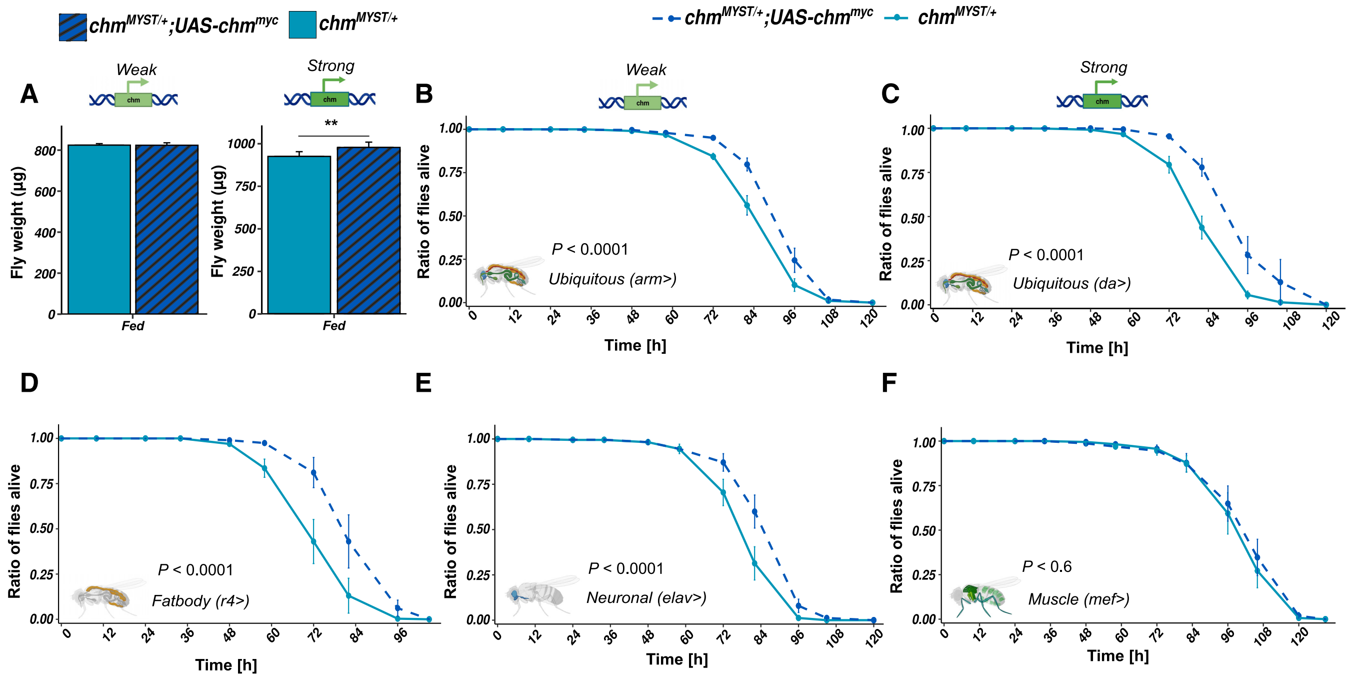

Figure 6. Overexpression of chm improves weight and survival.

A Weight of 8- to 9-day-old male $chm^{MYST/+}$ and $chm^{MYST/+}$;*UAS-chm*^{myc} flies upon weak (left, *N* = 5, paired) and strong (right, *N* = 4, paired, log-rank test was performed for each biological replicate. All replicates had *P*-values < 0.0001) chm overexpression with *arm-Gal4* and *da-Gal4*, respectively (paired *t*-test was performed).

B Average survival curve of $chm^{MYST/+}$ and $chm^{MYST/+}$;*UAS-chm*^{myc} male flies upon ubiquitous weak expression of *chm* with *arm-Gal4* (*N* = 4, paired, log-rank test was performed for each biological replicate. All replicates had *P*-values < 0.0001).

C Average survival curve of $chm^{MYST/+}$ and $chm^{MYST/+}$;*UAS-chm*^{myc} male flies upon ubiquitous strong expression of *chm* with *da-Gal4* (*N* = 3, paired, log-rank test was performed for each biological replicate. All replicates had *P*-values < 0.0001).

D Average survival curve of $chm^{MYST/+}$ and $chm^{MYST/+}$;*UAS-chm*^{myc} male flies upon fat body expression of *chm* with *r4-Gal4* (*N* = 4, paired, Log-rank test was performed for each biological replicate. All replicates had *P*-values < 0.0001).

E Average survival curve of control $chm^{MYST/+}$ and $chm^{MYST/+}$;*UAS-chm*^{myc} male flies upon neuronal expression of *chm* with *elav-Gal4* (*N* = 5, paired, Log-rank test was performed for each biological replicate. All replicates had *P*-values < 0.0001).

F Average survival curve of $chm^{MYST/+}$ and $chm^{MYST/+}$;*UAS-chm*^{myc} male flies upon muscle expression of *chm* with *mef-Gal4* (*N* = 5, paired, log-rank test was performed for each biological replicate. *P*-values ranged between 0.6 and 0.0001 across replicates).

Data information: All are independent biological replicates and error bars indicate standard error of the mean (SEM). Paired *t*-test was performed for the fly weight data and non-significant values are not shown (**P* < 0.05, ***P* < 0.01, ****P* < 0.001, *****P* < 0.0001). For survival curves, log-rank test was performed for each biological replicate. The displayed *P*-value is based on all biological replicates.
Source data are available online for this figure.

© 2023 The Authors

differences. However, both cases showed a strong starvation susceptibility suggesting that feeding behavior does not explain the reduced starvation resilience. Moreover, previous studies from our lab have shown that *chm*$^{MYST/+}$ mutants are more active than its corresponding control of same age (Peleg *et al*, 2016a). In addition, the fact that effect of a *chm* loss on the response to starvation can also be observed when chm is removed in adults using the GeneSwitch system. The latter experiments also show that the effect is not due to chm's role in fly development (Aggarwal & Calvi, 2004; Hainaut *et al*, 2012; McConnell *et al*, 2012).

The increased survival under normal conditions and the higher sensitivity toward starvation seem contradictory at first glance. However, they are very likely caused by the same regulatory mechanism. The leaner body mass of flies lacking chm is similar to what is seen during caloric restriction. While this state can result in lifespan extension (Peleg *et al*, 2016a,b) as long as sufficient nutrients are available, the system does not respond appropriately when exposed to less favorable conditions (Appendix Fig S2). A similar inability to mount a proper response to environmental challenges has recently been shown to lead to a selective growth advantage of epigenetically disrupted cancer cells (Loukas *et al*, 2023).

The phenotypic inertia is a consequence of the dampened response in flies lacking chm activity. This resilience to changing nutrient supplies is likely mediated on several levels as we observe an effect of a reduced chm activity on the transcriptome, the proteome, and the acetylome. While we are unable to identify a single pathway that is responsible for this effect, the affected transcripts and proteins show a clear enrichment for GO terms associated with many metabolic processes. Such interplay between an epigenetic enzyme and metabolism, especially upon starvation, is not uncommon and has been shown previously in *Drosophila* (Nakajima *et al*, 2016; Wang *et al*, 2020; Charidemou *et al*, 2022).

Finally, to our knowledge, this is probably the first evidence of an enzyme having opposing effects on longevity and nutrient stress. We speculate that while having less chm increases longevity, the requirement to survive under stress in a novel environment outweighs the benefit of a long and healthy lifespan. Our study therefore provides an explanation for the evolutionary conservation of chm in *Drosophila melanogaster*.

Table 1. Composition of fly food.

Components	Amount
Agarose	130 g
Corn meal	1,300 g
Soy flour	150 g
Yeast	300 g
Maltase	650 g
Molasses	1,300 g
Methyl 4-Hydroxybenzoate (Nipagin)	415 ml Ethanol (10%)
Acid mix	295 ml
Water	Up to 16 l

Table 2. Details of fly lines used in this experiment.

Fly lines	Source	ID
w^{1118}	Gift from Carla Margulies, University of Munich (LMU)	–
+;cyo[a]	Gift from Carla Margulies, University of Munich (LMU)	–
chm^{JF02348}[a]	Bloomington	#27027
chm^{v5684}[a]	VDRC	#5684
chm^{14}[a]	Gift from Yacine Graba, IBDM (France)	–
chm^{14}*/cyoGFP*	Gift from Yacine Graba, IBDM (France)	–
chm^{14}*/cyoGFP;UAS-chm*^{myc}	Gift from Yacine Graba, IBDM (France)	–
arm-Gal4[a]	Bloomington	#1560
elav-Gal4[a]	Gift from Carla Margulies, University of Munich (LMU)	–
da-Gal4[a]	Gift from Miura Masayiki, University of Tokyo	–
mef-Gal4[a]	Gift from Carla Margulies, University of Munich (LMU)	–
Act5C.GAL4.Switch	Bloomington	#9431
GFP^{RNAi}	Bloomington	#41556

[a]Flies have been backcrossed to w^{1118} for at least seven generations.

Materials and Methods

Fly maintenance

Fly lines were maintained in an incubator (Panasonic, MLR-235H-PE) at 25°C with a 12 h/12 h light–dark cycle at 60% relative humidity. The composition of the fly food is given in Table 1. Details of fly lines used in the study are given in Table 2.

Starvation assay

As age, sex, temperature, light–dark cycle, and humidity could affect starvation response and resistance (Jang & Lee, 2015, 2018; Chauhan *et al*, 2021), we kept all these constant by using flies of age 8–9 days old developed and starved at 23°C with 60% relative humidity and 12 h light/12 h dark cycle. Approx. 50–60 flies were transferred to an empty fly bottle containing a tissue with 4 ml of water. Total number of flies for each experiment ranged from 100 to 250 for each genotype/condition tested. Readings were taken until flies were dead in all genotypes with dead flies counted every 10–12 h.

For GeneSwitch, 7- to 10-day-old males from the cross of *Act5C-GeneSwitch* (males) and *w*^{1118} or *w*^{1118}*;UAS-chm*^{RNAi} (females) were used. RU486 (mifepristone; Sigma-Aldrich) was administrated with food for 4–5 days prior to the food deprivation, and then with water during the deprivation. RU486 was dissolved in ethanol (10 mg/ml) and mixed with melted food or water in a final concentration of 0.2 mM. The same amount of ethanol was added to the food or water for the control groups. Males and females were separated 3 days prior to the food deprivation.

Longevity assay

Male flies of age 8–9 days old developed and aged at 23°C with 60% relative humidity and 12 h light/12 h dark cycle. Approx. 30 male

© 2023 The Authors

flies were used per bottle and the total number of flies ranged from 100 to 150 for each genotype tested. Food was changed and readings were taken once a week initially and once in 2–3 days when flies started dying. Readings were taken until all flies were dead in both genotypes.

Weight measurement

Flies of the fed conditions were transferred to a 2.0 ml tube and snap frozen in liquid nitrogen. Following this, weight of each empty tube was measured. Heads and bodies were obtained by passing through sieves. First sieve (width: 710 μm) separates the bodies from remaining and the second (width: 355 μm) separates heads from wings/legs (Analysensieb). A total of 20–30 heads and bodies were transferred to the corresponding 1.5 ml tube and the weight was measured again. Difference in weight between the two was considered as the corresponding weight of 20–30 flies from which weight per fly was calculated. Weights were measured using KERN ABJ 120-4NM weighing machine. Samples and all the components were kept in dry ice for the entire duration of the experiment.

Post-translational modification of histones

Sample preparation

Fly heads were obtained following the same procedure given in the *Body and head weight measurement* section. Approx. 300 μl of homogenization buffer (60 mM KCl, 15 mM NaCl, 4 mM $MgCl_2$, 15 mM HEPES [pH 7.5], 0.5% Triton-X-100, 0.5 mM DTT, 20 mM sodium butyrate, and 1 tablet protease inhibitor) was added to 30–50 fly heads and were homogenized extensively with an electrical stirrer (5 × 10 s ON and 15 s OFF). Following this, sonication was performed with Bioruptor® Pico for 3 × 10 s ON and 45 s OFF at 4°C. The obtained lysate was centrifuged at 20,817 *g* for 30 min. Obtained pellet was resuspended in 200 μl of 0.2 M H_2SO_4, vortexed heavily, and rotated overnight at 15 rpm and 4°C. Subsequently, overnight incubated lysate was centrifuged at 20,817 *g* (max. Speed) for 10 min at 4°C. Histone was precipitated by adding trichloroacetic acid (TCA; ThermoScientific, Cat. No 85183) to reach 26% final concentration. Tubes were mixed and incubated at 4°C for 2 h and spun at 20,817 *g* for 15 min. Pellets were washed thrice with ice-cold 100% acetone (VWR, Cat. No AA22928-K2; 5 min rotation at 4°C and 15 min of 20,817 *g* spin at 4°C between washes), dried for 15 min at room temperature, and resuspended in 20 μl of 1× Laemmli sample buffer for million cells and boiled at 95°C for 5 min. Samples were stored at −20°C until further use. The histones corresponding to 0.5 million cells were separated into 4–20% precast polyacrylamide gels (SERVA, Cat. No 43277.01). Gels were briefly stained with InstantBlue Coomassie Protein Stain (Abcam, Cat. No ab119211). For targeted mass-spectrometry analysis, histones bands were excised, washed once with MS-grade water (Sigma Aldrich, Cat. No 1153331000) and de-stained twice (or until transparent) by incubating 30 min at 37°C with 200 μl of 50% acetonitrile (ACN; Carl Roth, Cat. No 8825.2) in 50 mM ammonium bicarbonate (NH_4HCO_3; Carl Roth, Cat. No T871.1). Gel pieces were then washed twice with 200 μl MS grade and twice with 200 μl of 100% ACN to dehydrate them. Histones were in-gel acylated by first adding 20 μl of d6 acetic anhydride (Sigma-Aldrich, 175641-5G), followed by 40 μl of 100 mM NH_4HCO_3. After 5 min, 140 μl of 1 M NH_4HCO_3 was slowly added to the reaction. pH of the final solution should be around 7 (in cases where pH was acidic, few microliters of 1 M NH_4HCO_3 was added). Samples were incubated at 37°C for 45 min at 550 rpm. Following this, samples were washed five times with 200 μl of 100 mM NH_4HCO_3, four times with 200 μl of MS-grade water, and four times with 200 μl of 100% ACN. They were spun down briefly and all remaining ACN was removed. Gel pieces were rehydrated in 50 μl of trypsin solution (25 ng/ml trypsin in 100 mM NH_4HCO_3; Promega, Cat. No V5111) and incubated at 4°C for 30 min. After the addition of 150 μl of 50 mM NH_4HCO_3, histones were in-gel digested overnight at 37°C at 550 rpm. Peptides were sequentially extracted by incubating 10 min at room temperature with 150 μl of 50 mM NH_4HCO_3, twice with 150 μl of 50% ACN (in MS-grade water) 0.1% trifluoroacetic acid (TFA) and twice with 100 μl of 100% ACN. During each of the above washing steps, samples were sonicated for 3 min in a water bath followed by a brief spin down. Obtained peptides were dried using a centrifugal evaporator and stored at −20°C until resuspension in 30 μl of 0.1% TFA. For desalting, peptides were loaded in a C18 Stagetip (prewashed with 20 μl of methanol followed by 20 μl 80% ACN 0.1% TFA and equilibrated with 20 μl of 0.1% TFA), washed two times with 20 μl of 0.1% TFA, and peptides were eluted three times with 10 μl of 80% ACN 0.25% TFA. Flow through obtained from loading of peptides in C18 was further desalted with TopTip Carbon (glygen, Cat, No TT1CAR.96) by loading the flow through thrice (prewashed thrice with 30 μl of 100% ACN followed by equilibration thrice with 30 μl of 0.1% TFA), washed five times with 30 μl of 0.1% TFA, and eluted thrice with 15 μl of 70% ACN and 0.1% TFA. Eluted peptides from both desalting steps were combined and evaporated in a centrifugal evaporator, resuspended in 15–17 μl of 0.1% TFA, and stored at −20°C until mass spectrometry analysis.

Targeted mass spectrometry

Desalted histone peptides in 0.1% TFA were injected in an RSLCnano System (Thermo Fisher Scientific) and separated in a 15 cm analytical column (75 μm ID home packed with ReproSil-Pur C18-AQ 2.4 μm from Dr. Maisch) with a 50 min gradient from 4 to 40% ACN in 0.1% formic acid at 300 nl/min flowrate. The effluent from the HPLC was electrosprayed into Q Exactive HF mass spectrometer (Thermo Fisher Scientific). The MS instrument was programmed to target several ions except for the MS3 fragmentation (22). Survey full-scan MS spectra (from m/z 270 to 730) were acquired with resolution $R = 60{,}000$ at m/z 400 (AGC target of 3 × 106). Targeted ions were isolated with an isolation window of 0.7 m/z to a target value of 2 × 105 and fragmented at 27% normalized collision energy. Typical mass spectrometric conditions were as follows: spray voltage, 1.5 kV; no sheath and auxiliary gas flow; and heated capillary temperature, 250°C.

Data analysis

Raw data from mass spectrometry were analyzed using Skyline (Pino *et al*, 2020) v21.1. Peak integration was performed for H3 and H4 peptides for each of its corresponding modifications. Relative levels of each PTM were calculated from the obtained intensities using R environment based on the formula given in Tiwari *et al*, 2020.

© 2023 The Authors

Western blot

For protein extraction, 30–50 fly heads were homogenized in RIPA buffer using a mechanical homogenizer, followed by sonication with Bioruptor® Pico (Diagenode, B01060010) for 4× 10 s ON, 30 s OFF. This was followed by centrifuging at maximum speed for 30 min and at 4°C. The obtained supernatant was transferred to a new tube and Laemelli sample buffer was added. Samples were then boiled for 5 min at 95°C.

For Western blot, samples were then loaded onto a 4–20% precast gel (SERVA, 43277.01) and run at 90 V for 30 min followed by 160 V for 2 h (SDS running buffer (10×): Tris 30.2 g, glycine 142 g, and SDS 10 g, make it to 1 l with double-distilled water). Wet transfer was performed for 2 h and 30 min at 175 V and 4°C (transfer buffer [10×]: Tris 30.3 g and glycine 144 g, make it to 1 l with double-distilled water; for 1×:10× transfer buffer 100 ml, methanol 150 ml, and 20% SDS 1 ml, make it to 1 l with double-distilled water). Following this, blocking was performed by incubating the membrane with 5% milk in PBS for 60–90 min at room temperature. Primary antibody incubation was performed at 4°C overnight. PBS-T (0.1%) washes were then carried out for 3 × 5 min time interval and secondary antibody incubation was performed at room temperature for 60 min. Following this, PBS-T (0.1%) washes were carried out for 3 × 5 min time interval and membrane was developed by chemiluminescence method using ECL detection kit (Bio-Rad, Clarity™ Western ECL Substrate, 170-5061), and ImageLab™ (v6.0) was used for the analysis. Information about the antibodies used is given in Table 3.

Quantitative reverse transcriptase PCR

RNA extraction

Thirty heads were homogenized with an electrical stirrer with 500 µl of Trizol (Thermo Fisher; cat. no. 15596026). Chloroform was added at the ratio of 1:5 with Trizol and the solutions were mixed for 15 s by inverting the tubes. After 5 min incubation at room temperature, samples were centrifuged at 12,000 *g* for 15 min. Aqueous phase was transferred to a new tube from the centrifuged sample to which isopropanol was added at 1:1 ratio of the obtained aqueous phase, vortexed briefly, and incubated for 10 min at room temperature. They were then centrifuged for 10 min at 12,000 *g*. Supernatant was discarded and obtained pellet was washed with 750 µl of 80% ethanol. After brief vortexing, samples were centrifuged at 8,000 *g* for 5 min. Obtained supernatant was discarded and pellet was air-dried for 5 min inside the hood and resuspended in RNase-free water. RNA concentration and A260/280 ratio were measured with NanoDrop.

Table 3. List of antibodies used

Antibody	Raised	Dilution	Company/Catalog number
chm 3G3	Mouse	1:1	Homemade
Lamin	Mouse	1:1,000	Homemade
H3	Rabbit	1:5,000	Abcam (ab18521)
Anti-mouse	Sheep	1:5,000	RRID: AB772210
Anti-rabbit	Donkey	1:5,000	RRID: AB772206

DNase treatment, cDNA synthesis, and qRT–PCR

DNase treatment (Roche, DNase I recombinant, RNase-free from bovine pancreas, Cat. No. 04716728001) was performed with 1 µg of RNA as starting material following manufacturer's instructions. cDNA synthesis was performed using SuperScript III First Strand Synthesis System (Invitrogen, cat. No: 18080051, Random hexamer priming) using DNase-treated RNA. Each reaction was set up with/without Superscript III reverse transcriptase. Obtained cDNA was treated with RNaseH to remove the RNA–DNA duplex and diluted 1:5 with RNase-free water. Diluted cDNA was used for qPCR reaction with Fast SYBR™ Green Master Mix (Thermo Fisher Scientific, cat no: 4385612) following manufacturer's instructions and ran on a Lightcycler 480 II (Roche) instrument. Primer efficiency was calculated using serial dilutions and the corresponding melt curves were also assessed. Sequences of qPCR primers used are given in Table 4.

RNA sequencing

Library preparation

One microgram of RNA, obtained from fly heads, was used for library preparation. Both total and mRNA quality was assessed on a 2100 Bioanalyzer (Agilent Technologies, Cat. No G2939BA) using RNA pico assay kit (Agilent RNA 6000 Pico Kit, cat. No: 5067-1513) using manufacturer's protocol. rRNA depletion was performed using NEBNext rRNA Depletion Kit (Human/Mouse/Rat; NEB #E6310), and library preparation for RNA sequencing was performed using NEBNext Ultra II Directional RNA Library Prep Kit for Illumina (NEB #E7760) following manufacturer's protocol. Libraries were sequenced on an Illumina HiSeq 1500 instrument at the Laboratory of Functional Genomic Analysis (LAFUGA, Gene Center Munich, LMU).

Data analysis

A total of 50 bp paired-end reads were aligned to the *D. melanogaster* reference genome (release 6) using STAR aligner (version 2.5.3a) with providing GTF annotation (dmel-all-r6.17.gtf). Reads with multiple alignments were filtered by setting outFilterMultimapNmax parameter to 1. Reads were counted per gene with parameter –quantMode GeneCounts. BAM files were converted to normalized bedgraph coverages using genomeCoverageBed command (bedtools version 2.27.1) with scale parameter set to divide by the total number of reads and multiplied by a million. Bedgraph files were converted to tdf files (igvtools version 2.3.98) to visualize in the IGV browser.

Count tables (read counts per gene) were read into R environment and low-count genes were filtered out (at least three read per gene in 10% of the samples analyzed together). Differential expression analysis was performed by DESeq2 (Love *et al*, 2014) package (version 1.24) by adding replicate information as batch variable. Samples that were directly compared to each other were fitted in the same DESeq2 (Love *et al*, 2014) model. Log2FoldChange estimates and adjusted *P*-values were obtained by the results function (DESeq2) and an FDR cutoff < 0.05 was applied. In addition, the

Table 4. List of primer pairs used for qPCR.

Target gene	Forward primer (5′-3′)	Reverse primer (5′-3′)
Actin-5c	CAGAGCAAGCGTGGTATCCT	GTGTGGTGCCAGATCTTCT
chm-Exon8	CAATATCCAGCCGAGCTCAT	AGCCAAGAATTCGTCATCGT

© 2023 The Authors

less stringent Π-value that includes both statistical and biological information was used. Π-value takes into consideration log2FoldChange and *P*-value to obtain values between 0 and 1 (Xiao *et al*, 2014; Hostrup *et al*, 2022). For principal component analysis (PCA), batch effect was corrected by the remove batch effects function from limma (Ritchie *et al*, 2014; package version 3.52.0) on the normalized read counts.

Gene set enrichment analysis was performed on the obtained results from different conditions/comparisons using the gseGO function from clusterProfiler (Yu *et al*, 2012; package version 3.12.0) by ranking the genes based on *t*-statistic value without any $\log_2$FoldChange or *P*-adjusted cut-off. GSEA plots with selected GO terms were also generated with R environment with these selected GO terms having FDR < 0.05 cut-off.

Proteome and acetylome

Protein extraction

Approximately 1,000 male fly heads were collected and homogenized in an electrical homogenizer with 250 µl of lysis buffer (20 mM HEPES pH 8.0, 9 M urea, 1 mM sodium orthovanadate, 2.5 mM sodium pyrophosphate, 1 mM β-glycerophosphate, 1 mM Na-butyrate, and 60 µM sirtinol). After homogenization, sample volume was made up to 700 µl with lysis buffer. The obtained homogenate was rotated for 30 min (8 revolutions per min) at 4°C. Samples were then sonicated in a Bioruptor for six cycles (10 s ON, 45 s OFF) and again rotated for 10 min (8 revolutions per min) at 4°C. Centrifugation was performed at 14,000 *g* and 4°C for 20 min and the supernatant was transferred to another Eppendorf. This was followed by another centrifugation to remove the debris completely. The final supernatant was then transferred to a 15 ml falcon and the volume was made up to 2 ml with lysis buffer.

Acetylome sample preparation

Sample preparation for acetylome was performed with PTMScan® Acetyl-Lysine Motif [Ac-K] kit (Cell Signaling, 13416), and manufacturer's protocol was followed with minor modifications. To the obtain protein extract, 1/278th volume of 1.25 M DTT was added and incubated at room temperature for 60 min, followed by 15 min incubation in dark with 1/10th volume of iodoacetamide (Merck, 8.04744.0025). The supernatant was then diluted with 20 mM HEPES pH 8.0 and incubated (with mixing) with 1 mg/ml trypsin (Sigma-Aldrich, 175641-5G) at room temperature overnight. Trypsin was added at 1:100 ratio with total initial protein amount. Following overnight incubation, the digestion was confirmed with SDS–PAGE and 1/20 volume of 20% TFA was added to the digested peptide solution and incubated for 15 min on ice. The lysate was then centrifuged at 1,780 *g* for 15 min at room temperature to remove any precipitate.

For peptide purification, Sep-Pak® Light C18 cartridges filter column (Waters, WAT023501) was connected to a 10 cc syringe and the column was prewet with 5 ml 100% ACN, followed by sequential washes with 1, 3, and 6 ml of 0.1% TFA. The acidified digest was then loaded onto the column (without vacuum), followed by further washes of 1, 5, and 6 ml with 0.1% TFA, and then with 2 ml of wash buffer (0.1% TFA and 5% ACN). Elution of the peptide was carried out by washing the column with 0.1% TFA and 40% ACN. Eluted peptide was then frozen overnight at −80°C and lyophilized for at least 48 h.

Lyophilized peptide was centrifuged at 2,000 *g* for 5 min at room temperature, resuspended with 1.4 ml of the IAP buffer (provided by the manufacturer), and centrifuged again at 10,000 *g* at 4°C for 5 min. Resuspended samples were then quantified by BCA to make sure equal amount of peptides was used as input for proteome and acetylome. Ten percent of the cleared solution was taken as input for proteome. Lysine antibody beads (provided by the manufacturer) were washed with 1 ml of 1× PBS, centrifuged at 2,000 *g*, and resuspended with 40 µl of 1× PBS. To the cleared solution, half the volume of antibody bead slurry was used and incubated in a rotator for 2 h at 4°C. Incubated samples were then centrifuged at 2,000 *g* for 30 s, and supernatant was transferred to a new vial for further use. Beads were then washed twice with 1 ml of IAP buffer, mixed by inverting, centrifuged at 2,000 *g* for 30 s, and followed by similar washes, thrice with 1 ml chilled HPLC water. Peptides bound to the beads were eluted twice by adding 55 µl of 0.15% TFA, vortexing, incubating for 10 min at room temperature, and centrifuging for 30 s at 2,000 *g*.

Desalting of the eluted peptide was performed with AttractSPE® Disks Tips C18 column (affinisep, Tips-C18.T2.200.96). The column was first equilibrated with 50 µl 0.1% TFA twice. Input proteome and IP sample was then added to the C18, followed by washes with 0.1% TFA twice. Peptides were eluted with 10 µl of 0.1% TFA and 40% ACN, dried with vacuum concentrator, and resuspended with 15 µl of 0.1% TFA for mass spectrometry analysis.

Mass spectrometry

Samples were evaporated to dryness, resuspended in 15 µl of 0.1% formic acid solution, and injected in an Ultimate 3000 RSLCnano system (Thermo) separated in a 25 cm Aurora column (Ionopticks) with a 100 min gradient from 6 to 43% of 80% acetonitrile in 0.1% formic acid. The effluent from the HPLC was directly electrosprayed into an Orbitrap Exploris 480 (Thermo) operated in data-dependent mode to automatically switch between full-scan MS and MS/MS acquisition. Survey full-scan MS spectra (from *m/z* 350–1,200) were acquired with resolution *R* = 60,000 at *m/z* 400 (AGC target of 3 × 106). The 20 most intense peptide ions with charge states between 2 and 6 were sequentially isolated to a target value of 1×10^5, and fragmented at 30% normalized collision energy. Typical mass spectrometric conditions were as follows: spray voltage, 1.5 kV; no sheath and auxiliary gas flow; heated capillary temperature, 275°C; and intensity selection threshold, 3 × 105.

Data analysis

Raw flies from mass spectrometry were aligned using MaxQuant (Cox & Mann, 2008; v2.1.3.0) with *Drosophila* fasta from UniProt. Output files of proteome (proteinGroups.txt) and acetylome (Acetyl (K)sites.txt) from MaxQuant were analyzed in R environment. Proteins and acetylation sites were only used for further analysis when they were detected and quantified in at leat three out of four replicates of at least one condition. Following filtering, MinProb imputation algorithm with *q* = 0.01 was performed to impute the missing values, and limma (Ritchie *et al*, 2014)-based differential expression analysis was performed. Intensities of acetylome were not normalized to the proteome LFQ intensities and were used directly for

© 2023 The Authors

differential expression analysis. As with transcriptome, both *q*-value and Π-value (Xiao *et al*, 2014; Hostrup *et al*, 2022) were considered for significance with a cut-off of 0.05 in both cases. Gene set enrichment analysis was performed for the proteome from using the gseGO function clusterProfiler (Yu *et al*, 2012; package version 3.12.0) by ranking the genes based on *t*-statistic value without any log2FoldChange or *P*-adjusted cut-off (FDR < 0.05 cut-off for predicted GO terms). Overrepresentation analysis was performed without a background set for significant proteins in the acetylome by separating them based on log-fold change to obtain the up- and downregulated sites and their corresponding proteins (FDR < 0.05 cut-off for predicted GO terms). Corresponding GO plots were also generated with R environment. The script for data analysis was written based on DEP package (Zhang *et al*, 2018; v1.18.0) but was modified to be used for protein modification analysis. This will be provided on request as there is a possibility of publishing it as an R package in the future.

Plots and statistical analysis

All statistical analyses were performed in R (R Core Team, 2022; R: A language and environment for statistical computing and R Foundation for Statistical Computing, Vienna, Austria. URL https://www.R-project.org/) environment unless otherwise mentioned. Graphics in all figures were created using Biorender.com. Statistical tests were decided based on the experimental design and measurements and have been mentioned in each of the figure legends. Plots and graphs generated for all experiments were also generated in R environment unless otherwise mentioned.

Data availability

The datasets produced in this study are available in the following databases:
Transcriptomic data: Gene Expression Omnibus GSE211042 (http://www.ncbi.nlm.nih.gov/geo/query/acc.cgi?acc=GSE211042).
Histone PTM mass spectrometry data: ProteomeXchange Consortium via the PRIDE[20] partner repository PXD035947 (http://www.ebi.ac.uk/pride/archive/projects/PXD035947).
Acetylome and proteome mass spectrometry data: ProteomeXchange Consortium via the PRIDE[20] partner repository PXD042471 (http://www.ebi.ac.uk/pride/archive/projects/PXD042471).

Expanded View for this article is available online.

Acknowledgements
We would like to thank the members of the Imhof lab, Peleg lab, Tanimoto lab, Becker department, Andreas Ladurner, and Raffaele Teperino for their lively discussion, Catherine Regnard and Silke Krause for their help in RNA sequencing, Stefan Krebs and Helmut Blum from LAFUGA facility for sequencing, and Nicolas Gompel and Christa Schwarzlose for fly food and maintenance, respectively. We thank Tobias Straub, Tamas Schauer, and Wasim Aftab for their assistance in experimental design, statistics, and bioinformatic analysis. We acknowledge Carla Margulies, Yacine Graba, Masayuki Miura, and Fumiaki Obata for providing fly lines. In addition, we thank Markus Hohle from QBM for his constant support. AVV is supported by the QBM and SFB1309. Work in the AI lab was funded by grants from the DFG, grant numbers 213249687 (CRC1064) and 325871075 (CRC1309). The Peleg lab is supported by the FBN, DFG grant (458246576), and Longevity Impetus grant from Norn Group. Open Access funding enabled and organized by Projekt DEAL.

Author contributions
Anuroop Venkateswaran Venkatasubramani: Conceptualization; data curation; investigation; visualization; methodology; writing – original draft; writing – review and editing. **Toshiharu Ichinose:** Investigation; visualization; methodology. **Mai Kanno:** Methodology; writing – review and editing. **Ignasi Forne:** Formal analysis; visualization; methodology; writing – review and editing. **Hiromu Tanimoto:** Supervision; funding acquisition; methodology. **Shahaf Peleg:** Conceptualization; supervision; writing – review and editing. **Axel Imhof:** Conceptualization; resources; supervision; funding acquisition; writing – original draft; project administration; writing – review and editing.

Disclosure and competing interests statement
AI is a co-founder and shareholder of EpiQMAx. The other authors declare no competing interests.

References

Aggarwal BD, Calvi BR (2004) Chromatin regulates origin activity in *Drosophila* follicle cells. *Nature* 430: 372–376

Carthew RW (2021) Gene regulation and cellular metabolism: an essential partnership. *Trends Genet* 37: 389–400

Charidemou E, Tsiarli MA, Theophanous A, Yilmaz V, Pitsouli C, Strati K, Griffin JL, Kirmizis A (2022) Histone acetyltransferase NAA40 modulates acetyl-CoA levels and lipid synthesis. *BMC Biol* 20: 22

Chauhan V, Anis A, Chauhan A (2021) Effects of starvation on the levels of triglycerides, diacylglycerol, and activity of lipase in male and female *Drosophila melanogaster*. *J Lipids* 2021: 5583114

Cox J, Mann M (2008) MaxQuant enables high peptide identification rates, individualized p.p.b.-range mass accuracies and proteome-wide protein quantification. *Nat Biotechnol* 26: 1367–1372

Feller C, Forne I, Imhof A, Becker PB (2015) Global and specific responses of the histone acetylome to systematic perturbation. *Mol Cell* 57: 559–571

Flatt T (2020) Life-history evolution and the genetics of fitness components in *Drosophila melanogaster*. *Genetics* 214: 3–48

Grienenberger A, Miotto B, Sagnier T, Cavalli G, Schramke V, Geli V, Mariol MC, Berenger H, Graba Y, Pradel J (2002) The MYST domain acetyltransferase chameau functions in epigenetic mechanisms of transcriptional repression. *Curr Biol* 12: 762–766

Hainaut M, Sagnier T, Berenger H, Pradel J, Graba Y, Miotto B (2012) The MYST-containing protein chameau is required for proper sensory organ specification during *Drosophila* thorax morphogenesis. *PLoS One* 7: e32882

Hostrup M, Lemminger AK, Stocks B, Gonzalez-Franquesa A, Larsen JK, Quesada JP, Thomassen M, Weinert BT, Bangsbo J, Deshmukh AS (2022) High-intensity interval training remodels the proteome and acetylome of human skeletal muscle. *Elife* 11: e69802

Hsieh W-C, Sutter BM, Ruess H, Barnes SD, Malladi VS, Tu BP (2022) Glucose starvation induces a switch in the histone acetylome for activation of gluconeogenic and fat metabolism genes. *Mol Cell* 82: 60–74.e5

Iizuka M, Stillman B (1999) Histone acetyltransferase HBO1 interacts with the ORC1 subunit of the human initiator protein. *J Biol Chem* 274: 23027–23034

Jang T, Lee KP (2015) The genetic basis for mating-induced sex differences in starvation resistance in *Drosophila melanogaster*. *J Insect Physiol* 82: 56–65

 © 2023 The Authors

Jang T, Lee KP (2018) Context-dependent effects of temperature on starvation resistance in *Drosophila melanogaster*: mechanisms and ecological implications. *J Insect Physiol* 110: 6–12

Jeninga EH, Schoonjans K, Auwerx J (2010) Reversible acetylation of PGC-1: connecting energy sensors and effectors to guarantee metabolic flexibility. *Oncogene* 29: 4617–4624

Jenkins VK, Larkin A, Thurmond J, Consortium TF (2022) *Drosophila*, methods and protocols. *Methods Mol Biol* 2540: 1–34

Katada S, Imhof A, Sassone-Corsi P (2012) Connecting threads: epigenetics and metabolism. *Cell* 148: 24–28

Levine MT, Begun DJ (2008) Evidence of spatially varying selection acting on four chromatin-remodeling loci in *Drosophila melanogaster*. *Genetics* 179: 475–485

Loukas I, Simeoni F, Milan M, Inglese P, Patel H, Goldstone R, East P, Strohbuecker S, Mitter R, Talsania B *et al* (2023) Selective advantage of epigenetically disrupted cancer cells via phenotypic inertia. *Cancer Cell* 41: 70–87.e14

Love MI, Huber W, Anders S (2014) Moderated estimation of fold change and dispersion for RNA-seq data with DESeq2. *Genome Biol* 15: 550

McConnell KH, Dixon M, Calvi BR (2012) The histone acetyltransferases CBP and chameau integrate developmental and DNA replication programs in *Drosophila* ovarian follicle cells. *Development* 139: 3880–3890

Miotto B, Sagnier T, Berenger H, Bohmann D, Pradel J, Graba Y (2006) Chameau HAT and DRpd3 HDAC function as antagonistic cofactors of JNK/AP-1-dependent transcription during *Drosophila* metamorphosis. *Genes Dev* 20: 101–112

Molaei M, Vandehoef C, Karpac J (2019) NF-κB shapes metabolic adaptation by attenuating foxo-mediated lipolysis in *Drosophila*. *Dev Cell* 49: 802–810.e6

Nakajima E, Shimaji K, Umegawachi T, Tomida S, Yoshida H, Yoshimoto N, Izawa S, Kimura H, Yamaguchi M (2016) The histone deacetylase gene Rpd3 is required for starvation stress resistance. *PLoS One* 11: e0167554

Osterwalder T, Yoon KS, White BH, Keshishian H (2001) A conditional tissue-specific transgene expression system using inducible GAL4. *Proc Natl Acad Sci USA* 98: 12596–12601

Peleg S, Feller C, Forne I, Schiller E, Sévin DC, Schauer T, Regnard C, Straub T, Prestel M, Klima C *et al* (2016a) Life span extension by targeting a link between metabolism and histone acetylation in *Drosophila*. *EMBO Rep* 17: 455–469

Peleg S, Feller C, Ladurner AG, Imhof A (2016b) The metabolic impact on histone acetylation and transcription in ageing. *Trends Biochem Sci* 41: 700–711

Pino LK, Searle BC, Bollinger JG, Nunn B, MacLean B, MacCoss MJ (2020) The skyline ecosystem: informatics for quantitative mass spectrometry proteomics. *Mass Spectrom Rev* 39: 229–244

R Core Team (2022) *R: a language and environment for statistical computing*, Vienna, Austria: R Foundation for Statistical Computing

Ritchie ME, Phipson B, Wu D, Hu Y, Law CW, Shi W, Smyth GK (2014) limma powers differential expression analyses for RNA-sequencing and microarray studies. *Nucleic Acids Res* 43: e47

Rodrigues CP, Chatterjee A, Wiese M, Stehle T, Szymanski W, Shvedunova M, Akhtar A (2021) Histone H4 lysine 16 acetylation controls central carbon metabolism and diet-induced obesity in mice. *Nat Commun* 12: 6212

Shi L, Tu BP (2015) Acetyl-CoA and the regulation of metabolism: mechanisms and consequences. *Curr Opin Cell Biol* 33: 125–131

Tiwari SK, Toshniwal AG, Mandal S, Mandal L (2020) Fatty acid β-oxidation is required for the differentiation of larval hematopoietic progenitors in *Drosophila*. *Elife* 9: e53247

Wang Y, Huang Y, Liu J, Zhang J, Xu M, You Z, Peng C, Gong Z, Liu W (2020) Acetyltransferase GCN5 regulates autophagy and lysosome biogenesis by targeting TFEB. *EMBO Rep* 21: e48335

Wang W, Zheng Y, Sun S, Li W, Song M, Ji Q, Wu Z, Liu Z, Fan Y, Liu F *et al* (2021) A genome-wide CRISPR-based screen identifies KAT7 as a driver of cellular senescence. *Sci Transl Med* 13: eabd2655

Wu Z, Wei D, Gao W, Xu Y, Hu Z, Ma Z, Gao C, Zhu X, Li Q (2015) TPO-Induced metabolic reprogramming drives liver metastasis of colorectal cancer CD110+ tumor-initiating cells. *Cell Stem Cell* 17: 47–59

Xiao Y, Hsiao T-H, Suresh U, Chen H-IH, Wu X, Wolf SE, Chen Y (2014) A novel significance score for gene selection and ranking. *Bioinformatics* 30: 801–807

Ye C, Tu BP (2018) Sink into the epigenome: histones as repositories that influence cellular metabolism. *Trends Endocrinol Metab* 29: 626–637

Yu G, Wang L-G, Han Y, He Q-Y (2012) clusterProfiler: an R package for comparing biological themes among gene clusters. *OMICS* 16: 284–287

Zaini MA, Müller C, de Jong TV, Ackermann T, Hartleben G, Kortman G, Gührs K-H, Fusetti F, Krämer OH, Guryev V *et al* (2018) A p300 and SIRT1 regulated acetylation switch of C/EBPα controls mitochondrial function. *Cell Rep* 22: 497–511

Zhang X, Smits AH, van Tilburg GB, Ovaa H, Huber W, Vermeulen M (2018) Proteome-wide identification of ubiquitin interactions using UbIA-MS. *Nat Protoc* 13: 530–550

Zinke I, Schütz CS, Katzenberger JD, Bauer M, Pankratz MJ (2002) Nutrient control of gene expression in *Drosophila*: microarray analysis of starvation and sugar-dependent response. *EMBO J* 21: 6162–6173

License: This is an open access article under the terms of the Creative Commons Attribution License, which permits use, distribution and reproduction in any medium, provided the original work is properly cited.

© 2023 The Authors

Expanded View Figures

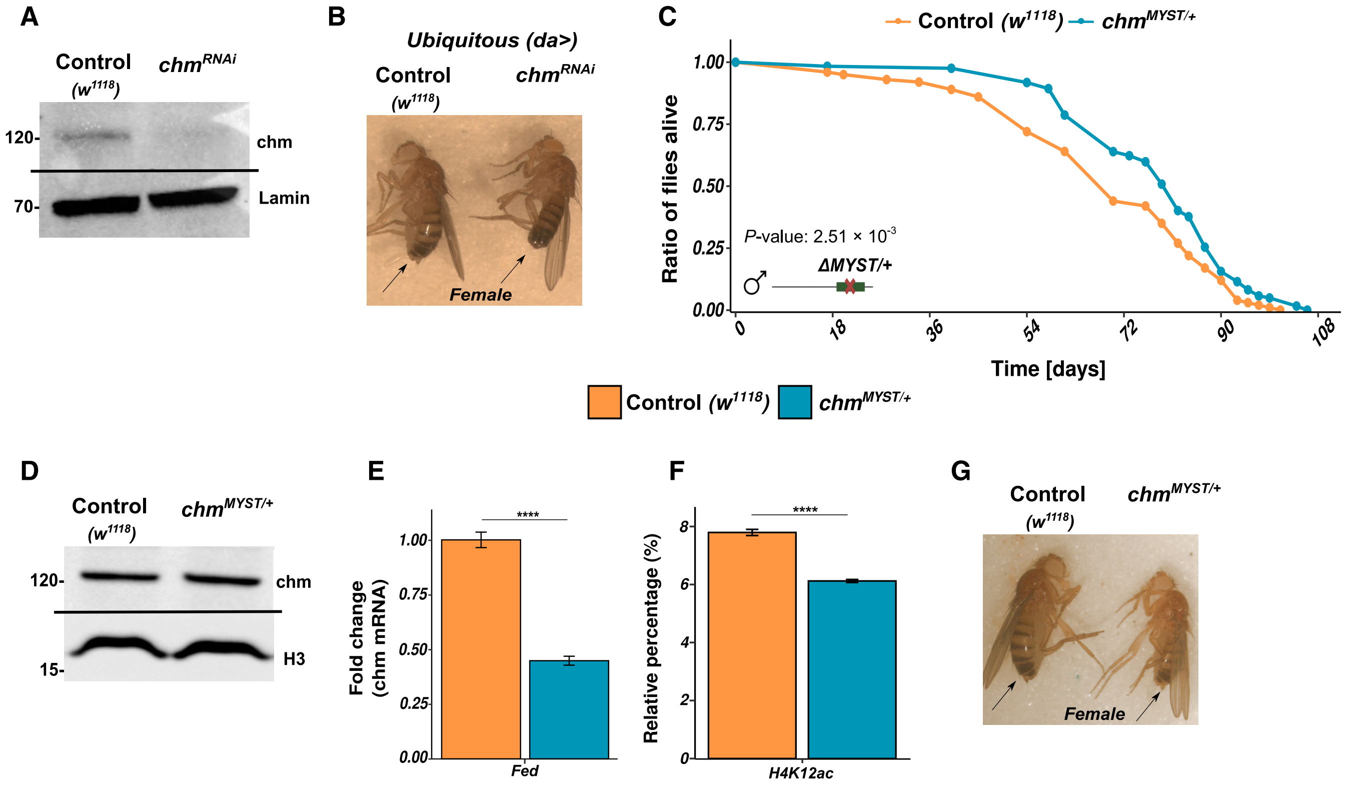

Figure EV1. Longevity in males is unaffected by temperature and female *chm* mutants also show changes in physiology.

A Western blot of control (w^{1118}) and chm^{RNAi}.

B Image of 8- to 9-day-old female control (w^{1118}, left) and chm^{RNAi} (right) flies upon ubiquitous *chm* knockdown.

C Survival curve showing increased lifespan of $chm^{MYST/+}$ flies at 23°C (N = 1).

D Western blot of control (w^{1118}) and $chm^{MYST/+}$.

E mRNA levels of *chm* in control (w^{1118}) and $chm^{MYST/+}$ flies (N = 4 (control) and 5 ($chm^{MYST/+}$), unpaired).

F Mass spectrometry quantified relative percentage of H4K12ac between control (w^{1118}) and $chm^{MYST/+}$ flies (N = 5, unpaired).

G Image of 8- to 9-day-old female control (w^{1118}, left) and $chm^{MYST/+}$ (right) flies.

Data information: All replicates are independent biological replicates and error bars indicate standard error of the mean (SEM). Unpaired t-test was performed and non-significant values are not shown (*$P < 0.05$, **$P < 0.01$, ***$P < 0.001$, ****$P < 0.0001$). For survival curves, log-rank test was performed.
Source data are available online for this figure.

© 2023 The Authors

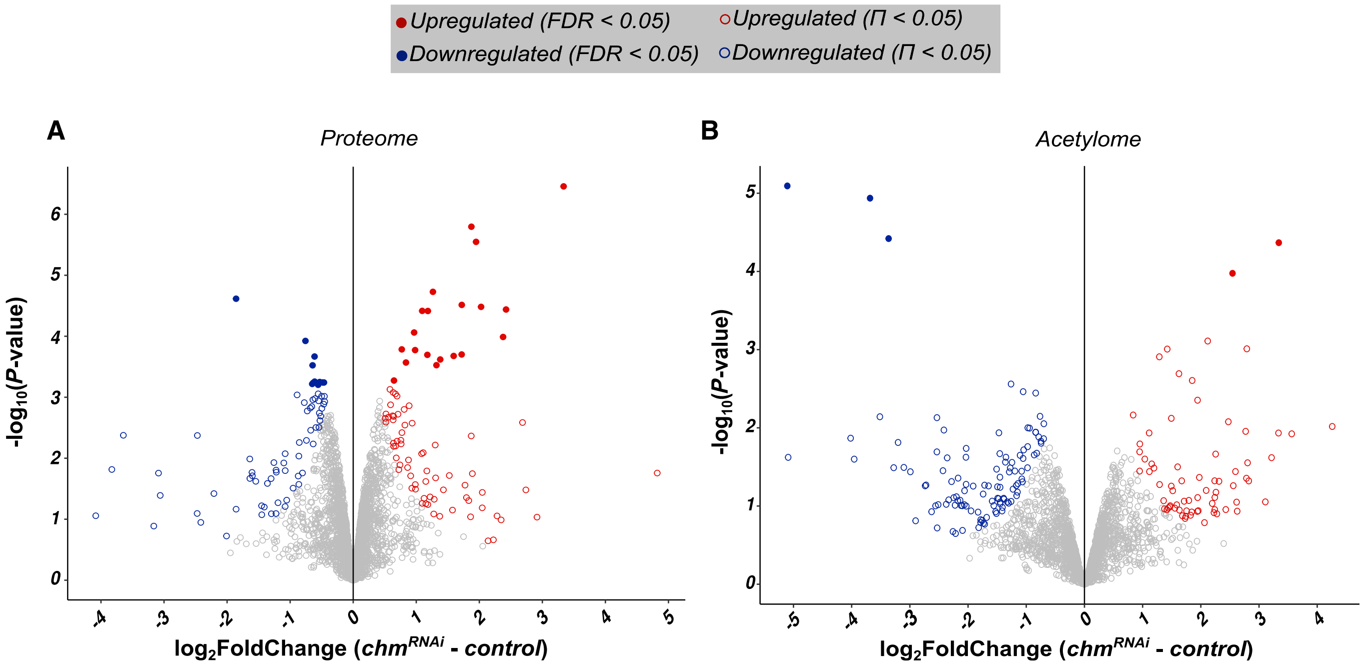

Figure EV2. Proteome and acetylome data show significant differential changes upon *chm* knockdown.

A, B Volcano plot showing the $\log_2$Foldchange (chm^{RNAi} - control) in x-axis and $\log_{10}P$-value in y-axis for A) proteome and B) acetylome (not normalized to the proteome). Filled circles indicate FDR < 0.05 and unfilled circles indicate $\Pi < 0.05$. Color of the circles indicates the nature of differential regulation.

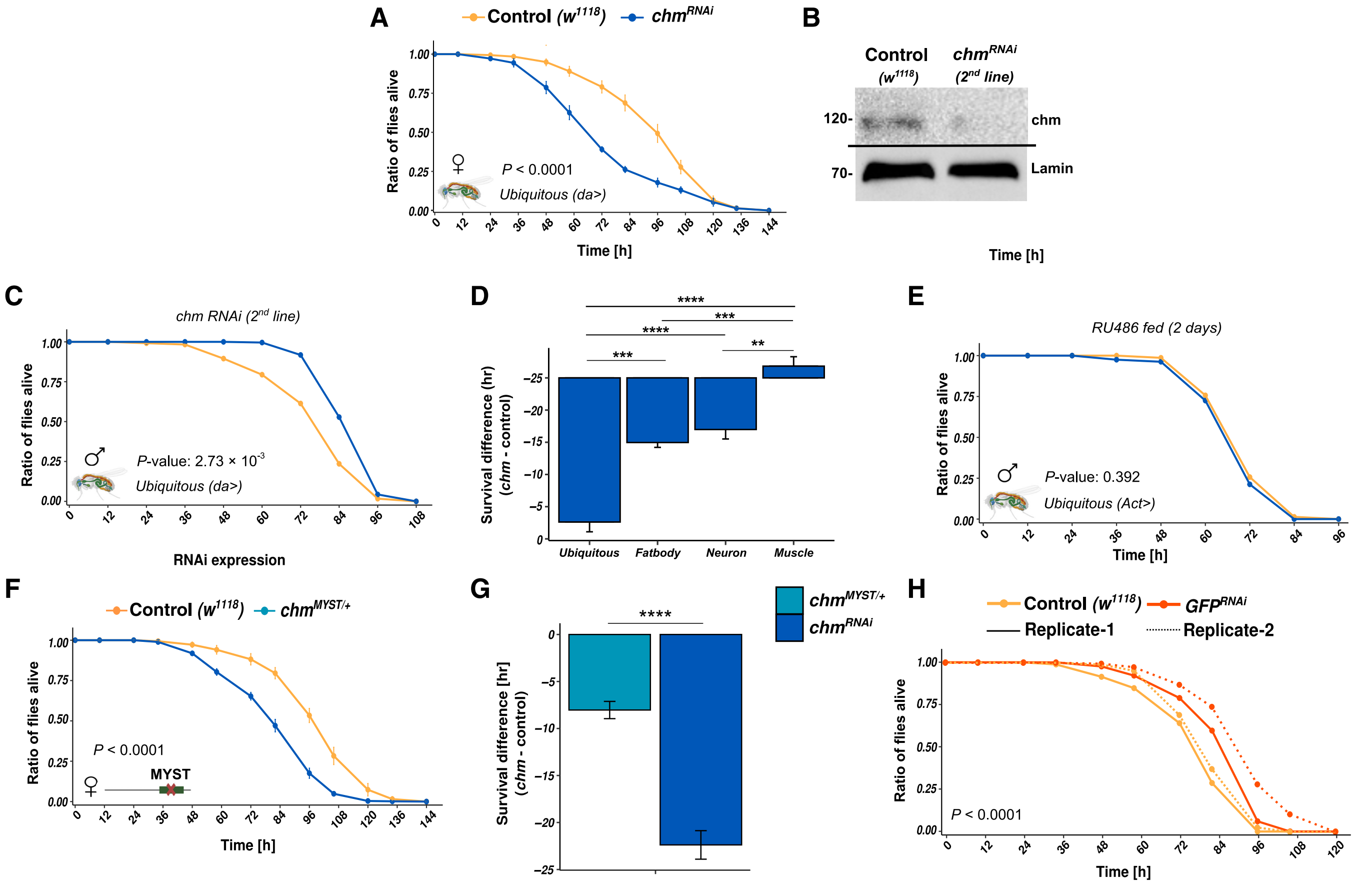

Figure EV3. Starvation susceptibility in chm mutants is observed independent of gender and RNAi line but not upon 2-day RU486 treatment.

A Average survival curve of control (w^{1118}) and chm^{RNAi} female flies upon ubiquitous knockdown of *chm* with *da-Gal4* (N = 4, paired).

B Western blot of chm for control (w^{1118}) and $chm^{RNAi\text{-}2}$.

C Survival curve of control (w^{1118}) and $chm^{RNAi\text{-}2}$ male flies upon ubiquitous knockdown of *chm* with *da-Gal4* (N = 1).

D Survival difference of chm knockdown using different drivers (ANOVA P-value: 2.73×10^{-07}, N = 3 (neuron- and muscle-specific) and 4 (ubiquitous and fat body-specific); Tukey *post-hoc* test was performed for statistical significance).

E Survival curve of control (w^{1118}) and chm^{RNAi} male flies upon RU486 administration for 2 days before starvation (N = 1).

F Average survival curve of control (w^{1118}) and $chm^{MYST/+}$ female flies (N = 4, paired).

G Survival difference between ubiquitous chm^{RNAi} and $chm^{MYST/+}$ male flies (N = 4 (RNAi) and 5 (MYST/+), unpaired t-test was performed for statistical significance).

H Survival curves of control (w^{1118}) and GFP^{RNAi} male flies upon ubiquitous GFP expression with *da-Gal4* (N = 2, paired). Each independent biological replicate is indicated by the solid and dotted lines.

Data information: All replicates are independent biological replicates and error bars indicate standard error of the mean (SEM). Unpaired t-test or Tukey test was performed as indicated and non-significant values are not shown (*$P < 0.05$, **$P < 0.01$, ***$P < 0.001$, ****$P < 0.0001$). For survival curves, log-rank test was performed for each biological replicate. The displayed P-value is based on all biological replicates.
Source data are available online for this figure.

 © 2023 The Authors

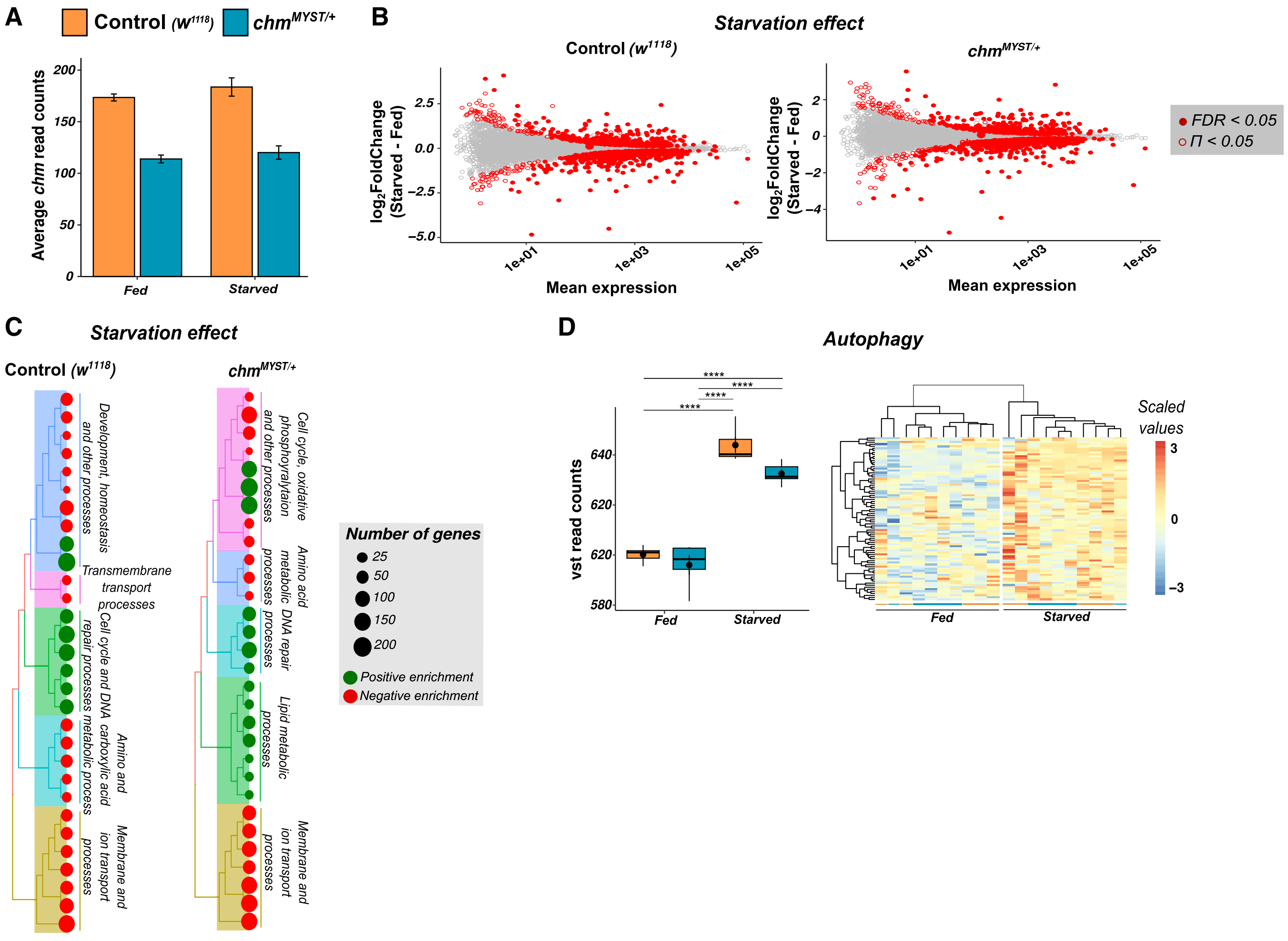

Figure EV4. Transcriptomic data show enrichment of similar genes and GO terms upon starvation independent of the genotype.

A Bar plot showing the average read counts of *chm* at fed and starved in control (w^{1118}) and $chm^{MYST/+}$ flies ($N = 5$, unpaired).

B MA plot of control (w^{1118}) and $chm^{MYST/+}$ flies in response to starvation. Significant genes are highlighted in filled and unfilled red circles for FDR < 0.05 and $\Pi < 0.05$, respectively.

C Tree plot depicting top 30 significant GO terms of starvation effect from GSEA of control (w^{1118}) and $chm^{MYST/+}$ transcriptome. Color on the circles indicates enrichment and the size indicates number of genes annotated with that pathway. GO terms were clustered based on semantic similarity and the terms that were represented the most within a cluster were mentioned.

D Box plot (left) and heatmap (right) of annotated genes from GO term autophagy (ANOVA *P*-value: 2.10×10^{-09}). Box plot shows the average vst normalized read counts of all annotated genes within the GO term averaged over the replicates. Boxes indicate interquartile range with central band as the median, central filled dot as the average, and the whiskers indicating the maximum and minimum values across five biological replicates. Heatmap shows the scaled values of vst normalized read counts in each replicate for all conditions.

Data information: All replicates are independent biological replicates and error bars indicate standard error of the mean (SEM). ANOVA followed by Tukey test was performed for box plot, and non-significant values are not shown (*$P < 0.05$, **$P < 0.01$, ***$P < 0.001$, ****$P < 0.0001$).
Source data are available online for this figure.

© 2023 The Authors

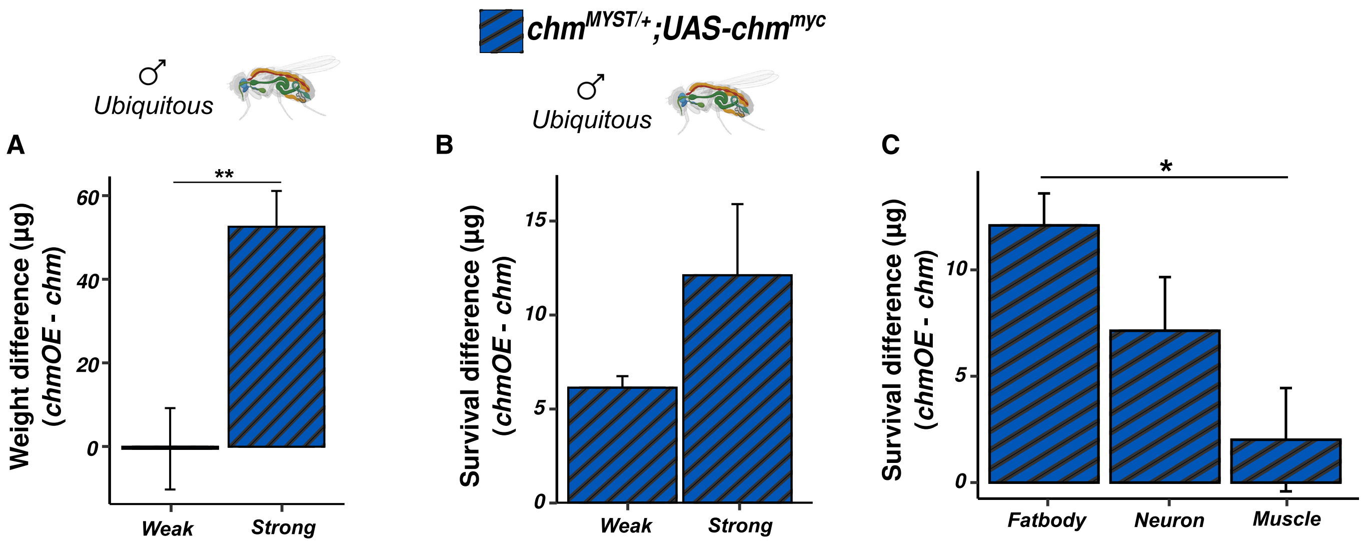

Figure EV5. chm's function in starvation susceptibility is validated by improvement of the phenotype upon overexpression.

A Weight difference in *chm*$^{MYST/+}$;*UAS-chm*^{myc} between weak and strong ubiquitous overexpressing male flies (*N* = 4 (strong) and 5 (weak), unpaired *t*-test was performed for statistical significance).

B Survival difference in *chm*$^{MYST/+}$;*UAS-chm*^{myc} between weak and strong ubiquitous overexpressing male flies (*N* = 3 (strong) and 4 (weak), unpaired *t*-test was performed for statistical significance).

C Survival difference in *chm*$^{MYST/+}$;*UAS-chm*^{myc} between different tissue-specific overexpressing male flies (*N* = 4 (fat body-specific) and 5 (neuron- and muscle-specific), unpaired, ANOVA *P*-value: 0.036; Tukey *post-hoc* test was performed for statistical significance).

Data information: All replicates are independent biological replicates and error bars indicate standard error of the mean (SEM). Unpaired *t*-test or Tukey test was performed as indicated and non-significant values are not shown (*$P < 0.05$, **$P < 0.01$, ***$P < 0.001$, ****$P < 0.0001$).
Source data are available online for this figure.

 © 2023 The Authors

Table of contents

Appendix Figure S1 1
Appendix Figure S2 2

Appendix Figure S1

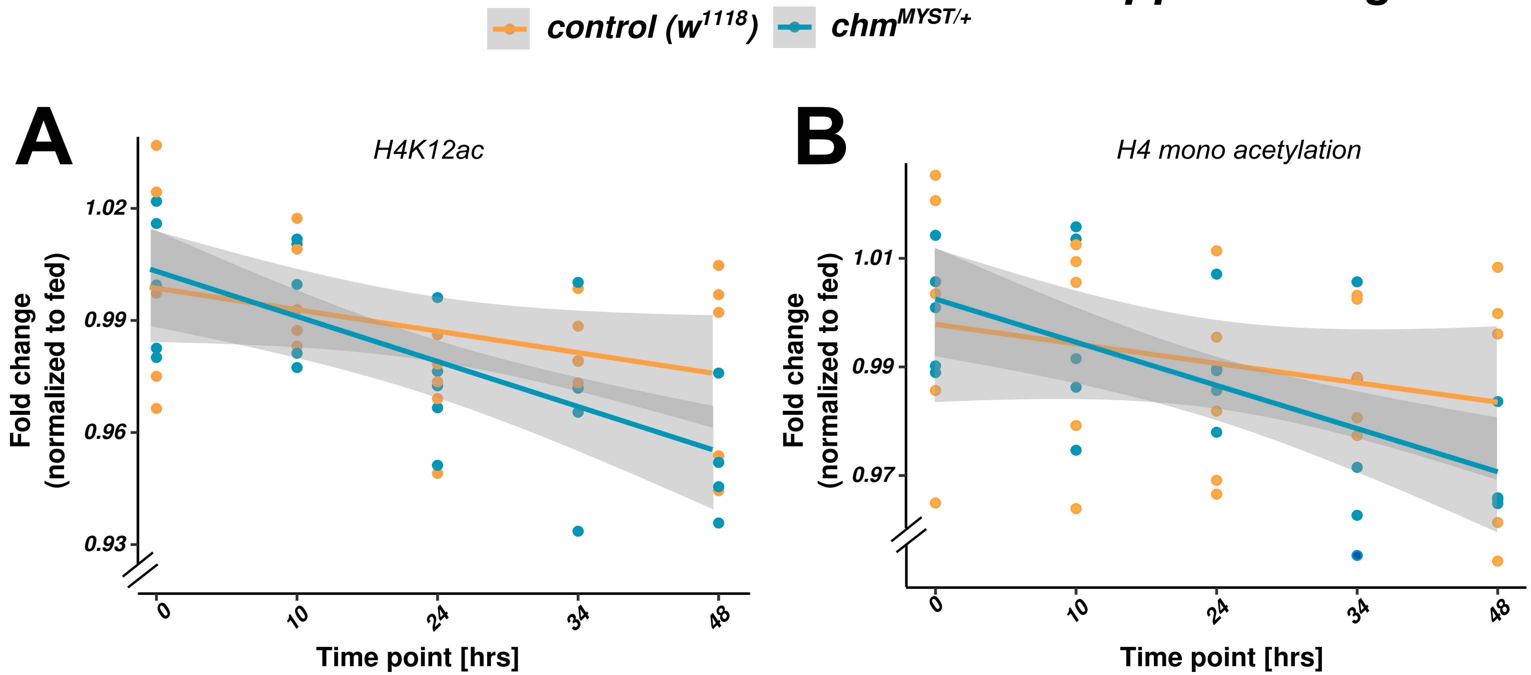

Appendix Figure S1: *Histone acetylation shows a decreasing trend in both control and $chm^{MYST/+}$ flies upon starvation.* Linear model showing mass-spectrometry quantified levels of A) H4K12ac and B) H4 mono-acetylation in control (w^{1118}) and $chm^{MYST/+}$ male flies across different starvation time points. Relative percentages of histone PTM were normalized to fed of corresponding genotype (N = 5, unpaired). Non-significant values are not indicated.

Appendix Figure S2

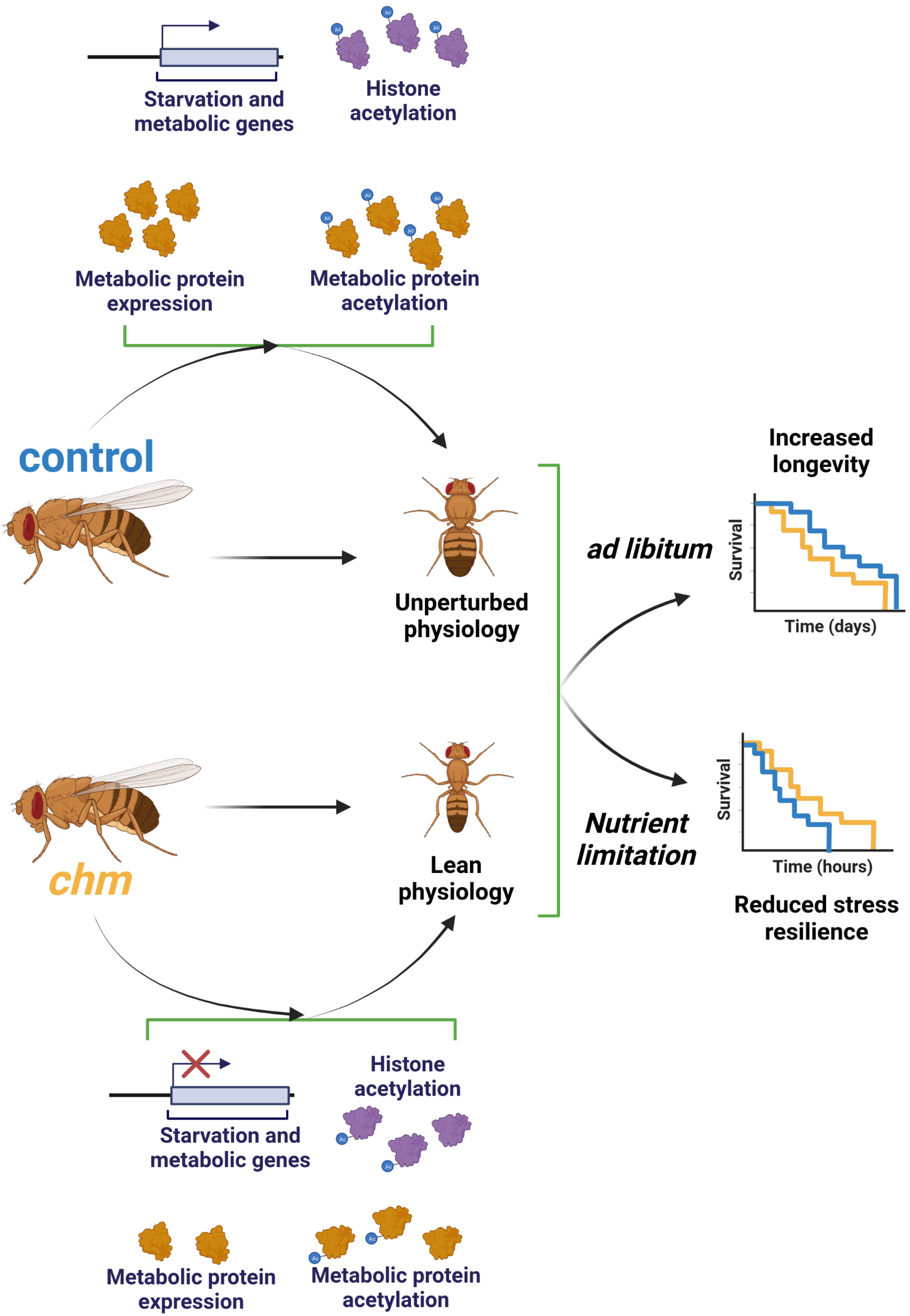

Appendix Figure S2: A decreased chm activity results in a lean phenotype and down regulation of genes and proteins involved in metabolic processes. While this results in life time extension in times of plenty, it is detrimental when food is sparse.
