# Supplementary figures and images for: The fruit fly acetyltransferase chameau promotes starvation resilience at the expense of longevity
Source: EMBO Rep. 2023 Sep 19;24(10):e57023. doi: 10.15252/embr.202357023 (PMC10561354; doi:10.15252/embr.202357023)

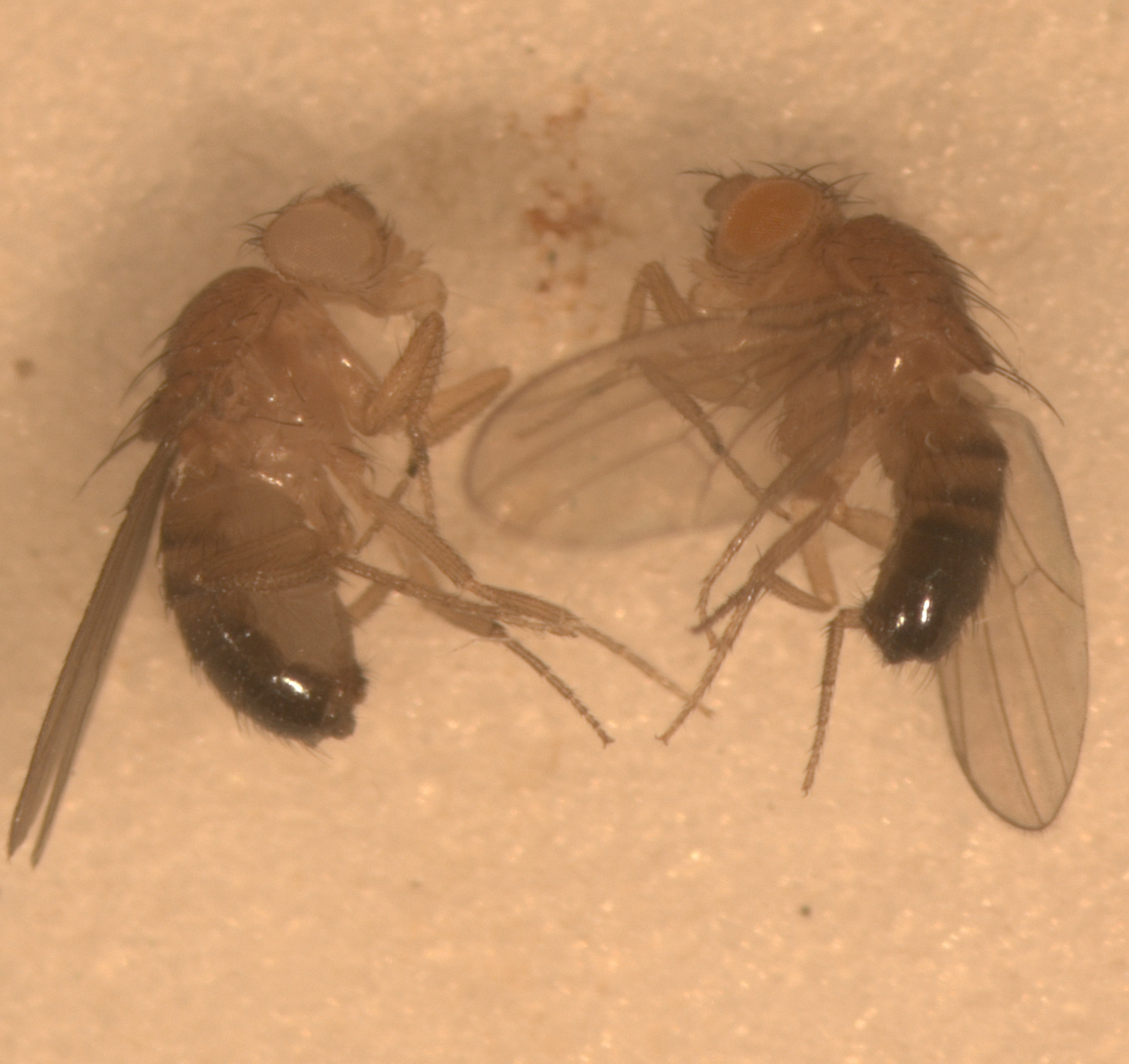

Supplement: Supplementary file 16 — Source Data for Figure 4 [file EMBR-24-e57023-s004.zip › Figure-1/1A/control-vs-chmRNAi_Ubiquitous.tif]

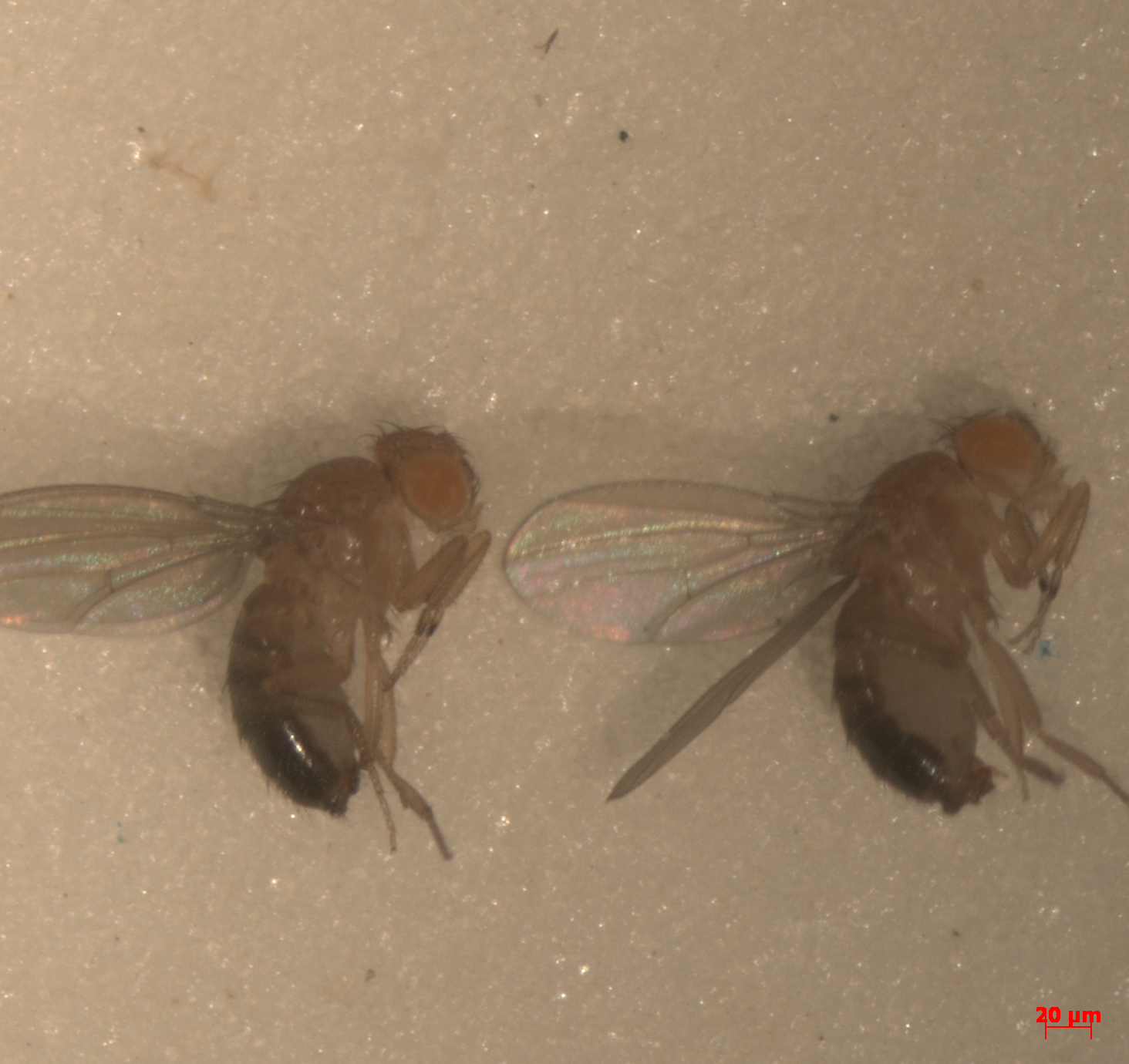

Supplement: Supplementary file 16 — Source Data for Figure 4 [file EMBR-24-e57023-s004.zip › Figure-1/1B/control-vs-chmRNAi_Fatbody.tif]

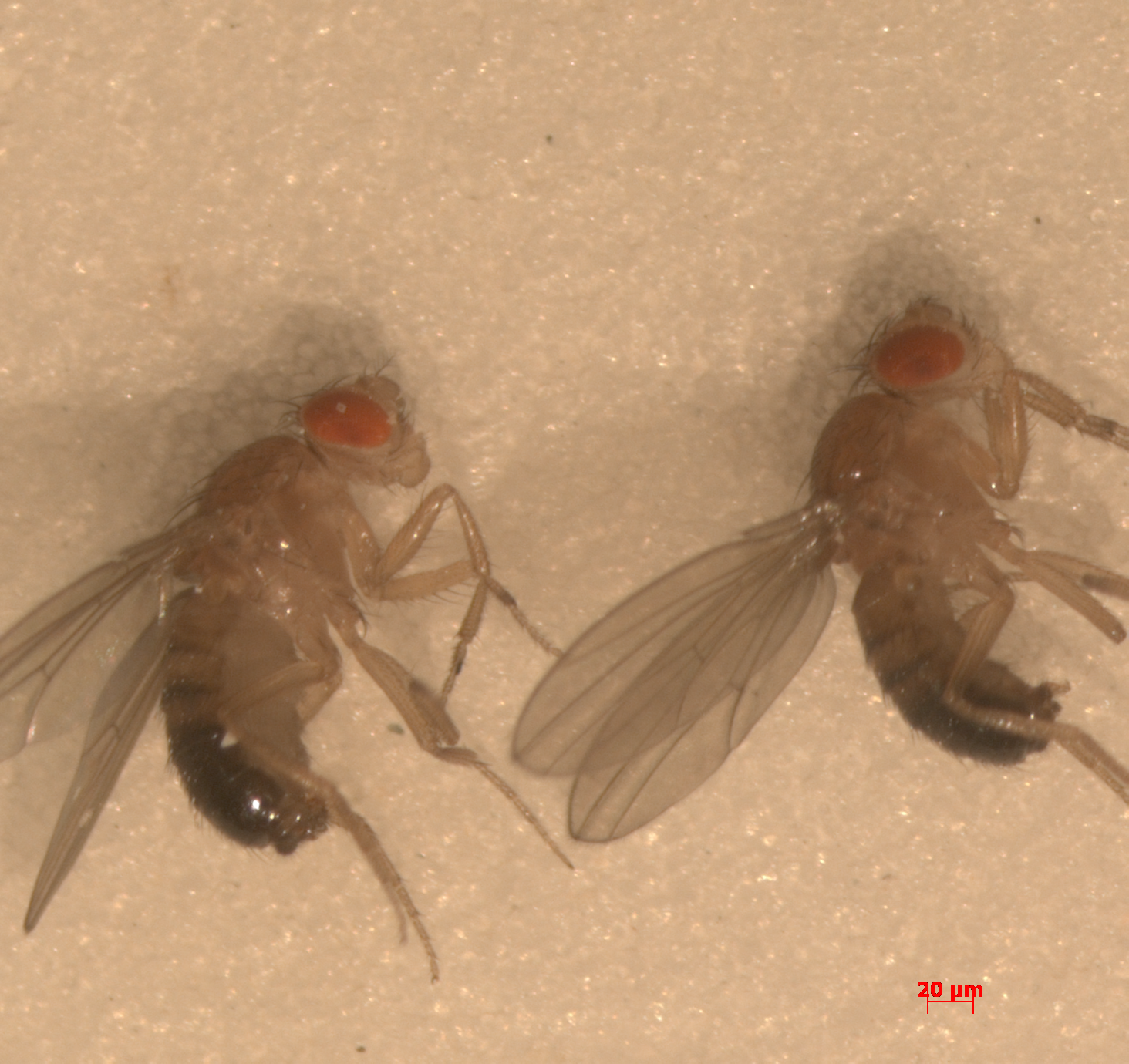

Supplement: Supplementary file 16 — Source Data for Figure 4 [file EMBR-24-e57023-s004.zip › Figure-1/1C/control-vs-chmRNAi_Neuron.tif]

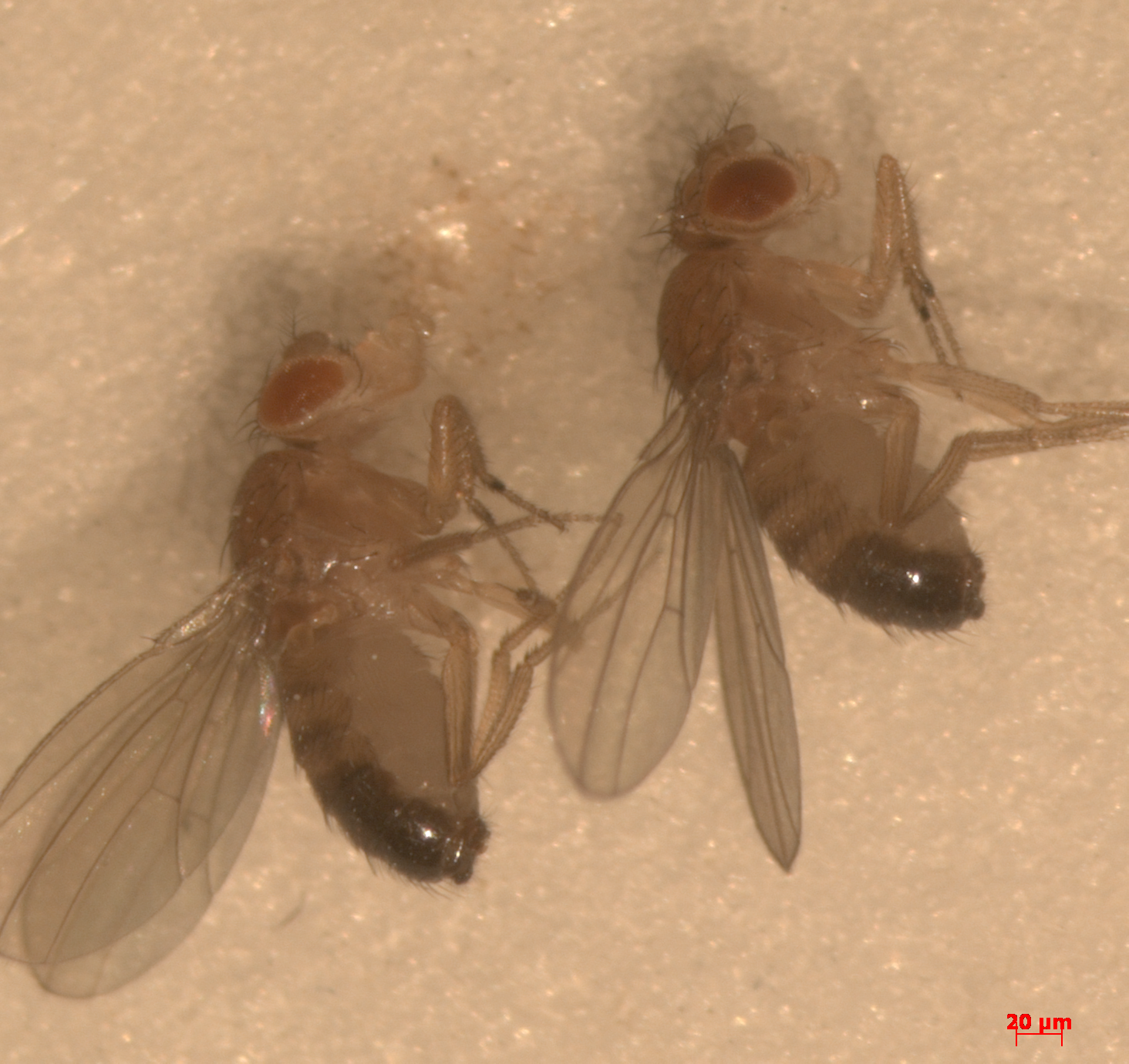

Supplement: Supplementary file 16 — Source Data for Figure 4 [file EMBR-24-e57023-s004.zip › Figure-1/1D/control-vs-chmRNAi_Muscle.tif]

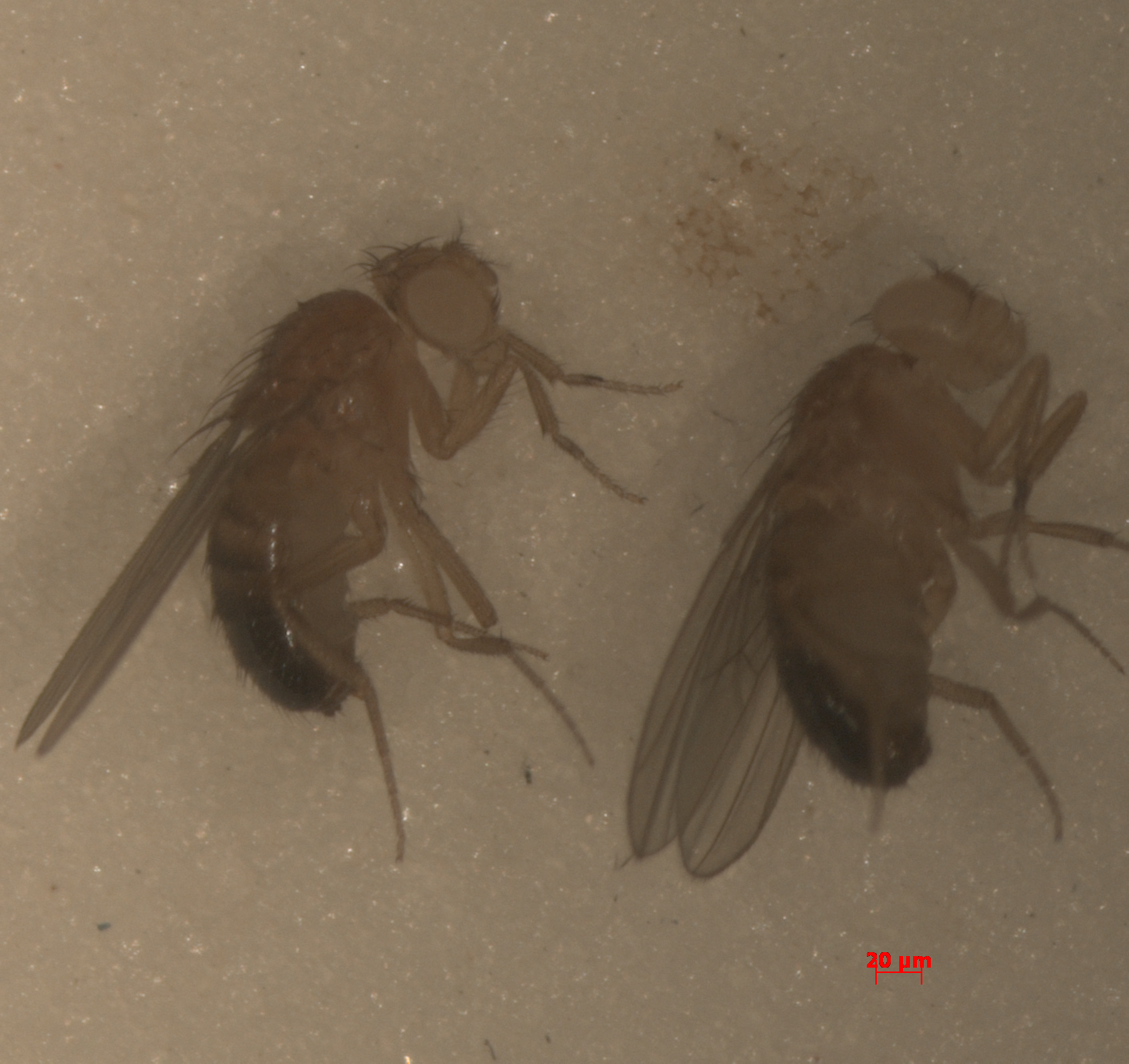

Supplement: Supplementary file 16 — Source Data for Figure 4 [file EMBR-24-e57023-s004.zip › Figure-1/1E/control-vs-chmMYST.tif]
