# Supplementary material for: The fruit fly acetyltransferase chameau promotes starvation resilience at the expense of longevity
Source: EMBO Rep. 2023 Sep 19;24(10):e57023. doi: 10.15252/embr.202357023 (PMC10561354; doi:10.15252/embr.202357023)
Supplement: Supplementary file 19 [file EMBR-24-e57023-s014.pdf]

EMBO reports

The fruit fly acetyltransferase Chameau promotes starvation resilience at the expense of longevity

Anuroop Venkateswaran Venkatasubramani, Toshiharu Ichinose, Mai Kanno, Ignasi Forne, Hiromu Tanimoto, Shahaf Peleg, and Axel Imhof
DOI: 10.15252/embr.202357023

Corresponding author(s): Axel Imhof (imhof@lmu.de) , Shahaf Peleg (peleg@fbn-dummerstorf.de)

Review Timeline:

Submission Date:	22nd Feb 23
Editorial Decision:	31st Mar 23
Revision Received:	27th Jul 23
Editorial Decision:	18th Aug 23
Revision Received:	22nd Aug 23
Accepted:	25th Aug 23

Editor: Ioannis Papaioannou

Transaction Report:

(Note: With the exception of the correction of typographical or spelling errors that could be a source of ambiguity, letters and reports are not edited. Depending on transfer agreements, referee reports obtained elsewhere may or may not be included in this compilation. Referee reports are anonymous unless the Referee chooses to sign their reports.)

Dear Dr. Imhof,

Thank you for submitting your research manuscript for consideration by EMBO reports. It has now been seen by four experts in the field, and we have received the full set of their reports, which are included below.

As you will see, the referees acknowledge that the findings are potentially interesting and the manuscript well-written. However, they also identify several limitations in the study and raise concerns regarding insufficient support of the conclusions by the available data, overinterpretation of some data, little mechanistic insight, and many open questions. The referees provide a number of helpful suggestions for further improvement and strengthening of the work and the manuscript, which should be addressed.

Given these constructive and well-informed reports and suggestions, we would like to invite you to revise your manuscript with the understanding that the referee concerns (as detailed in their reports) must be fully addressed and their suggestions taken on board. Please address all referee concerns in a complete point-by-point response. Acceptance of the manuscript will depend on a positive outcome of a second round of review. It is EMBO reports policy to allow a single round of revision only and acceptance or rejection of the manuscript will therefore depend on the completeness of your responses included in the next, final version of the manuscript. If you have any questions or comments, we can also discuss the revisions in a video chat, if you like.

We realize that it is difficult to revise to a specific deadline. In the interest of protecting the conceptual advance provided by the work, we usually recommend a revision within 3 months (June 30th). Please discuss with us the revision progress ahead of this time if you require more time to complete the revisions.

Please note that you can publish your study either as a Report or as an Article. For Reports, the manuscript should not exceed 27,000 characters (including spaces but excluding Materials & Methods and References) and 5 main plus 5 Expanded View figures. The Results and Discussion sections must be combined, which will help to shorten the manuscript text by eliminating some redundancy that is inevitable when discussing the same experiments twice. For an Article there are no length limitations, but it should have more than 5 main figures and the Results and Discussion sections must be separate. In both cases, the entire Materials and Methods must be included in the main manuscript file.

*****
IMPORTANT NOTE:
We perform an initial quality control of all revised manuscripts before re-review. Your manuscript will FAIL this control and the handling will be DELAYED if the following APPLIES:

1) If a data availability section providing access to data deposited in public databases is missing.

2) If your manuscript contains statistics and error bars based on n=2. Please use scatter plots in these cases. No statistics should be calculated if n=2.

When submitting your revised manuscript, please carefully review the instructions that follow below. Failure to include requested items will delay the evaluation of your revision.
*****

When submitting your revised manuscript, we will require:

1) A .docx formatted version of the manuscript text (including legends for main figures, EV figures and tables). Please make sure that the changes are highlighted to be clearly visible.

2) Individual production quality figure files as .eps, .tif, .jpg (one file per figure).
Please download our Figure Preparation Guidelines (figure preparation pdf) from our Author Guidelines pages https://www.embopress.org/page/journal/14693178/authorguide for more info on how to prepare your figures.

3) A .docx formatted letter INCLUDING the reviewers' reports and your detailed point-by-point responses to their comments. As part of the EMBO Press transparent editorial process, the point-by-point response is part of the Review Process File (RPF), which will be published alongside your paper unless you opt out of this (please see below for further information).

4) A complete author checklist, which you can download from our author guidelines (). Please insert information in the checklist that is also reflected in the manuscript. The completed author checklist will also be part of the RPF (please see below for more information).

5) Please note that all corresponding authors are required to supply an ORCID ID for their name upon submission of a revised manuscript (). Please find instructions on how to link your ORCID ID to your account in our manuscript tracking system in our Author guidelines
()

6) We replaced Supplementary Information with Expanded View (EV) Figures and Tables that are collapsible/expandable online. A maximum of 5 EV Figures can be typeset. EV Figures should be cited as 'Figure EV1, Figure EV2" etc... in the text and their respective legends should be included in the main text after the legends of regular figures.

- For the figures that you do NOT wish to display as Expanded View figures, they should be bundled together with their legends in a single PDF file called *Appendix*, which should start with a short Table of Content. Appendix figures should be referred to in the main text as: "Appendix Figure S1, Appendix Figure S2" etc. See detailed instructions regarding expanded view here:

- Additional Tables/Datasets should be labeled and referred to as Table EV1, Dataset EV1, etc. Legends have to be provided in a separate tab in case of .xls files. Alternatively, the legend can be supplied as a separate text file (README) and zipped together with the Table/Dataset file.

7) Before submitting your revision, primary datasets (and computer code, where appropriate) produced in this study need to be deposited in appropriate public databases (see <
https://www.embopress.org/page/journal/14693178/authorguide#dataavailability>).
Specifically, we would kindly ask you to provide public access to the following datasets/data:
- Mass spectrometry data.
- RNA sequencing data.

Please remember to provide a reviewer password if the datasets are not yet public.

The accession numbers and database should be listed in a formal "Data availability " section (placed after Materials & Methods) that follows the model below (see also < https://www.embopress.org/page/journal/14693178/authorguide#dataavailability>):

Data availability

The datasets (and computer code) produced in this study are available in the following databases:

- RNA-seq data: Gene Expression Omnibus GSE46843 (https://www.ncbi.nlm.nih.gov/geo/query/acc.cgi?acc=GSE46843)
- [data type]: [name of the resource] [accession number/identifier/doi] ([URL or identifiers.org/DATABASE:ACCESSION])

*** Note: all links should resolve to a page where the data can be accessed. ***

*** Note: the Data Availability Section is restricted to new primary data that are part of this study. ***

8) We request authors to consider both actual and perceived competing interests. Please review the new policy () and update your competing interests statement if necessary. Please name this section 'Disclosure and competing interests statement' and place it after the Acknowledgements section.

9) Figure legends and data quantification:
The following points must be specified in each figure legend:

- the name of the statistical test used to generate error bars and P values,
- the number (n) of independent experiments (please specify technical or biological replicates) underlying each data point,
- the nature of the bars and error bars (s.d., s.e.m.)
- If the data are obtained from n {less than or equal to} 2, use scatter plots showing the individual data points.

Discussion of statistical methodology can be reported in the Materials and Methods section, but figure legends should contain a basic description of n, P and the test applied.

See also the guidelines for figure legend preparation:
https://www.embopress.org/page/journal/14693178/authorguide#figureformat

10) We now request publication of original source data with the aim of making primary data more accessible and transparent to the reader. Our source data coordinator will contact you to discuss which figure panels we would need source data for and will also provide you with helpful tips on how to upload and organize the files.

11) Our journal encourages inclusion of *data citations in the reference list* to directly cite datasets that were re-used and obtained from public databases. Data citations in the article text are distinct from normal bibliographical citations and should directly link to the database records from which the data can be accessed. In the main text, data citations are formatted as follows: "Data ref: Smith et al, 2001" or "Data ref: NCBI Sequence Read Archive PRJNA342805, 2017". In the Reference list, data citations must be labeled with "[DATASET]". A data reference must provide the database name, accession number/identifiers and a resolvable link to the landing page from which the data can be accessed at the end of the reference. Further instructions are available at .

12) Please also note our reference format:
.

13) We now use CRediT to specify the contributions of each author in the journal submission system. CRediT replaces the author contribution section, which should be removed from the manuscript. Please use the free text box to provide more detailed descriptions. See also guide to authors:
.

14) As part of the EMBO publications' Transparent Editorial Process, EMBO reports publishes online a Review Process File to accompany accepted manuscripts. This File will be published in conjunction with your paper and will include the referee reports, your point-by-point response and all pertinent correspondence relating to the manuscript.

You can opt out of this by letting the editorial office know (emboreports@embo.org). If you do opt out, the Review Process File link will point to the following statement: "No Review Process File is available with this article, as the authors have chosen not to make the review process public in this case."

We would also welcome the submission of cover suggestions, or motifs to be used by our Graphics Illustrator in designing a cover.

I look forward to seeing a revised version of your manuscript when it is ready. Please let me know if you have any questions or comments regarding the revision.

Yours sincerely,

Ioannis Papaioannou, PhD
Editor
EMBO reports

Referee #1:

An interesting and technically well done manuscript from the Imhof lab. I wonder why the authors did not measure autophagy and lipolysis in starved/non-starved conditions in Chameau proficient and -depleted flies in order to underline their hypothesis.

Referee #2:

This group have previously shown that mutations that disrupt the histone acetyltransferase Chameau result in lifespan extension in Drosophila. Here, they expand on these findings to show that decreased chm levels/activity decrease fly weight and starvation resistance, potentially through misregulation of genes involved in energy storage and metabolism. Although this is an interesting idea, in my opinion the data presented do not strongly support these conclusions because of a lack of appropriate controls and overinterpretation of the gene expression data. Based on the data provided, it might be worth considering non-histone targets (i.e. not gene expression) in terms of mechanism. Overall, the gene expression changes between chm mutants and control are relatively minor and the authors have used some questionable analysis approaches to connect these to energy storage/metabolism. In addition, the genetic controls used for RNAi experiments are not appropriate and more than one RNAi line should be used to account for potential off-target effects. Last, the fact that these phenotypes are only observed at temperatures below 23C seems a bit concerning - this doesn't seem to be a very robust set of phenotypes or gene expression changes. Last, many of the details required to assess the data are absent eg western blots are not shown, RNA-seq data

should be available on GEO or another database. Specific comments with figure references are provided below:
1. W1118 is not an appropriate control for the RNAi experiments (eg Fig. 1). Instead, the authors should compare with a non-specific RNAi expressed under the same Gal4 driver (several VALIUM controls are available that target non-Drosophila genes like GFP etc). This is important because expression of RNAi constructs (or Gal4 drivers) can have effects both on gene expression and phenotypes. In addition, more than one RNAi construct against chm should be used to account for potential off-target effects. The identity of controls also needs to be clearly described in the figure itself so readers can easily identify these.
2. I am not convinced that chm knockdown results in a reduced ability of flies to store energy (line 100) because the knockdown and mutants would both impact development. Adult-specific knockdown or loss of function (post-development) would be necessary to make these claims.
3. Western blots for the H4K12ac level decrease (supp Fig. 2) are not shown, and it's unclear what the y axis represents on the graphs. How were these data normalized and quantified?
4. The authors used an FDR < 0.1 for all DEGs in the RNA-seq analysis (rather than 0.05, which would be fairly standard), and it's clear from looking at the MA plots and supp Tables that relatively few genes show changes in gene expression. In addition, the variation between individual samples on the PCA plot in supp Fig. 3a is pretty substantial. What was the correlation between the individual biological replicates? I think the gene expression data shown are not strong enough to support the conclusion that chm plays an important role in adaption of gene expression patterns to nutrient limiting conditions (line 136). Moreover, comparing the overall read counts of genes associated with select GO terms (Fig. 3e-g) is simply not an appropriate analysis approach. It would be more helpful to show individual genes across all 4 samples in a bar graph if there are genes that show these patterns i.e. dampening of transcriptional response. Or perhaps present these as a heatmap of normalized expression scores.
5. What are the x-axis labels on supp Fig. 4a?
6. Are the RNA-seq data deposited in GEO or a similar database?
7. I am a bit concerned that the phenotype is only observed at temperatures <23C (line 120). Together with the variability in gene expression data, this gives me some concerns about technical artifacts that could be affecting interpretation of these experiments.

Referee #3:

In their manuscript, Venkateswaran and colleagues adds to their previous findings on the impact of histone acetyltransferase chm (HBO1 in mammals) on aging. They found that while chm silencing extends life expectancy in well fed drosophila flies, it restrains their ability to cope with nutrient deprivation. In fact, chm silencing leads to decrease in body weight and shorter lifespan under starvation.
The study has little mechanistic insight, but the authors could demonstrate that these effects are linked to the catalytic activity of chm, as the same effects were observed upon expression of catalycally-inactive chm.
Overall, the manuscript is well written and has significant impact for the understanding of the evolutionary significance of chm. Weight and lifespan effects are clear. The main conclusion is that chm is possibly important to regulate energy storage, possibly at the neuronal level. Observations are very interesting although mechanistic insight is minimal.
With that in mind, some aspects might still be improved to solidify the main conclusion of the study.

First, defective energy storage needs to be interrogated a bit more in depth. Authors should clarify that is not due to neurological impairments: do these flies move/fly OK? Do the feed/forage normally?
Authors should also test for growth defects. That should give a better sense whether chm indeed cause specific defects in energy storage.
It would be great if the authors could measure muscle (and/or fat) mass, but this is maybe beyond the scope of revision.
Next, authors should clarify the contribution of the neuronal system. Is the brain defective in sensing energy stress? Can they check AMPK activation for example?

One technical aspect the authors should clarify is how they normalized reads count when calculating "average reads count". The results is possibly interesting but normalization is an obvious issue when interrogating global deregulation of transcription (total read count change accordingly).
As a general note, there are more powerful bioinformatic tools to dissect differences in gene expression levels.

Minor concerns include:
- life expectancy (less than 100 hours) is markedly different from what they observed in their previous papers (weeks). Am I missing anything? What is the difference about?
- Authors should show chm levels are in fact down in RNAi muscles
- any notable difference in regulated genes between stared and fed conditions? More in general, comparison of effects in the two states is not easy because data are broken down in different figures.
- effects on acetylation under starvation are not reported
- several typos and vocabulary is not homogeneous in the text (ex: spelling of "lifespan")

Referee #4:

In the manuscript "The Drosophila acetyltransferase 1 chameau (chm) promotes starvation resilience at the expense of a shortened life span", Venkatasubramani et al. investigate the effect of mutating the acetyltransferase chm on the life span and starvation response in Drosophila. The authors find that although disruption of chm activity has a positive effect on increasing the life span of flies, it comes at the cost of the ability to deal with metabolic stress conditions. The manuscript overall is well written and is easy to follow. However, the manuscript fails to explore the causality behind the observed phenotypes, and is unable to support the author's speculations with any supportive evidence. As such, it is not clear that the findings presented in the manuscript substantially increase our understanding of how chm governs the phenotype of fly.

1. The RNA-seq data reveals that the genes that are differentially expressed belong to metabolic and related processes, which is expected as weight loss is observed in these flies. This is a good start, but unfortunately the authors don't follow up on this to provide mechanistic answers of how chm disruption is bringing about these changes.
2. The authors found that the acetyltransferase activity of chm is responsible for the phenotypes they observed. However, in the work presented, the important question remains unexplored that which substrate acetylation governs this phenotype and how.
3. Strikingly, the disruption of chm only in the neurons is sufficient to cause the phenotype that is attributed to metabolic rewiring. This is an important question to answer. However, the authors do not pursue this and leave it to speculation that it might be a result of behavioral changes in the flies.
4. For RNA-Seq, the authors used fly heads. Yet, surprisingly, they found changes in processes belonging to metabolism. The authors do not talk about any gene expression changes that might contribute to the speculated behavioral changes.

Detailed Response to the reviewers' comments:

Referee #1:

An interesting and technically well done manuscript from the Imhof lab. I wonder why the authors did not measure autophagy and lipolysis in starved/non-starved conditions in Chameau proficient and -depleted flies in order to underline their hypothesis.

We would like to thank the reviewer for their appreciation of our work. In the revised version, we show that changes in the expression of genes associated with autophagy is only dependent on nutrient status but not on the expression level of chm (Fig EV4D). In addition, genes involved in lipid metabolism did show a dampened transcriptional response towards starvation in chm mutant flies (Fig 5G). As with all the effects we see, the differences are rather moderate and modulatory in nature. We therefore conclude that the observed phenotype of a lack of chm activity is rather a combination of its moderate effect on many metabolic pathways rather than a strong one on single ones. We therefore focused our efforts on the question whether the starvation phenotype is caused by a reduction of chm activity earlier in life or whether chm is acutely needed for a response to starvation.

Referee #2:

This group have previously shown that mutations that disrupt the histone acetyltransferase Chameau result in lifespan extension in Drosophila. Here, they expand on these findings to show that decreased chm levels/activity decrease fly weight and starvation resistance, potentially through misregulation of genes involved in energy storage and metabolism. Although this is an interesting idea, in my opinion the data presented do not strongly support these conclusions because of a lack of appropriate controls and overinterpretation of the gene expression data. Based on the data provided, it might be worth considering non-histone targets (i.e. not gene expression) in terms of mechanism.

We agree with the reviewer that the effect of chm is not necessarily direct and may involve transcriptional as well as posttranscriptional aspects. Indeed, when we investigated the effect of chm on the proteome or the acetylome we observed changes that could not be explained by changes in the transcriptome only. However, the proteins that changed upon chm knock down either in amount or in their level of acetylation were also associated with GO terms related to metabolism (Fig 3).

Overall, the gene expression changes between chm mutants and control are relatively minor and the authors have used some questionable analysis approaches to connect these to energy storage/metabolism. In addition, the genetic controls used for RNAi experiments are not appropriate and more than one RNAi line should be used to account for potential off-target effects.

We agree that the changes between controls and chm mutants are relatively small. That is true for the observed transcriptional changes and is probably due to the use of a MYST chm mutant. For the changes in the proteome and the acetylome, using a line with stronger chm reduction, the proteins affected were connected to metabolic processes (Fig 3). In addition, we have now included an additional RNAi line in the revised manuscript (Fig EV3C).

Last, the fact that these phenotypes are only observed at temperatures below 23C seems a bit concerning - this doesn't seem to be a very robust set of phenotypes or gene expression changes.

We think this points to the role of chm as a modulator rather than the single main player. It is worth mentioning that such seemingly moderate temperature differences have been shown to have major effects on developmental timing, life span and metabolism (PMIDs: 30099070, 37094603, 30918312, 29478858, 27641694). The results were consistent over many independent biological replicates in different labs in Germany and Japan and independent of the sex of the flies. We have also tested a range of different temperatures and observe consistently that *chm*$^{MYST/+}$ flies show a temperature-dependent phenotype.

Last, many of the details required to assess the data are absent eg western blots are not shown, RNA-seq data should be available on GEO or another database.

All data are now provided either in the manuscript or as extended versions and extended tables. Proteomic data are deposited in the PROSITE database and NGS data are available on GEO. The

corresponding credentials for the reviewers are mentioned in the data availability section of the manuscript and are as follows:
Transcriptomic data: Gene Expression Omnibus GSE211042, (https://www.ncbi.nlm.nih.gov/geo/query/acc.cgi?acc=GSE211042; Reviewer token: ojctuwmqhbkvzeh). Histone PTM mass-spectrometry data: ProteomeXchange Consortium via the PRIDE[20] partner repository PXD035947 (https://www.ebi.ac.uk/pride/login; Reviewer account details: Username: reviewer_pxd035947@ebi.ac.uk, Password: kRLzPAqY).

Acetylome and proteome mass-spectrometry data: ProteomeXchange Consortium via the PRIDE[20] partner repository PXD042471 (https://www.ebi.ac.uk/pride/login; Reviewer account details: Username: reviewer_pxd042471@ebi.ac.uk, Password: yPPjlZG8).

Specific comments with figure references are provided below:
1. W1118 is not an appropriate control for the RNAi experiments (eg Fig. 1). Instead, the authors should compare with a non-specific RNAi expressed under the same Gal4 driver (several VALIUM controls are available that target non-Drosophila genes like GFP etc). This is important because expression of RNAi constructs (or Gal4 drivers) can have effects both on gene expression and phenotypes. In addition, more than one RNAi construct against chm should be used to account for potential off-target effects. The identity of controls also needs to be clearly described in the figure itself so readers can easily identify these.

We agree with the reviewer that w1118 is not the ideal control for the RNAi experiments. We therefore tested the starvation response using a GFP-RNAi line as suggested by the reviewer. In contrast to the chm RNAi line, the GFP-RNAi showed an even higher survival than the w1118 male flies. The corresponding data are now shown in figure EV3H. As the GFP-RNAi was not backcrossed with w1118, while da-gal4, the other RNAi lines and the MYST/+ mutant flies were, we used w1118 as control in all our experiments.

2. I am not convinced that chm knockdown results in a reduced ability of flies to store energy (line 100) because the knockdown and mutants would both impact development. Adult-specific knockdown or loss of function (post-development) would be necessary to make these claims.

To address the reviewer's question, we used a GeneSwitch system that enabled us to knock down chm in adults upon treatment with RU486 (mifepristone). In agreement with our hypothesis that chm is important for an efficient survival of starvation in a direct manner rather than a delayed development, we observed a reduced survival upon starvation after a 4 day treatment with RU486 in the chm gene switch line only.

3. Western blots for the H4K12ac level decrease (supp Fig. 2) are not shown, and it's unclear what the y axis represents on the graphs. How were these data normalized and quantified?

We apologize for not being clear in our previous version of the manuscript. We have now corrected this and mentioned clearly that H4K12ac was measured using mass-spectrometry and not Western blot in the corresponding figure legend.

4. The authors used an FDR < 0.1 for all DEGs in the RNA-seq analysis (rather than 0.05, which would be fairly standard), and it's clear from looking at the MA plots and supp Tables that relatively few genes show changes in gene expression. In addition, the variation between individual samples on the PCA plot in supp Fig. 3a is pretty substantial. What was the correlation between the individual biological replicates? I think the gene expression data shown are not strong enough to support the conclusion that chm plays an important role in adaption of gene expression patterns to nutrient limiting conditions (line 136). Moreover, comparing the overall read counts of genes associated with select GO terms (Fig. 3e-g) is simply not an appropriate analysis approach. It would be more helpful to show individual genes across all 4 samples in a bar graph if there are genes that show these patterns i.e. dampening of transcriptional response. Or perhaps present these as a heatmap of normalized expression scores.

We thank the author for raising this question. In fact, the top GO terms displayed in the figures have a FDR < 0.05, which is why we set there more common threshold of a FDR < 0.05 in the revised manuscript. We also used an additional parameter that includes the biological information, i.e. a combination of log2 fold-change and p-value, termed Π, with a cut-off < 0.05 (Xiao et al, 2014; Hostrup et al, 2022).

5. What are the x-axis labels on supp Fig. 4a?

Thank you for pointing this out. We have now corrected this and mentioned the corresponding nutrient status (Fig. EV4A).

6. Are the RNA-seq data deposited in GEO or a similar database?

See comment above

7. I am a bit concerned that the phenotype is only observed at temperatures <23C (line 120). Together with the variability in gene expression data, this gives me some concerns about technical artifacts that could be affecting interpretation of these experiments.

See comment above

Referee #3:

In their manuscript, Venkateswaran and colleagues adds to their previous findings on the impact of histone acetyltransferase chm (HBO1 in mammals) on aging. They found that while chm silencing extends life expectancy in well fed drosophila flies, it restrains their ability to cope with nutrient deprivation. In fact, chm silencing leads to decrease in body weight and shorter lifespan under starvation.
The study has little mechanistic insight, but the authors could demonstrate that these effects are linked to the catalytic activity of chm, as the same effects were observed upon expression of catalycally-inactive chm.
Overall, the manuscript is well written and has significant impact for the understanding of the evolutionary significance of chm. Weight and lifespan effects are clear. The main conclusion is that chm is possibly important to regulate energy storage, possibly at the neuronal level. Observations are very interesting although mechanistic insight is minimal.
With that in mind, some aspects might still be improved to solidify the main conclusion of the study.

We would like to thank the reviewer for their appreciation of our work

First, defective energy storage needs to be interrogated a bit more in depth. Authors should clarify that is not due to neurological impairments: do these flies move/fly OK? Do the feed/forage normally?

We tested the feeding behavior of the flies in both ubiquitous RNAi and MYST/+ mutants. While we observed reduced feeding in *chm*^{RNAi} flies upon ubiquitous knockdown, MYST/+ mutants showed no such differences. However, both cases showed a strong starvation susceptibility indicating that feeding behavior doesn't explain the reduced starvation resilience. Furthermore, previous studies from our lab have shown that *chm*$^{MYST/+}$ mutants are more active than its corresponding control of same age (Peleg, et al., 2016).

Authors should also test for growth defects. That should give a better sense whether chm indeed cause specific defects in energy storage.

We did not directly measure the growth defect, but we did observe a partial development arrest-like phenotype, in the da-GAL4 *chm*^{RNAi} line. However, this was not observed in any of the tissue specific lines or *chm*$^{MYST/+}$ mutants. This information is now incorporated in the revised manuscript.

It would be great if the authors could measure muscle (and/or fat) mass, but this is maybe beyond the scope of revision.

It would be indeed great to do this but as the differences in the intact flies are already moderate in the heterozygous flies, the putative mass differences could be less in the muscle/fat tissues making it difficult to recognize differences.

Next, authors should clarify the contribution of the neuronal system. Is the brain defective in sensing energy stress? Can they check AMPK activation for example?

Although in our previous version, we showed that the effect is mostly neuronal, our results indicate the the effect is stronger upon fatbody-specific RNAi of chm (Figs 1B, 1F and EV3D). Our proteomic data showed no change in AMPKalpha levels upon ubiquitous chm knockdown (Table EV3), which is why we did not further pursue AMPK as a major target of chm.

One technical aspect the authors should clarify is how they normalized reads count when calculating "average reads count". The results is possibly interesting but normalization is an obvious issue when interrogating global deregulation of transcription (total read count change accordingly).

As a general note, there are more powerful bioinformatic tools to dissect differences in gene expression levels.

We have now changed this to variance stabilizing transformed (vst) read counts which is a standard way of normalizing the read counts using DESeq2 analysis. Following this, we took the average of vst read counts of all the annotated genes in the selected GO term in one replicate and in each condition. This was done for all replicates giving 5 average vst read count values for every condition. This extensive analysis was done to avoid false statistical significance by designating the number of genes as “biological replicates”. In addition, we have also represented the data as a heatmap to better observe the moderate effect across replicates (Figs 5D-G and EV4D).

Minor concerns include:
- life expectancy (less than 100 hours) is markedly different from what they observed in their previous papers (weeks). Am I missing anything? What is the difference about?

In this manuscript, we measure lifespan upon starvation while in the previous one we determined life span when fed ad libitum. This could also be seen in Fig 4 and Fig EV1C with one showing the survival upon starvation and one when fed ad libitum, respectively.

- Authors should show chm levels are in fact down in RNAi muscles

While we have checked chm mRNA levels and the expression of dsRNA on the entire flies, we did not test the effectiveness of RNAi in the muscles. This was mainly due to the lack of an antibody with a sufficiently high enough sensitivity to detect chm from low input material.

- any notable difference in regulated genes between stared and fed conditions? More in general, comparison of effects in the two states is not easy because data are broken down in different figures.

As we now emphasize in the revised manuscript, we observed changes mainly on aa quantitative rather than a qualitative level. In fact, flies heterozygous for the *chmMYST* mutation show a very similar response in which genes respond to starvation but the amplitude of their response is greatly reduced. This dampened response is now shown in Figs 5D-G and EV4D. Interestingly, a similar inertia towards environmental changes has been shown earlier this year in epigenetically disrupted cancer cells by the Scaffidi group (Loukas et al., 2023), which we have now pointed out in the discussion.

- effects on acetylation under starvation are not reported

They are now reported in the revised MS for histone acetylation (Fig EV6A-B).

- several typos and vocabulary is not homogeneous in the text (ex: spelling of "lifespan")

All typos we could find are corrected in the revised MS

Referee #4:

In the manuscript "The Drosophila acetyltransferase 1 chameau (chm) promotes starvation resilience at the expense of a shortened life span", Venkatasubramani et al. investigate the effect of mutating the acetyltransferase chm on the life span and starvation response in Drosophila. The authors find that although disruption of chm activity has a positive effect on increasing the life span of flies, it comes at the cost of the ability to deal with metabolic stress conditions. The manuscript overall is well written and

is easy to follow. However, the manuscript fails to explore the causality behind the observed phenotypes, and is unable to support the author's speculations with any supportive evidence. As such, it is not clear that the findings presented in the manuscript substantially increase our understanding of how chm governs the phenotype of fly.

1. The RNA-seq data reveals that the genes that are differentially expressed belong to metabolic and related processes, which is expected as weight loss is observed in these flies. This is a good start, but unfortunately the authors don't follow up on this to provide mechanistic answers of how chm disruption is bringing about these changes.

In the revised manuscript we emphasized the fact that the chm induced changes in expression of genes belonging to metabolic processes are rather mild (Figs 5D-G and EV4D). Therefore, it was difficult to follow-up on specific genes or gene-sets. Moreover, chm exerts its function not only by regulating transcription but also via a modulation of protein levels and changes in non-histone acetylation. We show this in the revised manuscript by analyzing chm dependent changes in the proteome as well as the acetylome. In both cases we observe proteins involved in multiple metabolic processes as the major targets of chm (Figs 3B-D). In the revised manuscript, we also show that the effect of a fatbody-specific chm knockdown is stronger than the one observed upon a knockdown in neurons, which further supports the hypothesis that chm regulates metabolic processes.

2. The authors found that the acetyltransferase activity of chm is responsible for the phenotypes they observed. However, in the work presented, the important question remains unexplored that which substrate acetylation governs this phenotype and how.

As mentioned in the previous comment, we have also added acetylome data. Most of the proteins that showed differential acetylation are associated with0 metabolic processes suggesting that chm affects the PTMs of these proteins, presumably by gauging Acetyl-CoA levels. In future studies, we plan to focus on some of these substrates (such as Tps1, Idh etc) to delineate the metabolic role of chm.

3. Strikingly, the disruption of chm only in the neurons is sufficient to cause the phenotype that is attributed to metabolic rewiring. This is an important question to answer. However, the authors do not pursue this and leave it to speculation that it might be a result of behavioral changes in the flies.

As pointed out above, we show that the effect of fatbody-specific chm knockdown is stronger than a knockdown in neurons. In addition, we also tested the feeding behavior of the flies in both ubiquitous RNAi and MYST/+ mutants. While we observed reduced feeding in chm^{RNAi} flies upon ubiquitous knockdown, MYST/+ mutants showed no such differences. However, in both cases, flies with reduced chm or its activity showed starvation susceptibility indicating that feeding behavior doesn’t explain the reduced starvation resilience completely. Furthermore, previous studies from our lab have shown that $chm^{MYST/+}$ mutants are more active than its corresponding control of same age (Peleg et al, 2016).

4. For RNA-Seq, the authors used fly heads. Yet, surprisingly, they found changes in processes belonging to metabolism. The authors do not talk about any gene expression changes that might contribute to the speculated behavioral changes.

We used fly heads as they contain contain both neurons and fat cells (Fujii et al.,2002), which are the two tissues where we observed the strongest effect of a *chm* removal. For additional explanation, please also see the comments above.

Dear Dr. Imhof,

Thank you for submitting your revised manuscript to EMBO reports. We have now received the full set of reports from the four referees that were asked to re-evaluate your study. Please find their comments included below.

As you will see, all referees acknowledge that the revised manuscript has been strengthened by the addition of new data, and referees #1, #2, and #3 recognize that most of their previous concerns have been successfully addressed, although referee #3 also mentions that the mechanistic insight remains minimal. Referee #4, on the other hand, emphasizes the lack of in-depth mechanistic understanding of the phenomenon and evidence for the direct role of chm. Taking into consideration the input from all four referees, our editorial decision is that the advance provided by the study is sufficiently striking for publication in EMBO reports even without further mechanistic insight.

From the editorial side, there are several things that we need from you to address in a revised version of your manuscript before we can proceed with acceptance of your study for publication in EMBO reports:

- Please note that the title and the abstract of the revised manuscript should be brief, yet explicit, even to non-specialists. The length of the title should not exceed 100 characters (including spaces), and the abstract should be a single paragraph not exceeding 175 words. I took the liberty to edit them for you; please find my suggested title and abstract below and incorporate them in your revised manuscript if you agree with the suggestions, or feel free to improve them further according to the instructions above.

Suggested title:
The fruit fly acetyltransferase Chameau promotes starvation resilience at the expense of longevity

Suggested abstract:
Proteins involved in cellular metabolism and molecular regulation can extend lifespan in various organisms in the laboratory. However, any improvement in aging would only provide an evolutionary benefit if the organisms were able to survive under non-ideal conditions. We have previously shown that Drosophila melanogaster carrying a loss-of-function allele of the acetyltransferase chameau (chm) has an increased healthy lifespan when fed ad libitum. Here, we show that loss of chm and reduction of its activity result in a substantial reduction of weight and a decrease in starvation resistance. This phenotype is caused by failure to properly regulate the genes and proteins required for energy storage and expenditure. The previously observed increase in survival time thus comes with the inability to prepare for and cope with nutrient stress. As the ability to survive in environments with restricted food availability is likely a stronger evolutionary driver than the ability to live a long life, chm is still present in the organism's genome despite its apparent negative effect on lifespan.

- Please note the widely accepted conventions for Drosophila gene and protein nomenclature and follow them consistently throughout the manuscript.

- Your Figure legends have been inspected by our data editors for completeness and accuracy. Please see the required changes in the attached Word file and address all comments in your revised manuscript (with tracked changes).

- Please make sure that all deposited datasets in external repositories will be publicly available at the time of publication. The reviewer tokens for confidential access can now be removed from your Data availability statement.

- Please provide in the last column of your Author Checklist only the section(s) of the manuscript where the relevant information for each field can be found. Please move all other information to the respective sections of your revised manuscript and update/correct your checklist.

- In your Author Checklist, you have indicated (with a positive reply) that your study falls under dual use research restrictions, but we cannot find the relevant information in your manuscript. This issue needs to be clarified before acceptance of your manuscript.

- Please change the heading of your "Declaration of interests" statement to "Disclosure and competing interests statement".

- The author contributions statement should be removed from the manuscript file. Instead, we now use CRediT to specify the contributions of each author in the journal submission system. Please use the free text box to provide more detailed descriptions. See also our guide to authors:
<https://www.embopress.org/page/journal/14693178/authorguide#authorshipguidelines>.

- We noticed that callouts for Fig. 1E, 4D&G are missing; please make sure that all Figure panels are called out (in alphabetical order) in your revised manuscript.

- Please rename your Tables EV1-EV5 and EV7-EV10 as "Dataset EV#". Your Table EV6 should then be renamed as "Table EV1". All of them need their names and legends to be added to the respective Excel sheets, while the legends should be removed from the manuscript file. Please update the callouts of your Tables and Datasets accordingly throughout your manuscript.

- You have 7 EV Figures, but only a maximum of 5 can by typeset. The remaining 2 figures should be bundled together with their legends in a single PDF file called "Appendix", which should start with a short Table of Contents on its first page (including page numbers). Appendix figures should be referred to in the main text as: "Appendix Figure S1, Appendix Figure S2". See detailed instructions here:
<https://www.embopress.org/page/journal/14693178/authorguide#expandedview>.

- Please correct the heading "Methods" to "Materials and Methods".

- The manuscript sections are in the wrong order. Please restructure your manuscript following this order: Title page, Abstract, Keywords, Introduction, Results, Discussion, Materials and Methods, Data availability, Acknowledgements, Disclosure and competing interests statement, References, Figure legends, Expanded View Figure legends.

- Please note that EMBO press papers are accompanied online by:
A) a short (1-2 sentences) summary of the findings and their significance,
B) 2-4 short bullet points highlighting the key results, and
C) a synopsis image that is exactly 550 pixels wide and 300-600 pixels high (the height is variable). You can either show a model or key data in the synopsis image. Please note that the text needs to be readable at the final size.
Please upload this information along with your revised manuscript (the text for A and B should be provided in a separate Word file).

Please also note that as part of the EMBO publications' Transparent Editorial Process, EMBO reports publishes online a Review Process File to accompany accepted manuscripts. This File will be published in conjunction with your paper and will include the referee reports, your point-by-point response and all pertinent correspondence relating to the manuscript.

You can opt out of this by letting the editorial office know (emboreports@embo.org). If you do opt out, the Review Process File link will point to the following statement: "No Review Process File is available with this article, as the authors have chosen not to make the review process public in this case."

We would also welcome the submission of cover suggestions or motifs to be used by our Graphics Illustrator in designing a cover.

We look forward to seeing a final version of your manuscript as soon as possible.

Yours sincerely,

Ioannis Papaioannou, PhD
Editor
EMBO reports

Referee #1:

Good revision

Referee #2:

The authors have addressed my comments and I think the addition of the proteomic/acetylome data in Figure 3 strengthens this work. The additional controls included address most of my concerns.

Referee #3:

The revised manuscript is indeed improved. Authors did tackle my major concerns, although the mechanistic insight remains minimal and the energy storage phenotype is still a bit obscure.
However, I do believe the authors did enough and the current manuscript may be published in EMBO Reports.

Referee #4:

In the revised manuscript, the authors attempted to address the reviewer's comments by providing more evidence to support their conclusions. However, the major lacunae in the original submission of not providing adequate direct mechanistic insights behind the observed phenotype remains a concern in the revised manuscript.
The authors claim that chm exerts its function not only by regulating transcription but also via modulation of protein levels and changes in non-histone acetylation. The authors provide proteomic evidence to support this conclusion. However, how at the mechanistic level chm is causing these changes is unexplored and is left to speculation. In their response to the reviewer's comments, the authors state that they plan to focus on some of these substrates in future studies. However, I feel this should have been part of this manuscript.
More evidence is needed to demonstrate chm's direct role in regulating the transcriptome, proteome, and acetylome of flies to bring about the observed phenotypic changes.

All editorial and formatting issues were resolved by the authors.

Dr. Axel Imhof
Ludwig-Maximilians-Universität München
Biomedical Center
Großhadernerstr. 9
Planegg-Martinsried, Bayern 82152
Germany

Dear Dr. Imhof,

I am very pleased to accept your manuscript for publication in the next available issue of EMBO reports. Thank you for your contribution to our journal.

At the end of this email I include important information about how to proceed. Please ensure that you take the time to read the information and complete and return the necessary forms to allow us to publish your manuscript as quickly as possible.

As part of the EMBO publication's Transparent Editorial Process, EMBO reports publishes online a Review Process File to accompany accepted manuscripts. As you are aware, this File will be published in conjunction with your paper and will include the referee reports, your point-by-point response and all pertinent correspondence relating to the manuscript.

If you do NOT want this File to be published, please inform the editorial office within 2 days, if you have not done so already, otherwise the File will be published by default [contact: emboreports@embo.org]. If you do opt out, the Review Process File link will point to the following statement: "No Review Process File is available with this article, as the authors have chosen not to make the review process public in this case."

Thank you again for your contribution to EMBO reports and congratulations on a successful publication. Please consider us again in the future for your most exciting work.

Yours sincerely,

Ioannis Papaioannou, PhD
Editor
EMBO reports

****

THINGS TO DO NOW:

Please note that you will be contacted by Wiley Author Services to complete licensing and payment information. The required 'Page Charges Authorization Form' is available here: https://www.embopress.org/pb-assets/embo-site/er_apc.pdf - please download and complete the form and return to embopressproduction@wiley.com

EMBO Press participates in many Publish and Read agreements that allow authors to publish Open Access with reduced/no publication charges. Check your eligibility: https://authorservices.wiley.com/author-resources/Journal-Authors/open-access/affiliation-policies-payments/index.html

You will receive proofs by e-mail approximately 2-3 weeks after all relevant files have been sent to our Production Office; you should return your corrections within 2 days of receiving the proofs.

Please inform us if there is likely to be any difficulty in reaching you at the above address at that time. Failure to meet our deadlines may result in a delay of publication, or publication without your corrections.

All further communications concerning your paper should quote reference number EMBOR-2023-57023V3 and be addressed to emboreports@wiley.com.

Should you be planning a Press Release on your article, please get in contact with emboreports@wiley.com as early as possible, in order to coordinate publication and release dates.

EMBO Press Author Checklist

Corresponding Author Name: Axel Imhof
Journal Submitted to: EMBO Reports
Manuscript Number: EMBOR-2023-57023V2

USEFUL LINKS FOR COMPLETING THIS FORM
The EMBO Journal - Author Guidelines
EMBO Reports - Author Guidelines
Molecular Systems Biology - Author Guidelines
EMBO Molecular Medicine - Author Guidelines

Reporting Checklist for Life Science Articles (updated January
This checklist is adapted from Materials Design Analysis Reporting (MDAR) Checklist for Authors. MDAR establishes a minimum set of requirements in transparent reporting in the life sciences (see Statement of Task: 10.31222/osf.io/9sm4x). Please follow the journal's guidelines in preparing your manuscript.
Please note that a copy of this checklist will be published alongside your article.

Abridged guidelines for figures
1. Data
The data shown in figures should satisfy the following conditions:
- the data were obtained and processed according to the field's best practice and are presented to reflect the results of the experiments in an accurate and unbiased manner.
- ideally, figure panels should include only measurements that are directly comparable to each other and obtained with the same assay.
- plots include clearly labeled error bars for independent experiments and sample sizes. Unless justified, error bars should not be shown for technical
- if n<5, the individual data points from each experiment should be plotted. Any statistical test employed should be justified.
- Source Data should be included to report the data underlying figures according to the guidelines set out in the authorship guidelines on Data

2. Captions
Each figure caption should contain the following information, for each panel where they are relevant:
- a specification of the experimental system investigated (eg cell line, species name).
- the assay(s) and method(s) used to carry out the reported observations and measurements.
- an explicit mention of the biological and chemical entity(ies) that are being measured.
- an explicit mention of the biological and chemical entity(ies) that are altered/varied/perturbed in a controlled manner.
- the exact sample size (n) for each experimental group/condition, given as a number, not a range;
- a description of the sample collection allowing the reader to understand whether the samples represent technical or biological replicates (including how many animals, litters, cultures, etc.).
- a statement of how many times the experiment shown was independently replicated in the laboratory.
- definitions of statistical methods and measures:
  - common tests, such as t-test (please specify whether paired vs. unpaired), simple χ2 tests, Wilcoxon and Mann-Whitney tests, can be unambiguously identified by name only, but more complex techniques should be described in the methods section;
  - are tests one-sided or two-sided?
  - are there adjustments for multiple comparisons?
  - exact statistical test results, e.g., P values = x but not P values < x;
  - definition of 'center values' as median or average;
  - definition of error bars as s.d. or s.e.m.

Please complete ALL of the questions below.
Select "Not Applicable" only when the requested information is not relevant for your study.

Materials

Newly Created Materials	Information included in the manuscript?	In which section is the information available? (Reagents and Tools Table, Materials and Methods, Figures, Data Availability Section)
New materials and reagents need to be available; do any restrictions apply?	Not Applicable	

Antibodies	Information included in the manuscript?	In which section is the information available? (Reagents and Tools Table, Materials and Methods, Figures, Data Availability Section)
For **antibodies** provide the following information:<br>- Commercial antibodies: RRID (if possible) or supplier name, catalogue number and or/clone number<br>- Non-commercial: RRID or citation	Yes	Materials and methods

DNA and RNA sequences	Information included in the manuscript?	In which section is the information available? (Reagents and Tools Table, Materials and Methods, Figures, Data Availability Section)
**Short novel DNA or RNA including primers, probes**: provide the sequences.	Yes	Materials and methods

Cell materials	Information included in the manuscript?	In which section is the information available? (Reagents and Tools Table, Materials and Methods, Figures, Data Availability Section)
**Cell lines:** Provide species information, strain. Provide accession number in repository **OR** supplier name, catalog number, clone number, and/**OR** RRID.	Not Applicable	
**Primary cultures:** Provide species, strain, sex of origin, genetic modification status.	Not Applicable	
Report if the cell lines were recently **authenticated** (e.g., by STR profiling) and tested for mycoplasma contamination.	Not Applicable	

Experimental animals	Information included in the manuscript?	In which section is the information available? (Reagents and Tools Table, Materials and Methods, Figures, Data Availability Section)
**Laboratory animals or Model organisms:** Provide species, strain, sex, age, genetic modification status. Provide accession number in repository **OR** supplier name, catalog number, clone number, **OR** RRID.	Yes	Materials and methods
**Animal observed in or captured from the field:** Provide species, sex, and age where possible.	Not Applicable	
Please detail **housing and husbandry conditions**.	Yes	Materials and methods

Plants and microbes	Information included in the manuscript?	In which section is the information available? (Reagents and Tools Table, Materials and Methods, Figures, Data Availability Section)
**Plants:** provide species and strain, ecotype and cultivar where relevant, unique accession number if available, and source (including location for collected wild specimens).	Not Applicable	
**Microbes:** provide species and strain, unique accession number if available, and source.	Not Applicable	

Human research participants	Information included in the manuscript?	In which section is the information available? (Reagents and Tools Table, Materials and Methods, Figures, Data Availability Section)
If collected and within the bounds of privacy constraints report on age, sex and gender or ethnicity for all study participants.	Not Applicable	

Core facilities	Information included in the manuscript?	In which section is the information available? (Reagents and Tools Table, Materials and Methods, Figures, Data Availability Section)
If your work benefited from core facilities, was their service mentioned in the acknowledgments section?	Yes	Acknowledgements

Design

Study protocol	Information included in the manuscript?	In which section is the information available? (Reagents and Tools Table, Materials and Methods, Figures, Data Availability Section)
If study protocol has been **pre-registered, provide DOI in the manuscript**. For clinical trials, provide the trial registration number **OR** cite DOI.	Not Applicable	
Report the **clinical trial registration number** (at ClinicalTrials.gov or equivalent), where applicable.	Not Applicable	

Laboratory protocol	Information included in the manuscript?	In which section is the information available? (Reagents and Tools Table, Materials and Methods, Figures, Data Availability Section)
Provide DOI OR other citation details if **external detailed step-by-step protocols** are available.	Not Applicable	

Experimental study design and statistics	Information included in the manuscript?	In which section is the information available? (Reagents and Tools Table, Materials and Methods, Figures, Data Availability Section)
Include a statement about **sample size** estimate even if no statistical methods were used.	Yes	Figure legends
Were any steps taken to minimize the effects of subjective bias when allocating animals/samples to treatment (e.g. **randomization procedure**)? If yes, have they been described?	Yes	1. Flies of different generations were used; 2. Flies were randomly arranged or ordered for each replicate or experiment; 3. Flies from different vials were used; 4. In some cases, experiments were blinded (esp. starvation assays)
Include a statement about **blinding** even if no blinding was done.	Yes	In some cases, experiments were blinded (esp. starvation assays)
Describe **inclusion/exclusion criteria** if samples or animals were excluded from the analysis. Were the criteria pre-established?<br><br>If sample or data points were omitted from analysis, report if this was due to attrition or intentional exclusion and provide justification.	Yes	Samples were removed or excluded based on PCA. In those experimental designs where samples were paired, all samples/replicate from that paired condition was removed in cases where the trend is different from other paired replicates.
For every figure, are **statistical tests** justified as appropriate? Do the data meet the assumptions of the tests (e.g., normal distribution)? Describe any methods used to assess it. Is there an estimate of variation within each group of data? Is the variance similar between the groups that are being statistically compared?	Yes	Yes, statistical tests were appropriate. QQ-plot, density plot, shapiro-wilk test and others were performed. Tests were decided based on the observations from all these. Type of test and in multi-group analysis, corresponding post-hoc test was performed.

Sample definition and in-laboratory replication	Information included in the manuscript?	In which section is the information available? (Reagents and Tools Table, Materials and Methods, Figures, Data Availability Section)
In the figure legends: state number of times the experiment was **replicated** in laboratory.	Yes	Figure legends
In the figure legends: define whether data describe **technical or biological replicates**.	Yes	Figure legends

Ethics

Ethics	Information included in the manuscript?	In which section is the information available? (Reagents and Tools Table, Materials and Methods, Figures, Data Availability Section)
Studies involving **human participants**: State details of **authority granting ethics approval** (IRB or equivalent committee(s), provide reference number for approval.	Not Applicable	
Studies involving **human participants**: Include a statement confirming that **informed consent** was obtained from all subjects and that the experiments conformed to the principles set out in the WMA Declaration of Helsinki and the Department of Health and Human Services Belmont Report.	Not Applicable	
Studies involving **human participants:** For publication of **patient photos**, include a statement confirming that consent to publish was obtained.	Not Applicable	
Studies involving experimental **animals**: State details of **authority granting ethics approva**l (IRB or equivalent committee(s), provide reference number for approval. Include a statement of compliance with ethical regulations.	Not Applicable	
Studies involving **specimen and field samples:** State if relevant **permits** obtained, provide details of authority approving study; if none were required, explain why.	Not Applicable	

Dual Use Research of Concern (DURC)	Information included in the manuscript?	In which section is the information available? (Reagents and Tools Table, Materials and Methods, Figures, Data Availability Section)
Could your study fall under dual use research restrictions? Please check biosecurity documents and list of **select agents and toxins** (CDC): https://www.selectagents.gov/sat/list.htm	Not Applicable	
If you used a select agent, is the security level of the lab appropriate and reported in the manuscript?	Not Applicable	
If a study is subject to dual use research of concern regulations, is the name of the **authority granting approval and reference number** for the regulatory approval provided in the manuscript?	Not Applicable	

Reporting

The MDAR framework recommends adoption of discipline-specific guidelines, established and endorsed through community initiatives. Journals have their own policy about requiring specific guidelines and recommendations to complement MDAR.

Adherence to community standards	Information included in the manuscript?	In which section is the information available? (Reagents and Tools Table, Materials and Methods, Figures, Data Availability Section)
State if relevant guidelines or checklists (e.g., **ICMJE, MIBBI, ARRIVE, PRISMA**) have been followed or provided.	Not Applicable	
For **tumor marker prognostic studies,** we recommend that you follow the **REMARK** reporting guidelines (see link list at top right). See author guidelines, under 'Reporting Guidelines'. Please confirm you have followed these guidelines.	Not Applicable	
For **phase II and III randomized controlled trials**, please refer to the **CONSORT** flow diagram (see link list at top right) and submit the CONSORT checklist (see link list at top right) with your submission. See author guidelines, under 'Reporting Guidelines'. Please confirm you have submitted this list.	Not Applicable	

Data Availability

Data availability	Information included in the manuscript?	In which section is the information available? (Reagents and Tools Table, Materials and Methods, Figures, Data Availability Section)
Have **primary datasets** been deposited according to the journal's guidelines (see 'Data Deposition' section) and the respective accession numbers provided in the Data Availability Section?	Yes	Data availability
Were **human clinical and genomic datasets** deposited in a public access-controlled repository in accordance to ethical obligations to the patients and to the applicable consent agreement?	Yes	Data availability
Are **computational models** that are central and integral to a study available without restrictions in a machine-readable form? Were the relevant accession numbers or links provided?	Not Applicable	
If publicly available data were reused, provide the respective **data citations in the reference list**.	Not Applicable	
